# Design, Synthesis, In Silico and In Vitro Pharmacological Profiling of Cannabidiol-like Synthetic Analogues as Multi-Target Anti-Alzheimer’s Agents

**DOI:** 10.3390/molecules31152657

**Published:** 2026-07-30

**Authors:** Boris P. Stoyanov, Borislav Georgiev, Denitsa Stefanova, Virginia Tzankova, Elena Kalcheva-Yovkova, Nikolay Vassilev, Miroslav Rangelov, Nadezhda Todorova, Dimitrina Zheleva-Dimitrova, Boris Shivachev, Violina T. Angelova

**Affiliations:** 1 Department of Pharmacology, Pharmacotherapy and Toxicology, Faculty of Pharmacy, Medical University of Sofia, 1000 Sofia, Bulgaria; borisstoyanov03@gmail.com (B.P.S.); denitsa.stefanova@pharmfac.mu-sofia.bg (D.S.); virginia_tzankova@yahoo.com (V.T.); 2 Institute of Biodiversity and Ecosystem Research, Bulgarian Academy of Sciences, 1113 Sofia, Bulgaria; bobogeorgiev5@gmail.com (B.G.); nadeshdahr@gmail.com (N.T.); 3 Faculty of Computer Systems and Technologies, Technical University–Sofia, 1000 Sofia, Bulgaria; elena@tu-sofia.bg; 4 Institute of Organic Chemistry with Centre of Phytochemistry, Bulgarian Academy of Sciences, 1113 Sofia, Bulgaria; nikolay.vassilev@orgchm.bas.bg (N.V.); marangelov@gmail.com (M.R.); 5 Department of Pharmacognosy, Faculty of Pharmacy, Medical University of Sofia, 1000 Sofia, Bulgaria; dzheleva@pharmfac.mu-sofia.bg; 6 Institute of Mineralogy and Crystallography “Acad. Ivan Kostov”, Bulgarian Academy of Sciences, 1113 Sofia, Bulgaria; blshivachev@gmail.com; 7 Department of Chemistry, Faculty of Pharmacy, Medical University of Sofia, 1000 Sofia, Bulgaria

**Keywords:** Alzheimer’s disease, cannabidiol analogues, hydrazones, selective BChE inhibitors, antioxidant activity, molecular docking, neurotoxicity, lipid peroxidation, multitarget-directed anti-Alzheimer agents

## Abstract

Alzheimer’s disease (AD) is a multifactorial neurodegenerative disorder that requires therapeutic agents capable of targeting multiple pathological pathways. In this study, a series of cannabidiol (CBD)-like hydrazone derivatives (**3a**–**i**) was synthesized and characterized by NMR, HRMS, and single-crystal X-ray diffraction for compound **3i**. In silico ADME analysis predicted favorable drug-like properties, including compliance with Lipinski’s Rule of Five, oral bioavailability, and blood–brain barrier permeability. The compounds were evaluated for cholinesterase inhibition, antioxidant activity, cytotoxicity in neuronal cell lines, and binding interactions with human butyrylcholinesterase (hBChE) by molecular docking. Biological evaluation revealed a marked preference for BChE over acetylcholinesterase (AChE). Compound **3f** was the most potent BChE inhibitor (IC_50_ = 1.67 ± 0.11 μM), while compounds **3b** and **3f** demonstrated high selectivity toward BChE. Antioxidant assays (DPPH, ABTS, FRAP, and FTC) indicated moderate, mechanism-dependent activity. Compounds **3b** and **3e** showed the strongest ABTS radical-scavenging effects, whereas compounds **3b** and **3f** provided the greatest protection against lipid peroxidation, surpassing CBD under the tested conditions. Several derivatives, particularly **3a**, **3b**, **3f**, **3h**, and **3i**, exhibited favorable safety profiles in SH-SY5Y and Neuro-2a cells. Molecular docking supported the experimental findings. Overall, compounds **3b** and **3f** emerged as promising multifunctional leads for the development of multitarget-directed anti-Alzheimer agents.

## 1. Introduction

Alzheimer’s disease (AD) is a multifactorial neurodegenerative disorder characterized by cholinergic dysfunction, amyloid-β and tau aggregation, oxidative stress, mitochondrial dysfunction, metal dyshomeostasis, and neuroinflammation, ultimately leading to neuronal loss and cognitive decline [1,2]. Owing to this complex pathology, single-target therapies have demonstrated limited efficacy and mainly provide symptomatic relief [2,3]. Consequently, multitarget-directed ligands (MTDLs) have emerged as a promising strategy for simultaneously modulating multiple pathogenic pathways [4]. Recently approved anti-amyloid monoclonal antibodies, including Aducanumab [5], Lecanemab [6], and Donanemab [7], represent the first disease-modifying therapies for AD, reducing amyloid burden and modestly slowing cognitive decline in early-stage patients [8]. However, their clinical benefit remains limited and is frequently accompanied by amyloid-related imaging abnormalities (ARIA), high treatment costs, and restricted applicability [9,10,11]. These limitations further emphasize the need for alternative multitarget therapeutic approaches for Alzheimer’s disease [12].

**Figure 1:**
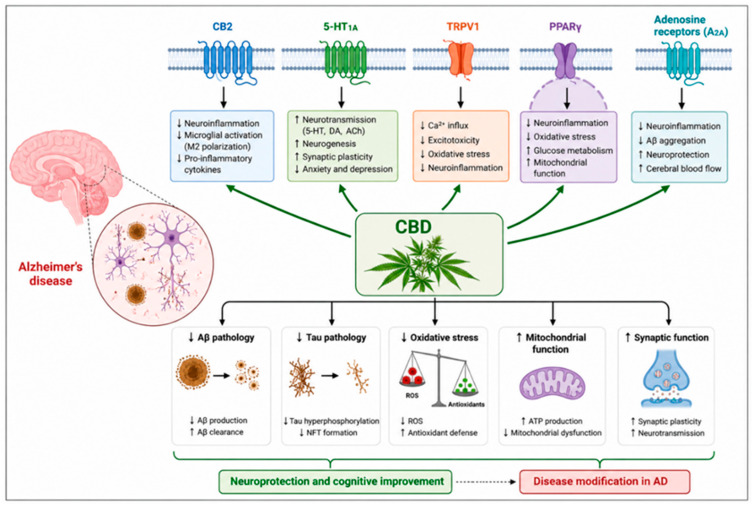
Proposed multitarget neuroprotective mechanisms underlying the rationale for CBD-like structures design in Alzheimer’s disease.

Cannabidiol (CBD), a non-psychoactive phytocannabinoid derived from Cannabis sativa, has emerged as a promising multitarget neuroprotective agent for Alzheimer’s disease (AD) [13,14,15]. CBD and its analogues have been reported to modulate several AD-associated pathological pathways, including β-amyloid toxicity, tau hyperphosphorylation, oxidative stress, mitochondrial dysfunction, and neuroinflammation [16,17,18,19,20,21,22,23,24,25,26,27,28]. In particular, CBD has been shown to reduce reactive oxygen species production, regulate GSK-3β activity, improve neuronal viability, and promote neuroprotective microglial responses [13,16,29,30,31,32]. Furthermore, in vivo studies in 5xFAD mice demonstrated that CBD administration improves cognitive performance and attenuates neuroinflammatory responses, supporting its potential as a neuroprotective scaffold for AD-oriented drug design [33,34,35,36] (Figure 1).

Despite its therapeutic potential, the clinical application of CBD remains limited by poor oral bioavailability [37], high lipophilicity [38], rapid metabolic degradation [39], and modest target selectivity [40]. Therefore, rational structural modification of the CBD scaffold has gained increasing attention as a strategy to improve pharmacological activity and target engagement. In this regard, hybrid molecular design incorporating different pharmacophores represents a promising approach for the development of novel multitarget anti-Alzheimer agents [4,41,42,43,44]. Among the various pharmacophoric motifs used in MTDL design, hydrazone-based fragments have received significant attention due to their synthetic accessibility, structural versatility, metal-chelating capacity, redox properties, and ability to establish diverse intermolecular interactions with biological targets [45,46]. Importantly, hydrazone-containing compounds have demonstrated promising activities against several AD-related pathological processes, including cholinesterase inhibition, oxidative stress, metal dysregulation, and amyloid aggregation [47]. Moreover, the incorporation of hydrazone functionalities into cannabinoid-inspired scaffolds may enhance cholinesterase affinity while preserving the intrinsic neuroprotective and antioxidant properties associated with CBD-derived structures.

Recent studies on CBD-based terpene–*N*-acylhydrazone analogues suggest that incorporation of terpene fragments and the *N*-acylhydrazone pharmacophore represents a promising approach for generating multifunctional cannabinoid-inspired molecules with improved pharmacological potential [48,49,50,51]. The first generation of terpene-cinnamoyl-acylhydrazone derivatives exhibited potent anti-inflammatory and antinociceptive activities, with several compounds displaying effects comparable to morphine in experimental pain models [48]. These findings suggest an improved capacity to modulate pathways associated with inflammation, oxidative stress, and cellular homeostasis, which are also implicated in AD pathogenesis [48].

The therapeutic relevance of BChE inhibition in Alzheimer’s disease has gained increasing attention due to the alterations in cholinesterase activity during disease progression. Unlike AChE, whose activity may decline in advanced stages of AD [52], BChE activity is relatively preserved or increased and may represent an important contributor to acetylcholine hydrolysis under pathological conditions [53]. Consequently, BChE-selective inhibitors have emerged as promising candidates for maintaining cholinergic signaling and represent an important direction in anti-Alzheimer drug discovery. Despite increasing interest in cannabinoid-derived MTDLs [54,55], CBD-like hydrazone derivatives with selective BChE inhibitory activity remain insufficiently explored.

Based on these considerations, the present study describes the design, synthesis, and in vitro biological evaluation of a series of CBD-like derivatives (**3a**–**i**) based on selected structural features of cannabidiol as potential multitarget anti-Alzheimer agents. We hypothesized that combining a terpenoid scaffold with aroyl or heteroaroyl hydrazone pharmacophoric fragments [45] could generate multifunctional ligands with enhanced BChE selectivity and improved multitarget anti-Alzheimer potential. The synthesized compounds were evaluated for their cholinesterase inhibitory activity, with particular emphasis on BChE selectivity, together with their antioxidant activity, cytotoxicity, and in silico ADME/toxicity profiles. By integrating these complementary structural elements, the present study explores a rational MTDL design strategy for the development of multifunctional neuroprotective agents targeting multiple pathological mechanisms associated with Alzheimer’s disease, including cholinergic dysfunction.

## 2. Results and Discussion

### 2.1. Design, Synthesis and Structural Characterization

The molecular design strategy was based on the incorporation of a cannabidiol (CBD)-like terpenoid scaffold and biologically relevant aroyl- and heteroaroyl hydrazone fragments to generate novel multifunctional derivatives for the treatment of Alzheimer’s disease (AD). The synthesized compounds were rationally designed to combine several pharmacologically desirable properties within a single molecular framework, including cholinesterase inhibition (AChE/BChE), antioxidant potential, favorable blood–brain barrier (BBB) permeability, and improved drug-like characteristics. The design concept relied on the integration of two complementary structural motifs: (i) a carvone-derived terpenoid scaffold incorporating selected structural features of cannabidiol, providing a lipophilic molecular framework, and (ii) aroyl- or heteroaroyl hydrazone fragments, which were introduced as biologically relevant structural elements expected to contribute to the multitarget pharmacological profile of the synthesized derivatives. The incorporation of differently substituted aromatic and heteroaromatic hydrazide fragments enabled modulation of the electronic properties, lipophilicity, hydrogen-bonding capacity, and steric profile of the final compounds, thereby allowing fine-tuning of their predicted biological properties and CNS drug-like behavior.

**Figure 2:**
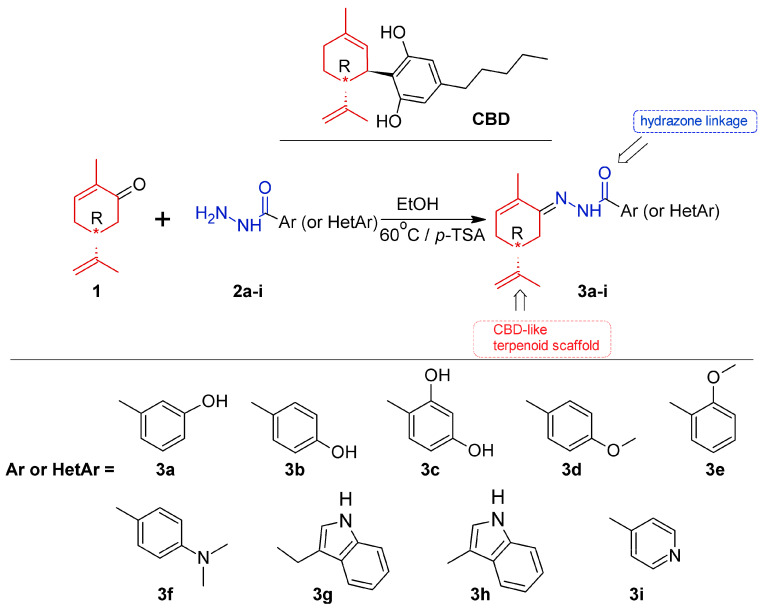
Synthetic strategy for the preparation of CBD-like carvone derivatives containing aroyl- or heteroaroyl hydrazone fragments, **3a**-**i**. The red-highlighted region represents the CBD-like terpenoid scaffold, whereas the blue-highlighted region corresponds to the introduced aroyl- or heteroaroyl hydrazone fragment. The aroyl and heteroaroyl structural fragments are shown in black. The asterisk (*) indicates the chiral center with an R configuration.

The target compounds **3a**–**i** were synthesized via condensation of the corresponding hydrazides (**2a**–**i**) with carvone (**1**) in ethanol in the presence of catalytic *p*-toluenesulfonic acid (*p*-TSA, 1 mol%). The catalytic role of *p*-TSA is attributed to the acid-mediated activation of the carbonyl group, facilitating nucleophilic addition of the hydrazide and subsequent condensation toward hydrazone formation, while also shortening the reaction time and improving the efficiency of the synthetic process. The reaction progress was monitored by thin-layer chromatography (TLC), and the reactions were carried out at 60 °C for 8–12 h, affording the desired hydrazone derivatives in moderate to excellent yields (Figure 2). The reaction conditions favored the predominant formation of the *E*-configured hydrazones, as supported by the NMR spectroscopic data.

The absolute configuration of the stereogenic center was assigned as R based on the use of naturally occurring (R)-carvone as the starting material. Since the synthetic transformations involved condensation reactions without modification of the stereogenic center or conditions promoting racemization, the original configuration was retained throughout the synthesis. The observed NMR coupling patterns and chemical shift data were fully consistent with the preserved stereochemical arrangement of the molecules.

The structures of the synthesized compounds were fully confirmed by HRMS together with one- and two-dimensional NMR spectroscopy, including ^1^H, ^13^C, DEPT-135, COSY, HSQC, and HMBC experiments. All compounds displayed characteristic spectroscopic features consistent with the proposed structures and the formation of the hydrazone linkage. In the ^1^H NMR spectra, the singlets corresponding to the exocyclic methylene group (=CH_2_) were consistently observed at δ 4.76–4.84 ppm, while the olefinic proton H-3 of the cyclohexene fragment appeared at δ 6.14–6.27 ppm. The methyl groups attached to the cyclohexene scaffold resonated as singlets at δ 1.73–1.92 ppm. The NH proton of the hydrazone moiety was detected as a singlet in the downfield region at δ 10.06–11.70 ppm, confirming the presence of the hydrazide fragment. Hydroxyl-containing derivatives **3a**–**c** exhibited additional broad singlets assigned to phenolic OH groups in the region δ 10.01–11.75 ppm. The ^13^C NMR spectra further supported the assigned structures. Signals for the exocyclic methylene carbon atoms were observed around δ 109–110 ppm, whereas the imine carbon (C=N) resonated in the range δ 149–159 ppm. The carbonyl carbon signals of the hydrazide fragment appeared at δ 161–173 ppm, depending on the electronic nature of the aromatic substituent. Aromatic methoxy substituents in compounds **3d** and **3e** produced characteristic resonances at δ 55.37 and 56.24 ppm, respectively, while the dimethylamino derivative **3f** showed the expected N(CH_3_)_2_ signal at δ 2.98 ppm in the ^1^H NMR spectrum. Several compounds, namely **3e**, **3g**, and **3i**, existed in solution as mixtures of *synperiplanar* and *antiperiplanar* conformers around the amide bond, as evidenced by duplicated signals in both ^1^H and ^13^C NMR spectra. The conformational behavior was most pronounced for the indole-containing derivative **3g**, which displayed a 1:0.64 conformer ratio in DMSO-*d*_6_. Such conformational equilibria are characteristic of hydrazone systems and are attributed to restricted rotation around the amide bond. The indole derivatives **3g** and **3h** exhibited characteristic aromatic proton patterns in the δ 6.9–8.4 ppm region together with NH signals at δ 10.84 and 11.70 ppm, confirming incorporation of the indole scaffold. In the pyridyl derivative **3i**, the aromatic pyridine protons appeared as two doublets at δ 7.73 and 8.73 ppm, consistent with a para-substituted pyridine ring.

Additional structural confirmation was obtained by a combination of DEPT-135 and two-dimensional NMR experiments (COSY, HSQC, and HMBC), which enabled unambiguous assignment of all proton and carbon resonances in compounds. The DEPT-135 spectra allowed differentiation of CH, CH_2_, and CH_3_ groups within the cyclohexene scaffold and the aromatic substituents, confirming the presence of two methyl groups, methylene carbons, and the exocyclic methylene fragment. In particular, the negative-phase signals observed for the CH_2_ carbons clearly confirmed the assignments of C-4, C-6, and the exocyclic =CH_2_ group. The ^1^H–^1^H COSY spectra established the proton spin systems within the cyclohexene ring through characteristic correlations between H-4/H-5 and H-5/H-6, thereby confirming the connectivity of the alicyclic fragment. Additional COSY cross-peaks within the aromatic regions verified the substitution patterns of the phenyl, pyridyl, and indole moieties. The HSQC spectra enabled direct one-bond ^1^H–^13^C correlations and allowed unequivocal assignment of all protonated carbons. Correlations between the exocyclic methylene protons and the carbon signal at approximately δ 109–110 ppm, as well as between the olefinic H-3 proton and the corresponding sp^2^ carbon, confirmed the integrity of the terpene-derived fragment. Long-range ^1^H–^13^C correlations observed in the HMBC spectra provided decisive evidence for the formation of the hydrazone linkage. Key HMBC cross-peaks between the NH proton and the imine carbon (C=N), as well as correlations from H-3 and neighboring methylene protons to the azomethine carbon, confirmed the connectivity between the cyclohexene scaffold and the hydrazide moiety. Additional HMBC correlations from aromatic protons to the carbonyl carbon verified the attachment and substitution pattern of the aromatic fragments. High-resolution mass spectrometry analysis confirmed the molecular compositions of all synthesized compounds. The experimentally observed [M + H]^+^ ions were in excellent agreement with the calculated values, with deviations below 5 ppm, confirming the purity and identity of the target hydrazones.

### 2.2. Single-Crystal X-Ray Diffraction Analysis of Compound ***3i***

**Figure 3:**
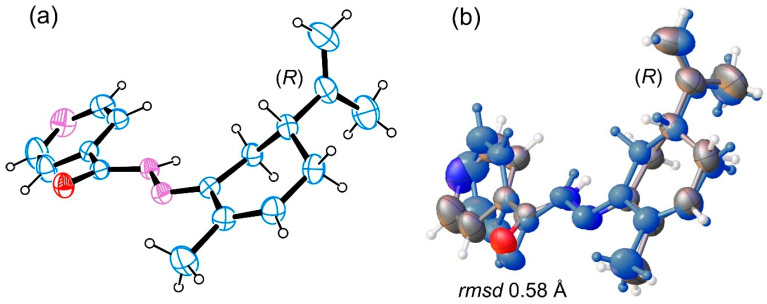
Molecular structure of (**a**) one of the molecules present in the asymmetric unit of **3i**, showing the assigned (R) configuration at the stereogenic center and (**b**) overlay of the two crystallographically independent molecules in the asymmetric unit, showing their close conformational similarity with an rmsd of 0.58 Å.

**Figure 4:**
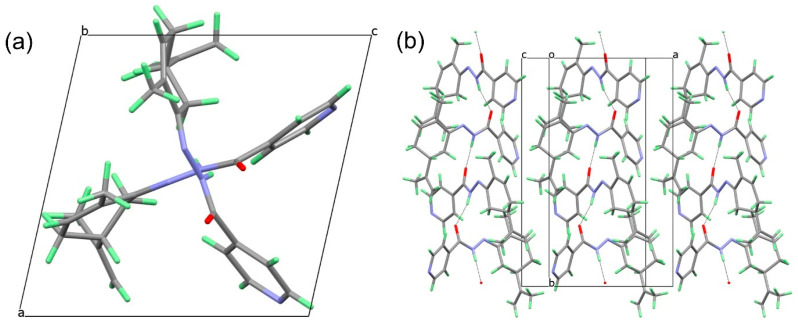
(**a**) A view of the two crystallographically independent molecules of **3i** along b axis disclosing the inner side. (**b**) Crystal packing of **3i** propagating along α axis; **3i** molecules are linked through intermolecular N–H•••O hydrogen bonds (dashed lines) between the hydrazide N–H groups and carbonyl oxygen atoms, generating one-dimensional chains that propagate parallel to the crystallographic β axis.

The crystal structure of **3i** crystallizes in the non-centrosymmetric monoclinic space group P2_1_ with Z = 4, indicating the presence of two crystallographically independent molecules (Z′ = 2) in the asymmetric unit. The absolute structure parameter [Flack = 0.1(6)] is consistent with the assigned (R) configuration at the stereogenic carbon atom. The molecule consists of a chiral bornane-derived bicyclic framework linked through a hydrazone-type spacer to a pyridyl amide fragment. Bond distances within the hydrazone moiety are characteristic of conjugated imine systems, with C=N bond lengths of 1.287(4) Å (N101–C111) and 1.291(4) Å (N102–C112), while the N–N bonds [1.395(3) and 1.388(3) Å] confirm substantial π-delocalization along the N–N=C fragment. The carbonyl groups exhibit normal amide-type distances [C71 = O81 1.228(3) Å, C72 = O82 1.228(3) Å]. The pyridyl and hydrazone fragments are nearly coplanar, as shown by the small torsion angles N91–N101–C111–C121 = −175.6(3)°, N92–N102–C112–C122 = −172.6(3)°, N101–N91–C71–O81 = 7.2(4)°, N102–N92–C72–O82 = 3.9(5)°, indicating an extended conjugated system. The stereogenic carbon atom within the bicyclic fragment adopts the R configuration, consistent with the refinement of the absolute structure. The rigid bicyclic framework forces the pyridyl–hydrazone substituent into a defined three-dimensional arrangement while preserving near planarity of the conjugated hydrazone-amide fragment. The asymmetric unit contains two conformationally similar molecules (Figure 3). Superposition of these independent molecules gives an RMS deviation of approximately 0.58 Å, indicating that they adopt closely related conformations despite minor differences in the orientation of the flexible bicyclic substituents. The packing is therefore governed primarily by the robust N–H•••O hydrogen bonds involving the hydrazide N–H donors and carbonyl oxygen acceptors of neighbouring molecules. These nearly linear interactions link neighbouring molecules into one-dimensional hydrogen-bonded chains running parallel to the crystallographic b axis, as observed in Figure 4b. Several weaker C–H•••O and C–H•••N contacts further reinforce the packing (Table S1). Together, these interactions generate a continuous supramolecular pseudo-layered framework in which the molecules are aligned along b (Figure 4a), while the bulky bicyclic substituents project outward from the hydrogen-bonded backbone (Figure 4b).

### 2.3. In Silico Studies

#### 2.3.1. ADMET Prediction

The newly synthesized compounds were subjected to an in silico ADME evaluation to predict their physicochemical characteristics, pharmacokinetic behavior, and drug-likeness properties. The SwissADME [56] online platform developed by the Swiss Bioinformatics Institute (https://www.swissadme.ch/, accessed on 18 June 2026) was used for the computational analysis. Molecular descriptors, physicochemical parameters, and pharmacokinetic properties were calculated. Drug-likeness and potential oral bioavailability were assessed using established rule-based filters, including Lipinski’s rule of five. The calculated molecular descriptors of the synthesized derivatives (**3a**–**i**) and cannabidiol (CBD), used as a reference compound, are summarized in Table 1.

The evaluated parameters included molecular weight (MW), molecular refractivity (MR), topological polar surface area (TPSA), number of heavy atoms and aromatic heavy atoms, rotatable bonds, hydrogen bond acceptors (HBA), hydrogen bond donors (HBD), calculated lipophilicity (logP), and Lipinski rule-of-five (R5) compliance.

The synthesized compounds displayed favorable molecular sizes, with MW values ranging from 269.34 to 337.42 Da, which are within the range generally considered suitable for drug-like molecules and CNS-active compounds. The TPSA values varied from 44.70 to 81.92 Å^2^, indicating an acceptable balance between polarity and membrane permeability. In particular, the moderate TPSA values observed for most derivatives may contribute to improved passive diffusion and potential CNS exposure, whereas the higher polarity of compound **3c** may explain its lower predicted BBB permeability. The number of rotatable bonds ranged from 4 to 5, indicating relatively limited molecular flexibility compared with CBD (6 rotatable bonds). The hydrogen bonding profile was favorable, with most derivatives containing 2–3 hydrogen bond acceptors and 1–2 hydrogen bond donors. Lower HBD values observed for several derivatives, particularly compound **3f**, may contribute to enhanced membrane permeability and CNS penetration. The calculated logP values ranged from 3.49 to 4.80, suggesting moderate lipophilicity. This range is considered favorable for CNS drug candidates, as it may support BBB penetration while avoiding excessive hydrophobicity that can negatively affect solubility and metabolic behavior. In contrast, CBD exhibited higher lipophilicity (logP = 5.85), resulting in one Lipinski rule-of-five violation. The transdermal permeability coefficient logKp is in the typical range of −2 to −6 cm/s (in this case around −5.5), which allows easier penetration through the skin [57].

All synthesized compounds complied with Lipinski’s rule of five, suggesting favorable drug-like characteristics and potential oral bioavailability. The predicted ADME-related properties, including aqueous solubility, gastrointestinal absorption, BBB permeability, P-glycoprotein substrate activity, CYP450 inhibition potential, membrane permeability, and synthetic accessibility, are presented in Table 2.

Most synthesized derivatives were predicted to be soluble, while compounds **3f**, **3g**, and **3h** showed moderate solubility, similarly to CBD. All tested compounds demonstrated high predicted gastrointestinal absorption, suggesting favorable oral absorption characteristics. Regarding CNS availability, most derivatives showed predicted BBB permeability, with the exception of compound **3c**. The favorable BBB profile of compounds **3f**, **3g**, and **3i** indicates their potential ability to reach the central nervous system, which is particularly relevant for the development of anti-Alzheimer’s agents. According to the BOILED-Egg model, the majority of compounds were located within the region associated with passive gastrointestinal absorption and BBB permeation. Moreover, all synthesized derivatives were predicted to be non-substrates of P-glycoprotein, suggesting a reduced probability of active efflux transport and improved CNS retention. The predicted membrane permeability values (logKp) ranged from −5.36 to −5.95 cm/s, indicating acceptable passive permeability characteristics [57]. The lowest permeability value was observed for compound **3i**, while compound **3f** displayed the highest predicted permeability among the synthesized derivatives. Drug–drug interactions are frequently associated with modulation of cytochrome P450 enzymes, which are responsible for the metabolism of the majority of clinically used drugs. The SwissADME platform predicts the inhibition potential of major CYP isoforms using machine-learning-based models [56]. The obtained results showed that several compounds may interact with CYP2C19, CYP2C9, and CYP3A4. Among these enzymes, CYP3A4 is particularly important because it participates in the metabolism of numerous therapeutic agents [58,59]. Therefore, potential CYP inhibition should be experimentally validated during further development. Notably, compound **3i** exhibited the most favorable predicted CYP profile, showing no predicted inhibition of CYP1A2 or CYP3A4. In comparison, compounds with predicted CYP3A4 inhibition may require additional metabolic evaluation to exclude possible drug–drug interaction risks. The synthetic accessibility scores of the synthesized derivatives ranged from 3.75 to 3.95, indicating relatively low synthetic complexity and feasible preparation.

**Figure 5:**
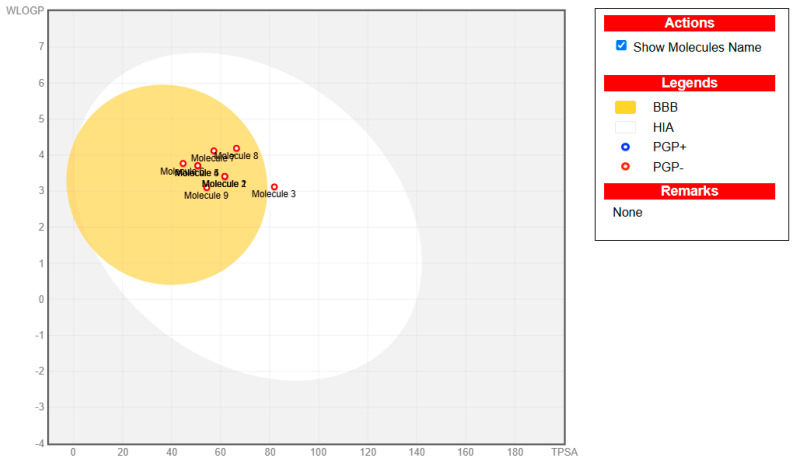
A BOILED-EGG diagram. Molecules **1**–**9** correspond to compounds **3a**–**i**, respectively.

Overall, the ADME evaluation suggests that compound **3f** represents the most balanced candidate, combining favorable physicochemical properties, high predicted BBB permeability, high gastrointestinal absorption, absence of P-glycoprotein substrate activity, and acceptable synthetic accessibility. Compound **3i** also demonstrated a promising profile, particularly due to its high BBB permeability, improved predicted metabolic profile, and lower overall toxicity risk. The BOILED-EGG model in Figure 5 predicts that the compounds are suitable for the synthesis of the required dosage forms, as all are predicted to successfully cross the blood–brain barrier (BBB) and with a high probability of penetrating the central nervous system. The negative permeability (PGP-, in red) of all proposed compounds in this series is also clearly visible, suggesting favorable pharmacokinetics and potential for good accumulation in the brain.

Table 3 presents the predicted toxicity parameters of compounds **3a**–**i** obtained using the ProTox-III web server (accessed on 18 June 2026). All compounds were predicted to belong to toxicity class 4, indicating low acute toxicity. The estimated LD_50_ values ranged from 324 to 510 mg/kg. Compounds **3a**–**e** exhibited identical LD_50_ values of 324 mg/kg, while compounds **3f** and **3h** showed slightly higher values. Compound **3g** displayed an intermediate LD_50_ value of 341 mg/kg, whereas compound **3i** exhibited the highest predicted LD_50_ value (510 mg/kg), suggesting the lowest level of acute toxicity within the series. Several toxicity endpoints, including neurotoxicity and respiratory toxicity, were predicted as active for most compounds, with probability values generally ranging from 0.50 to 0.75. In contrast, cardiotoxicity was predicted to be inactive for all derivatives, indicating a low likelihood of cardiotoxic effects. Hepatotoxicity was predicted for compounds **3a**–**e**, **3h**, and **3i**, whereas compounds **3f** and **3g** were predicted to be inactive for this endpoint. Nephrotoxicity was predicted only for compound **3c**, while immunotoxicity was predicted solely for compound **3h**. Regarding genotoxicity-related endpoints, compounds **3c**, **3g**, **3h**, and **3i** were predicted to be inactive for both mutagenicity and cytotoxicity, suggesting a more favorable safety profile compared to the remaining analogues. Furthermore, ecotoxicity was predicted to be inactive for compounds **3c** and **3i**, whereas the other derivatives showed only moderate probabilities of activity. Predicted hepatotoxicity was observed for most derivatives, suggesting that metabolic stability and potential hepatic liability should be further evaluated experimentally.

The BBB-barrier endpoint, which reflects blood–brain barrier permeability, revealed that compounds **3f**, **3g**, and **3i** possessed the highest probabilities of crossing the BBB, with values of 0.87, 0.82, and 0.90, respectively. These findings suggest the potential for central nervous system exposure and support the potential applicability of these compounds for the treatment of neurodegenerative disorders. Among them, compound **3i** demonstrated the highest predicted BBB permeability, making it a potential candidate for CNS-targeted applications. Although **3g** showed favorable BBB penetration, its higher predicted neurotoxicity warrants caution. Evaluation of cytochrome P450 interactions indicated generally low predicted activity toward most CYP isoforms. All compounds were predicted to be inactive toward CYP2E1, with a high confidence level (probability = 0.99). Likewise, CYP1A2, CYP2C19, CYP2D6, and CYP3A4 were predominantly predicted as inactive across the series, suggesting a relatively low risk of CYP-mediated drug–drug interactions. However, CYP2C9 inhibition was predicted for most compounds, although with relatively low probability values (0.51–0.64), indicating that this isoform may require additional attention during further optimization and biological evaluation.

Overall, compound **3f** demonstrated the most balanced predicted ADMET profile, combining high BBB permeability with low predicted organ toxicity, favorable CYP inhibition profile, and absence of major cytotoxicity alerts. Compound **3i** showed the highest predicted LD50 value and excellent BBB penetration, may indicate improved general safety; however, the predicted hepatotoxicity alert may require further evaluation. Compound **3g**, despite favorable BBB penetration, showed increased predicted neurotoxicity and CYP inhibition potential, suggesting a less desirable safety profile. However, the predicted hepatotoxicity and carcinogenicity alerts observed across the series highlight the necessity for further experimental validation. It should be noted that some differences between SwissADME and ProTox-III CYP predictions may be attributed to the use of different machine-learning models and prediction algorithms. SwissADME estimates CYP inhibition probability based on structural similarity and classification models, whereas ProTox-III applies alternative toxicity-oriented predictive models. Therefore, these results should be interpreted as complementary rather than absolute predictions. Thus, the observed differences between ADME and toxicity predictions reflect the distinct nature of pharmacokinetic and toxicological endpoints, where BBB permeability indicates CNS exposure potential, whereas toxicity models evaluate potential adverse biological effects.

#### 2.3.2. Molecular Docking Analysis

In view of the experimentally confirmed preference of the synthesized derivatives for BChE and the growing importance of this enzyme as a therapeutic target in advanced Alzheimer’s disease, docking simulations were conducted in the active-site gorge of human BChE (hBChE) to explore potential binding modes and key ligand–protein interactions. Molecular docking of compounds **3a**–**i** was carried out in the active-site gorge of human BChE. It should be noted that the molecular docking studies were performed using the human BChE crystal structure, whereas the enzymatic inhibition assays employed equine BChE (eqBChE). Although the catalytic gorge is highly conserved between the human and equine enzymes, minor sequence and structural differences may influence ligand binding and should therefore be considered when correlating the docking results with the experimental inhibition data. Accordingly, the docking analysis should be regarded as a structural interpretation of the observed binding interactions rather than a direct quantitative validation of the enzymatic assay results. Additional validation approaches, including molecular dynamics simulations, may further strengthen the interpretation of the predicted binding modes; however, such a large-scale computational study was beyond the scope of the present study. Since the experimentally evaluated compounds correspond to the (R) stereochemical series, only the R-form docking outputs were used for the discussion. For each compound, the selected R pose was analyzed after induced-fit refinement and rescoring. Residue contacts were evaluated from heavy-atom ligand–protein distance matrices and summarized as minimum ligand-residue distances. Donepezil was included as an internal racemic reference. Because the reference compound was used experimentally as a racemate, its R and S configurations were docked separately and are reported as internal reference configurations rather than as individual experimental entities. The two donepezil configurations gave score-function values of −7.15 and −7.10 kcal/mol, respectively (Table 4). These values provide an internal comparison for the docking protocol, but should not be interpreted as quantitative binding free energies. Among the R-form docking outputs, compounds **3f**R, **3g**R, and **3e**R exhibited the most favorable score-function values, with docking scores of −7.07, −6.98, and −6.87 kcal/mol, respectively (Table 4). Their predicted poses were accommodated within the hBChE active-site gorge and positioned in proximity to residues of the catalytic/oxyanion-hole, aromatic/choline-binding, and acyl-binding regions. Compound **3f** displayed the most favorable docking score among the biologically relevant derivatives. Its selected pose involved close contacts with Ser198, Gly117, Trp231, Phe329, Ala199, Trp82, and His438, indicating ligand accommodation across multiple functional regions of the enzyme gorge. The dimethylamino-substituted aromatic moiety (**3f**) may contribute to favorable electrostatic interactions, whereas the terpene-derived scaffold provides hydrophobic complementarity within the binding cavity. Thus, the predicted binding mode of **3f** is compatible with the strong experimental BChE inhibitory activity and reflects key structural features commonly associated with BChE inhibitors, including a nitrogen-containing polar moiety, an aromatic fragment, and an extended hydrophobic region. Compound **3g**R also showed favorable accommodation within the BChE gorge. The closest residues included Gly116, Ser287, Thr120, Leu286, Ser198, Glu197, Val288 and Gly117. This contact pattern indicates that **3g** can occupy the same general binding region as the more active derivatives. However, its experimental BChE inhibition was weaker than that of the leading compounds, indicating that the docking score mainly reflects geometric accommodation within the human BChE model.

**Figure 6:**
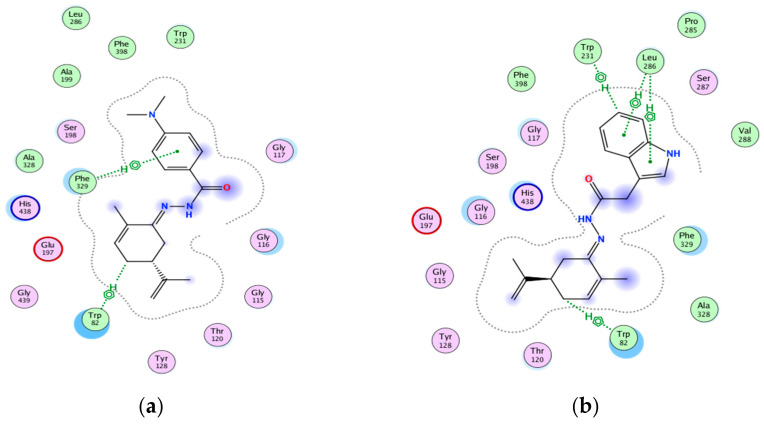
Two-dimensional MOE ligand–receptor interaction map of compounds **3f**R (**a**) and **3g**R (**b**) in the hBChE active-site gorge.

To visualize the predicted binding modes of the highest-scoring and biologically relevant derivatives, two-dimensional MOE interaction maps of compounds **3f** and **3g** are presented in Figure 6. In both maps (Figure 6), the ligand is shown as a 2D chemical structure, and nearby amino-acid residues are shown as labeled circles. Lipophilic residues are colored green, polar residues violet/magenta, acidic residues are outlined in red and basic residues in blue. The grey dotted contour indicates the local pocket boundary, and blue halos denote solvent exposure. Green dashed lines and aromatic symbols denote predicted docking interactions with side-chain polar or aromatic features, whereas blue dashed arrows denote predicted backbone polar contacts. The maps provide a qualitative view of ligand accommodation in the BChE gorge.

The grey dotted contour indicates the local pocket boundary, and blue halos denote solvent exposure. Green dashed lines and aromatic symbols denote predicted docking interactions with side-chain polar or aromatic features, whereas blue dashed arrows denote predicted backbone polar contacts. The maps provide a qualitative view of ligand accommodation in the BChE gorge.

Compound **3e**R ranked next in the R-form docking series and showed contacts with Ser198, Glu197, Gly117, His438, Trp82, Ser287 and Leu286. The remaining derivatives also occupied the gorge, but with less favorable score-function values. Compounds **3d**R, **3a**R, **3h**R, **3c**R and **3i**R retained contacts with functionally relevant gorge residues, including Trp82, Ser198, His438, Gly116/Gly117, Phe329, Tyr332 and Trp231, depending on the ligand orientation. Notably, Trp82 appeared among the recurrent contact residues for most active derivatives, suggesting a potential role of aromatic and hydrophobic interactions in ligand stabilization within the hBChE gorge.

Overall, the R-form docking analysis supports the ability of the synthesized CBD-like hydrazone derivatives to occupy the hBChE active-site gorge. The most favorable R-form pose was obtained for **3f**, in agreement with its strong experimental BChE inhibition. In contrast, the lower docking score of **3b** despite its high experimental potency emphasizes that the docking results should be used as qualitative structural support rather than as a quantitative model of IC_50_ values. Additional hydrogen-inclusive distance-matrix analysis of the protonated complexes did not materially alter the residue-contact patterns identified from the heavy-atom matrices.

### 2.4. In Vitro Studies

#### 2.4.1. Cytotoxicity

A series of MTT assays was performed using human (SH-SY5Y) and murine (Neuro-2a) neuroblastoma cell lines to evaluate the preliminary cytotoxicity and cellular tolerance of compounds **3a**–**i** and CBD. Undifferentiated SH-SY5Y cells were employed as a well-established model for the initial assessment of cytotoxicity rather than for investigating disease-specific neuronal mechanisms. The results obtained from the cell viability assays are presented in Table 5. IC_50_ values were derived from a single concentration–response experiment in which each concentration was tested in technical triplicate, and the corresponding 95% confidence intervals (CI) were obtained from the profile-likelihood method of the nonlinear regression. CBD was used as a reference compound. Cytotoxicity assessment represents an important step in the early in vitro profiling of novel compounds with potential application in Alzheimer’s disease and other neurodegenerative disorders. Since these conditions require long-term treatment, low toxicity toward neuronal cells is a key prerequisite for further development. In this context, the newly synthesized CBD-like hydrazone derivatives were evaluated in human (SH-SY5Y) and murine (Neuro-2a) neuroblastoma cell lines, widely used models for neurotoxicity and neuroprotection studies. All tested compounds **3a**–**i** exhibited higher IC_50_ values than CBD, indicating lower cytotoxicity and better cellular tolerance. This finding is particularly relevant because potential anti-Alzheimer agents should preserve neuronal viability within the concentration range required to exert pharmacological activity. The observed values therefore suggest a sufficiently favorable safety profile for further biological evaluation.

Among the tested derivatives, compound **3b** displayed no significant cytotoxicity toward SH-SY5Y cells up to the highest tested concentration (500 μM), indicating an IC_50_ above the experimental concentration range. The compounds **3a**, **3h**, and **3i** exhibited IC_50_ values above 200 µM, indicating low intrinsic toxicity in the human neuronal model. Such a profile is favorable for neuroprotective applications, as it suggests minimal impairment of neuronal viability at relatively high concentrations. Because the present values were obtained from a single concentration–response experiment, the following structure–activity observations should be regarded as descriptive trends rather than statistically validated differences. The data suggest that the nature and position of aromatic substituents may influence cellular tolerance. Notably, compounds **3a** and **3b**, which differ only in the position of the hydroxyl group (*meta*-OH vs. *para*-OH), showed distinct cytotoxicity profiles. The lower logP value of **3b** (3.80) compared to **3a** (4.80) was associated with lower cytotoxicity toward SH-SY5Y cells (IC_50_ > 500 vs. 239.90 µM), which may indicate that a more favorable lipophilicity/polarity balance decreases nonspecific cellular interactions and improves cellular tolerance. A similar trend was observed for methoxy derivatives, where **3d** and **3e** showed higher cytotoxicity than the corresponding hydroxy analogues. Furthermore, the increased TPSA and hydrogen-bonding capacity of compound **3c** (TPSA = 81.92 Å^2^, HBA/HBD = 4/3) resulting from the additional hydroxyl group were associated with enhanced cytotoxicity (IC_50_ = 68.57 µM). Although increased polarity generally improves aqueous solubility, excessive hydrogen-bonding capacity may promote stronger interactions with intracellular components and alter cellular uptake, contributing to reduced viability. These findings suggest that both electronic effects and lipophilic properties of the aromatic moiety contribute to cellular uptake and intracellular interactions.

Despite the strong electron-donating character of the dimethylamino group, compound **3f** exhibited lower cytotoxicity than the methoxy-substituted analogues **3d** and **3e** (IC_50_ = 160.40 µM), suggesting that electronic effects alone do not determine cellular toxicity. Its favorable cellular tolerance may be attributed to the protonatable dimethylamino moiety, which can modulate polarity under physiological conditions while maintaining an appropriate balance between membrane permeability and cellular tolerance. From an Alzheimer’s disease drug discovery perspective, this substituent is particularly attractive because it introduces a tertiary nitrogen atom, a key pharmacophoric feature of cholinesterase inhibitors such as donepezil. Although structurally simpler than the piperidine ring of donepezil, the dimethylamino group may facilitate ionic and cation–π interactions within the acetylcholinesterase binding gorge. Combined with its favorable cellular tolerance and pronounced inhibition of lipid peroxidation, these characteristics identify **3f** as a promising multifunctional lead for further anti-Alzheimer’s investigations.

Particular attention should be given to the indole-containing derivatives **3g** and **3h**, as the indole scaffold is frequently associated with neuromodulatory and neuroprotective activities. Despite their similar molecular weights and lipophilicity, compound **3g** (MW = 321.42, logP = 4.51, TPSA = 57.25 Å^2^) exhibited considerably higher cytotoxicity (IC_50_ = 70.10 µM) than 3h (MW = 337.42, logP = 4.59, TPSA = 66.48 Å^2^; IC_50_ = 274.80 µM). In addition to its higher polarity and hydrogen-bonding capacity, the lower cytotoxicity of **3h** may be related to its more rigid and conjugated structure, whereas the additional methylene spacer in **3g**, **3h** may be related to its more rigid and conjugated structure, whereas the additional methylene spacer in **3g** increases molecular flexibility and may promote nonspecific cellular interactions. These observations suggest that both physicochemical properties and conformational features of the indole-containing scaffold may influence cellular tolerance, highlighting **3h** as a promising lead for further anti-Alzheimer’s drug development.

Among all synthesized derivatives, compound **3i** exhibited one of the most favorable profiles, combining low cytotoxicity toward SH-SY5Y cells (IC_50_ = 302.10 µM) with the lowest molecular weight (269.34 Da), the lowest lipophilicity (logP = 3.49), and moderate polarity (TPSA = 54.35 Å^2^). This balanced physicochemical profile may contribute to improved cellular tolerance by reducing nonspecific cellular accumulation while maintaining adequate membrane permeability. Consequently, the low cytotoxicity of **3i** represents an attractive feature for further optimization as a potential CNS-active lead.

Interestingly, several compounds exhibited distinct cytotoxicity profiles between the two neuronal cell lines. In particular, **3b** and **3i** were substantially less toxic toward SH-SY5Y cells than Neuro-2a cells, which may reflect differences in cellular metabolism, membrane transport, or protein expression patterns. These observations underscore the importance of employing multiple cellular models during the early stages of biological evaluation. Overall, the results demonstrate that most synthesized hydrazone derivatives possess a favorable profile of low cytotoxicity. In contrast, CBD exhibited the highest cytotoxicity toward both neuronal cell lines (IC_50_ = 15.40–17.52 µM). This behavior may be partially attributed to its pronounced lipophilicity (logP = 5.85) and low polarity (TPSA = 40.46 Å^2^), which distinguish it from the synthesized derivatives. These properties may facilitate enhanced cellular uptake and membrane association, potentially contributing to the stronger cytotoxic effects observed in vitro.

Notably, compounds **3a**, **3b**, **3f**, **3h**, and **3i** combined low cytotoxicity with structural features commonly associated with CNS-active molecules. Together with their antioxidant activity observed in chemical assays, these findings support further evaluation of their protective effects against H_2_O_2_-induced oxidative stress in neuronal cell models. Such studies are currently in progress and are expected to provide additional insight into their neuroprotective potential.

#### 2.4.2. Cholinesterase Inhibition

The AChE and BChE inhibitory activity results of the novel CBD-like hydrazone derivatives are provided in Table 6. To assess their dual inhibitory profile, the improved Ellman spectrophotometric method [60] was employed, with certain modifications [61]. Donepezil was used as a reference compound. Results are presented with IC_50_ values as the mean of three independent measurements ± standard deviation. Enzyme inhibition percentages at 500 μM and selectivity indices for the compounds are also added (Table 6).

All newly synthesized compounds exhibited a clear and consistent preference for butyrylcholinesterase (BChE) inhibition over acetylcholinesterase (AChE), indicating a pronounced BChE-directed pharmacological profile. This selectivity was evidenced by both enzyme inhibitory potency and selectivity indices, with most derivatives showing weak or negligible AChE inhibition (IC_50_ > 1000 μM) in contrast to stronger activity against BChE (IC_50_ values ranging from 1.67 to 544.50 μM), with the most active derivatives exhibiting inhibition at the low micromolar level. As a result, high selectivity indices were obtained across the series, identifying compounds **3b** (SI = 515.46) and **3f** (SI = 189.34) as the most potent and highly BChE-selective inhibitors. Among the evaluated derivatives, compounds **3b** and **3f** emerged as the most promising candidates, combining strong BChE inhibition (IC_50_ = 1.94 ± 0.30 μM and 1.67 ± 0.11 μM, respectively) with minimal AChE activity. A second group of compounds, including **3a**, **3d**, **3i**, **3c**, and **3e**, demonstrated moderate-to-strong selectivity (SI ≈ 10–34), primarily driven by reduced AChE inhibition rather than exceptional BChE potency. In contrast, compounds **3g** and **3h** displayed weak BChE selectivity (SI < 2), indicating a lack of significant differentiation in inhibitory activity toward the two enzymes. It should be noted that the calculated selectivity indices are based on enzyme preparations from different sources and should therefore be considered as comparative indicators of relative enzyme preference rather than absolute measures of selectivity. Further studies using human cholinesterase isoforms are required to confirm the selectivity profile of the most promising compounds.

The observed biological profile aligns with the increasingly recognized role of BChE as a disease-relevant target in Alzheimer’s pathology. While AChE remains the primary enzyme responsible for acetylcholine hydrolysis under physiological conditions, its activity may decrease during disease progression, whereas BChE expression and functional contribution are relatively preserved or increased, particularly in advanced stages of AD [62]. Consequently, BChE-selective inhibitors have gained increasing interest as potential therapeutic agents due to their ability to target the altered cholinergic landscape associated with neurodegeneration. Recent studies suggest that BChE-selective ligands may provide pharmacological advantages by sustaining cholinergic signaling under pathological conditions in which BChE becomes a more relevant functional hydrolase [63,64]. Therefore, selective BChE modulation may represent a complementary strategy to classical AChE-centered approaches, particularly for addressing cholinergic dysfunction in later stages of AD [63,64]. In this context, the strong BChE-selective profile observed for the present series highlights their potential as lead structures for further investigation targeting cholinergic dysfunction associated with disease progression [62,64].

Thus, compounds **3b** and **3f** emerged as the most promising lead candidates for the development of multitarget agents for late-stage Alzheimer’s disease. These compounds combine fairly low cytotoxicity in human neuroblastoma cells with potent and selective BChE inhibition, and sustained protection against lipid peroxidation. Such a pharmacological profile is particularly relevant in advanced stages of Alzheimer’s disease, where BChE activity progressively increases while AChE activity declines. Notably, both compounds exhibited minimal AChE inhibition, indicating a clear preference for BChE as the primary cholinergic target. Overall, the results reveal a consistent structure–activity relationship favoring BChE-selective inhibition, thereby distinguishing this series from classical AChE-directed cholinesterase inhibitors and highlighting the potential of these scaffolds for further optimization as multifunctional anti-Alzheimer agents.

#### 2.4.3. Antioxidant Activity

Oxidative stress is widely recognized as a key contributor to the pathogenesis and progression of Alzheimer’s disease (AD), where excessive generation of reactive oxygen species (ROS) leads to lipid peroxidation, mitochondrial dysfunction, synaptic failure, and ultimately neuronal death. Importantly, oxidative damage is tightly interconnected with amyloid-β (Aβ) aggregation and tau hyperphosphorylation, creating a self-propagating cycle of neurodegeneration. As emphasized in the recent literature, oxidative stress is both a cause and consequence of Aβ pathology, amplifying neuronal injury and synaptic dysfunction [65,66,67]. Therefore, the development of multifunctional antioxidants capable of interrupting ROS-driven cascades is considered a promising therapeutic strategy in AD research. In the present study, the antioxidant properties of the synthesized hydrazone derivatives were evaluated using complementary in vitro assays (DPPH, ABTS, FRAP, and FTC), each reflecting distinct mechanisms of antioxidant action, including hydrogen atom transfer (HAT), single electron transfer (SET), and inhibition of lipid peroxidation in membrane-mimicking systems. Results are presented in Table 7 and Table 8.

The antioxidant properties of the synthesized derivatives were assessed using complementary assays based on different reaction mechanisms, including DPPH and ABTS radical scavenging assays, ferric-reducing antioxidant power (FRAP), and lipid peroxidation inhibition (FTC). Overall, the compounds exhibited moderate and assay-dependent antioxidant activity, with substantial differences depending on the underlying redox mechanism. As expected, none of the tested derivatives exceeded the activity of ascorbic acid in classical radical-scavenging assays, highlighting that their antioxidant effects are weaker than those of established reference antioxidants. However, several derivatives demonstrated favourable activity in specific assays, suggesting their potential contribution to multitarget neuroprotective profiles.

In the DPPH assay, compound **3b** exhibited the highest radical-scavenging activity among the tested derivatives (IC_50_ = 8.52 mM), followed by compounds **3e** (IC_50_ = 13.13 mM) and **3f** (IC_50_ = 27.22 mM). Nevertheless, these activities remained considerably lower than that of ascorbic acid (IC_50_ = 0.28 mM), indicating limited hydrogen atom transfer efficiency compared with the reference antioxidant. The improved performance of **3b** may be associated with the presence of a *para*-hydroxyl substituent, which facilitates hydrogen donation and stabilization of the resulting phenoxyl radical through resonance effects. This observation is consistent with the well-established contribution of phenolic hydroxyl groups to radical-quenching mechanisms [68]. Interestingly, the presence of additional hydroxyl groups did not necessarily enhance DPPH activity. Compound **3c**, despite containing two hydroxyl substituents, showed no measurable activity in this assay. This behaviour may be related to structural factors, such as intramolecular hydrogen bonding involving the *ortho*-hydroxyl group, which can reduce proton accessibility and decrease the efficiency of hydrogen donation. Similar effects have been reported for sterically constrained phenolic antioxidants [69].

The ABTS assay revealed a different activity pattern, with **3e** (IC_50_ = 0.22 mM) and **3b** (IC_50_ = 0.37 mM) representing the most active derivatives. Although both compounds remained less potent than ascorbic acid (IC_50_ = 0.11 mM) and CBD (IC_50_ = 0.10 mM), their substantially improved performance compared with the remaining derivatives suggests more favourable electron-transfer capacity. The higher ABTS activity of **3e** may be attributed to the presence of an electron-donating methoxy substituent, which increases electron density within the aromatic system and facilitates reduction of the ABTS•^+^ radical. These findings indicate that methoxy substitution may enhance redox activity through electron-transfer mechanisms rather than solely hydrogen atom donation. Compound **3f**, containing a dimethylamino substituent, also displayed measurable ABTS scavenging activity (IC_50_ = 5.35 mM). The electron-donating properties of this group may contribute to increased aromatic electron density and improved radical stabilization. However, the weaker activity compared with **3b** and **3e** suggests that electronic effects alone are insufficient to determine antioxidant performance, and that steric and structural factors also influence activity.

The FRAP assay further demonstrated that antioxidant behaviour was highly dependent on the assay conditions. Compound **3h** showed the highest ferric-reducing capacity (51.47 ± 3.46 mM TE/mM), followed by **3e** (34.76 ± 3.94 mM TE/mM), **3b** (26.25 ± 0.57 mM TE/mM), and **3g** (25.24 ± 3.44 mM TE/mM). The increased reducing ability of indole-containing derivatives may be associated with enhanced π-electron delocalization, which facilitates electron donation. However, the FRAP results did not directly correlate with DPPH or ABTS activity, confirming that different antioxidant assays reflect distinct chemical mechanisms.

From a medicinal chemistry perspective, the antioxidant profile of the investigated derivatives suggests that specific structural motifs contribute differently to redox modulation. Phenolic hydroxyl groups appear to favour radical neutralization through hydrogen-transfer mechanisms, whereas electron-rich substituents and indole scaffolds may enhance electron-transfer-based reducing activity. Importantly, while the synthesized compounds do not surpass classical antioxidants such as ascorbic acid in standard radical-scavenging assays, several derivatives, particularly **3b** and **3e**, display favourable assay-dependent antioxidant properties. Oxidative damage to neuronal membranes is considered a critical component of Alzheimer’s disease (AD) pathology. Due to their high content of polyunsaturated fatty acids, neuronal membranes are particularly vulnerable to oxidative degradation, resulting in the formation of reactive aldehydes, including malondialdehyde and 4-hydroxynonenal, which have been associated with amyloid pathology, tau dysregulation, mitochondrial impairment, and synaptic dysfunction. Therefore, inhibition of lipid peroxidation represents a biologically relevant parameter for evaluating the neuroprotective antioxidant potential of multifunctional AD-oriented compounds. In the present study, the ability of the synthesized derivatives to inhibit lipid peroxidation was evaluated using the ferric thiocyanate (FTC) method in a linoleic acid emulsion system (Table 8).

During lipid oxidation, generated peroxides oxidize Fe^2+^ to Fe^3+^, which subsequently forms a ferric thiocyanate complex with a characteristic absorption maximum at 500 nm. Therefore, increased absorbance reflects higher peroxide formation, whereas compounds capable of delaying oxidative propagation decrease peroxide accumulation and consequently reduce absorbance values. The FTC assay revealed distinct differences in the ability of the tested derivatives to prevent lipid oxidation. Among the synthesized compounds, **3f** and **3b** demonstrated the most pronounced inhibitory effects, maintaining relatively stable absorbance values throughout the incubation period. These derivatives showed improved protection compared with CBD, suggesting enhanced capacity to interfere with lipid peroxidation processes under the applied experimental conditions. In contrast, several derivatives displayed weaker or time-dependent protection, as indicated by increasing absorbance values during incubation. Compound **3f** exhibited the most consistent inhibition profile, with absorbance values remaining low throughout the assay period. This behaviour suggests effective suppression of oxidative propagation reactions within the lipid environment rather than simple radical scavenging activity. Similarly, compound **3b** demonstrated sustained protection against lipid oxidation, supporting its favourable multitarget profile. Importantly, the FTC performance of these compounds does not directly correlate with their DPPH or ABTS activities, emphasizing that classical radical-scavenging assays do not fully predict membrane-level antioxidant behaviour. Notably, compound **3c**, despite showing limited activity in the DPPH assay, displayed measurable inhibition of lipid peroxidation. This observation further supports the concept that antioxidant activity is highly dependent on the experimental model and that protection against lipid oxidation may involve mechanisms beyond hydrogen atom transfer, including stabilization of lipid radicals or modulation of oxidative chain reactions. From a structure–activity perspective, the results suggest that different structural features contribute to distinct antioxidant mechanisms. The phenolic hydroxyl group present in **3b** may facilitate hydrogen donation and stabilization of radical intermediates, whereas the dimethylamino substituent in **3f** may enhance electron density and contribute to redox modulation. Although **3e** showed favourable performance in ABTS and FRAP assays, its weaker lipid peroxidation inhibition indicates that high electron-transfer capacity does not necessarily translate into efficient protection against lipid-phase oxidation. Similarly, compound **3h**, which demonstrated the highest reducing capacity in the FRAP assay, showed limited protection in the FTC model. This difference highlights the importance of considering assay-specific mechanisms when interpreting antioxidant activity. The strong reducing ability of 3 h may be related to the electron-rich indole scaffold, which facilitates electron donation and stabilization of oxidized intermediates. However, efficient ferric ion reduction does not necessarily correspond to effective inhibition of lipid radical propagation.

Overall, the antioxidant evaluation indicates that the synthesized derivatives possess moderate and mechanism-dependent antioxidant properties, with different compounds exhibiting advantages in specific assays. Compounds **3b**, **3e**, **3f**, and **3h** showed the most notable antioxidant-related characteristics; however, their activity profiles differed substantially. Compound **3e** displayed the most favourable ABTS activity and reducing capacity, while **3h** was the strongest FRAP-active derivative. In contrast, **3b** and **3f** demonstrated the most relevant protection against lipid peroxidation, a parameter more closely associated with oxidative membrane damage in AD. Considering the multifactorial nature of AD, compounds capable of simultaneously modulating cholinergic dysfunction and reducing oxidative membrane damage may represent valuable starting points for further optimization. Based on the integrated antioxidant profile and other pharmacological properties, **3b** and **3f** emerge as promising multifunctional lead structures for further investigation as potential anti-Alzheimer agents. Although the antioxidant effects were observed at higher concentrations than those required for BChE inhibition, this difference should be considered when assessing the multitarget profile of the synthesized derivatives. In vitro antioxidant assays are widely used for preliminary evaluation; however, their results should be interpreted with caution due to several conceptual and methodological limitations. The radical-scavenging and reducing activities measured under experimental conditions may not fully reflect the antioxidant behavior in vivo, where factors such as absorption, metabolism, bioavailability, and interactions with biological systems can significantly influence the overall response. Moreover, these assays often employ synthetic radicals and non-physiological conditions, limiting the direct extrapolation of the obtained findings to biological environments. Therefore, the antioxidant results reported herein should be regarded as comparative indicators of antioxidant capacity rather than direct predictors of in vivo efficacy. To achieve a more comprehensive evaluation, the synthesized compounds were assessed using four complementary assays based on different mechanisms, including radical scavenging, reducing capacity, and inhibition of lipid peroxidation in both aqueous and lipid phases. Further pharmacokinetic and in vivo studies are planned to determine the contribution of antioxidant activity to the overall biological effects of the most promising derivatives under physiologically relevant conditions.

### 2.5. Multitarget Profile Analysis and Preliminary Structure–Activity Relationship (SAR) Considerations

The preliminary SAR analysis, supported by the molecular docking results, suggests that the BChE inhibitory activity and selectivity of the synthesized derivatives are influenced by the interplay between substituent properties, hydrogen-bonding capacity, aromatic interactions, and overall molecular balance.

Compound **3f**, which displayed the highest BChE inhibitory activity, contains a dimethylamino-substituted aromatic moiety that may contribute to ligand recognition and stabilization within the BChE active-site gorge. The predicted binding mode of **3f** indicated favorable accommodation across multiple functional regions of the enzyme, with contacts involving Ser198, Gly117, Trp231, Phe329, Ala199, Trp82, and His438. The presence of a basic nitrogen-containing group may facilitate polar and/or electrostatic interactions, whereas the aromatic fragment and terpene-derived scaffold provide complementary hydrophobic interactions within the binding cavity. These structural features are consistent with characteristics commonly observed in cholinesterase inhibitors, where the combination of a basic moiety and hydrophobic aromatic regions contributes to enhanced enzyme affinity.

In contrast, compound **3b** exhibited the most balanced pharmacological profile, combining high BChE selectivity with pronounced antioxidant activity and excellent cellular tolerability. Although its docking score was not among the most favorable within the series, its strong experimental activity emphasizes that docking ranking alone cannot fully predict inhibitory potency. The hydroxyl substituent in **3b** may contribute to its multifunctional profile by providing additional hydrogen-bonding capacity and enhancing antioxidant properties through proton-donating ability. Thus, the activity of **3b** may reflect a favorable balance between enzyme interaction, antioxidant potential, and cellular compatibility rather than maximal predicted binding affinity.

The comparison with the remaining derivatives further highlights the importance of substituent optimization. Compounds **3a**, **3d**, **3e**, and **3i** showed moderate BChE inhibition and selectivity, suggesting that differences in electronic properties, steric effects, and molecular polarity may influence their accommodation within the enzyme gorge. Although compound **3i** demonstrated favorable cellular tolerability, its lower BChE selectivity indicates that improved safety alone is insufficient to achieve an optimal multitarget profile. Compound **3c**, containing an additional dihydroxy-substituted aromatic moiety, exhibited enhanced antioxidant activity; however, this modification did not result in improved cholinesterase selectivity. This observation suggests that increased polarity and hydrogen-bonding capacity may favor radical-scavenging properties while potentially affecting hydrophobic interactions required for efficient enzyme binding.

Similarly, the indole-containing derivatives **3g** and **3h** did not demonstrate superior BChE inhibition despite the presence of an additional aromatic heterocycle. Although docking analysis showed that **3g** could be accommodated within the hBChE gorge and interact with functionally relevant residues, including Gly116, Ser287, Thr120, Leu286, Ser198, and Gly117, the weaker experimental inhibition indicates that favorable geometric accommodation alone is insufficient to ensure enhanced inhibitory activity.

Overall, the preliminary SAR trends indicate that BChE selectivity is not governed by a single structural determinant but rather by the combined effects of basic functionality, aromatic/hydrophobic interactions, hydrogen-bonding capacity, and physicochemical balance. Within this series, the hydroxyl-containing derivative **3b** and the dimethylamino-substituted derivative **3f** represent complementary examples of favorable structural optimization, contributing to selective BChE inhibition, antioxidant activity, and cellular tolerability through distinct structural features. However, these SAR observations remain preliminary due to the limited chemical diversity of the current library, and further analogue development will be required to validate and refine the identified structure–activity relationships.

## 3. Materials and Methods

### 3.1. Materials

Chemical reagents: Commercial reagents and solvents were purchased from standard suppliers and used without further purification. Ethanol, methanol, dimethyl sulfoxide (DMSO), chloroform, and other synthetic reagents were obtained from Sigma-Aldrich (Merck KGaA, Darmstadt, Germany) and TCI Chemicals (Tokyo, Japan). Cannabidiol (CBD) used in this study was a synthetic product obtained by stereoselective synthesis, providing a structural and stereochemical configuration identical to the naturally occurring (–)(1R,6R)-CBD. The enantiomeric purity and chemical composition were confirmed by chiral HPLC (≥98% ee; ≥99% chemical purity). CBD (catalog number LPM THC 303 Cannabidiol) was manufactured by LGC Standards/Lipomed (Switzerland) and supplied through Bio Connect. The compound was handled and stored according to the manufacturer’s recommendations to maintain stability and enantiomeric purity. (R)-(+)-Carvone was purchased from Sigma-Aldrich (Merck KGaA, Darmstadt, Germany) as an analytical-grade reagent and used without further purification. The compound corresponded to the naturally occurring (R)-(+)-carvone enantiomer with a purity of ≥98% according to the manufacturer’s certificate of analysis. Storage and handling were performed according to the supplier’s instructions. All antioxidant assay reagents, including 2,2′-diphenyl-1-picrylhydrazyl (DPPH), 2,2′-azinobis-(3-ethylbenzothiazoline-6-sulfonic acid) (ABTS), Trolox, 2,4,6-tripyridyl-s-triazine (TPTZ), ascorbic acid, potassium persulfate, linoleic acid, ammonium thiocyanate, and iron(II) chloride (FeCl_2_), were obtained from Sigma-Aldrich (Merck KGaA, Darmstadt, Germany). All other chemicals and solvents were of analytical grade. MTT reagent [3-(4,5-dimethylthiazol-2-yl)-2,5-diphenyltetrazolium bromide] was purchased from Sigma-Aldrich (Merck KGaA, Darmstadt, Germany). L-glutamine, phosphate-buffered saline (PBS), and dimethyl sulfoxide (DMSO) was purchased from Sigma-Aldrich (Merck KGaA, Darmstadt, Germany).

Biological reagents: Acetylcholinesterase (AChE) from Electrophorus electricus, butyrylcholinesterase (BChE) from equine serum, 5,5′-dithiobis(2-nitrobenzoic acid) (DTNB), acetylthiocholine iodide (ATCI), and butyrylthiocholine iodide (BTCI) were purchased from Sigma-Aldrich (Merck KGaA, Darmstadt, Germany). Enzyme inhibition assays were performed using an ELISA microplate reader (EL10A, BIOBASE, Jinan, China). Human neuroblastoma SH-SY5Y cells were obtained from the European Collection of Authenticated Cell Cultures (ECACC; supplied through Sigma-Aldrich, Merck KGaA, Darmstadt, Germany), while murine Neuro-2a neuroblastoma cells were purchased from the American Type Culture Collection (ATCC, Manassas, VA, USA). RPMI-1640 culture medium and fetal bovine serum (FBS) were obtained from Sigma-Aldrich (Merck KGaA, Darmstadt, Germany).

### 3.2. Chemistry

#### 3.2.1. General

All chemicals, reagents, and solvents were commercially available and used without further purification. The progress of the reactions was monitored by thin-layer chromatography (TLC). Melting points were determined in open glass capillaries using a Büchi Melting Point M-565 apparatus (BUCHI Labortechnik AG, Flawil, Switzerland) and are reported uncorrected. NMR spectra were recorded in DMSO-*d*_6_ on a Bruker Avance III 600 MHz spectrometer using tetramethylsilane (TMS) as an internal reference. Chemical shifts (δ) are expressed in parts per million (ppm). High-resolution mass spectra (HRMS) were obtained using an Agilent Accurate-Mass Q-TOF LC/MS G6520B system equipped with a dual electrospray ionization (ESI) source (Agilent Technologies, Santa Clara, CA, USA).

#### 3.2.2. General Procedure for the Synthesis of Compounds **3a**–**i**

(−)-Carvone (**1**) (0.002 mol) was treated with the appropriate hydrazide derivative (**2a**–**i**) (0.002 mol) in ethanol (10 mL/mmol of ketone) in the presence of catalytic *p*-toluenesulfonic acid (*p*-TSA, 1 mol%). The reaction mixture was stirred at 60 °C for 8–12 h, and the reaction progress was monitored by thin-layer chromatography (TLC) on silica gel using petroleum ether/ethyl acetate (8:2 to 6:4, *v*/*v*) as the mobile phase. After completion of the reaction, the solvent was reduced under pressure, and the crude products were purified by recrystallization from ethanol to obtain the target compounds (**3a**–**i**) in good yields. All synthesized compounds were characterized by NMR spectroscopy and HRMS. The detailed NMR spectra and HRMS, are provided in the Supplementary Materials (Figures S1–S62).

#### 3.2.3. Structural Characterization

*(R,E)-3-Hydroxy-N’-(2-methyl-5-(prop-1-en-2-yl)cyclohex-2-en-1-ylidene)benzohydrazide* (**3a**) White solid; yield: 71%; m.p. 223–225 °C; ^1^H NMR (600 MHz, DMSO-*d*_6_): δ = 1.74 (s, 3H, cyclo-CH_3_), 1.86 (s, 3H, cyclo-CH_3_), 2.11 (t, J = 13.9 Hz, 1H, H-4), 2.19 (t, J = 14.3 Hz, 1H, H-6), 2.26 (dt, J = 4.3, 17.8 Hz, 1H, H-4), 2.35 (t, J = 11.5 Hz, 1H, H-5), 2.92 (d, J = 14.8 Hz, 1H, H-6), 4.78 (s, 1H, =CH_2_), 4.81 (s, 1H, =CH_2_), 6.22 (s, 1H, H-3), 6.93 (d, J = 6.0 Hz, 1H, H-4′), 7.19 (s, 1H, H-2′), 7.23 (d, d, J = 6.8 Hz, 1H, H-6′), 7.27 (t, J = 6.4 Hz, 1H, H-5′), 9.72 (bs, 1H, OH), 10.55 (bs, 1H, NH). ^13^C NMR (151 MHz, DMSO-*d*_6_): δ = 17.95 (cyclo-CH_3_), 20.54 (CH_3_), 29.79 (C-4), 30.07 (bs, C-6), 40.37 (C-5), 110.22 (=CH_2_), 114.58 (C-2′), 118.32 (bs, C-4′ and C-6′), 129.42 (C-5′), 132.45 (C-2), 134.27 (C-3), 135.66 (C-1′), 147.70 (C=), 157.24 (C=N), 157.42 (C-3′), 163.80 (C=O). HRMS (ESI) *m*/*z* calcd for C_17_H_21_N_2_O_2_ [M + H]^+^ 285.15975; found 285.15914.

*(R,E)-4-Hydroxy-N’-(2-methyl-5-(prop-1-en-2-yl)cyclohex-2-en-1-ylidene)benzohydrazide* (**3b**). White solid; yield: 78%; m.p. 223–225 °C. The ^1^H and ^13^C NMR spectra of compound 3b were previously reported in the literature [51] (https://doi.org/10.1080/17568919.2025.2515821); herein, a detailed assignment recorded at 600 MHz is presented. ^1^H NMR (600 MHz, DMSO-*d*_6_) δ 1.74 (s, 3H, CH_3_), 1.84 (s, 3H, cyclo-CH_3_), 2.11 (dd, J = 10.7, 17.0 Hz, 1H, H-4), 2.18 (d, J = 13.6 Hz, 1H, H-6), 2.25 (td, J = 4.9, 17.3 Hz, 1H, H-4), 2.32–2.37 (m, 1H, H-5), 2.93 (d, J = 15.1 Hz, 1H, H-6), 4.78 (s, 1H, =CH_2_), 4.81 (s, 1H, =CH_2_), 6.18 (s, 1H, H-3), 6.81 (d, J = 8.4 Hz, 2H, Ar-H), 7.72 (d, J = 8.5 Hz, 2H, Ar-H), 10.01 (br s, 1H, OH), 10.39 (s, 1H, NH). ^13^C NMR (151 MHz, DMSO-*d*_6_): δ = 17.94 (cyclo-CH_3_), 20.53 (CH_3_), 29.73 (C-4), 29.87 (bs, C-6), 40.34 (C-5), 110.13 (=CH_2_), 114.73 (C-3′ and C-5′), 124.62 (C-1′), 129.78 (C-2′ and C-6′), 132.49 (C-2), 133.55 (C-3), 147.67 (C=), 156.4 (C=N), 160.30 (C-4′), 163.3 (C=O). HRMS (ESI) *m*/*z* calcd for C_17_H_21_N_2_O_2_ [M + H]^+^ 285.15975; found 285.15917.

*(R,E)-2,4-Dihydroxy-N’-(2-methyl-5-(prop-1-en-2-yl)cyclohex-2-en-1-ylidene)benzohydrazide* (**3c**). White solid; yield: 82%; m.p. 220 °C. ^1^H NMR (600 MHz, DMSO-*d*_6_) δ 1.76 (s, 3H, CH_3_), 1.87 (s, 3H, cyclo-CH_3_), 2.13 (tdd, J = 2.4, 11.4, 17.1 Hz, 1H, H-4), 2.21 (dd, J = 12.7, 15.8 Hz, 1H, H-6), 2.26 (td, J = 5.1, 17.5 Hz, 1H, H-4), 2.39–2.44 (m, 1H, H-5), 2.74 (d, J = 14.3 Hz, 1H, H-6), 4.80 (m, 1H, =CH_2_), 4.82 (d, J = 0.5 Hz, 1H, =CH_2_), 6.19 (d, J = 5.1 Hz, 1H, H-3), 6.36 (dd, J = 2.2, 7.8 Hz, 1H, H-5′), 6.38 (br s, 1H, H-3′), 7.80 (d, J = 7.8 Hz, 1H, H-6′), 10.06 (s, 1H, NH), 10.99 (s, 1H, OH), 11.75 (s, 1H, OH). ^13^C NMR (151 MHz, DMSO-*d*_6_): δ = 17.88 (cyclo-CH_3_), 20.48 (CH_3_), 29.48 (C-4), 29.56 (C-6), 40.15 (C-5), 102.63 (C-3′), 107.87 (C-5′), 109.08 (C-1′), 110.31 (=CH_2_), 131.89 (C-6′), 132.38 (C-2), 133.42 (C-3), 147.61 (C=), 153.34 (C=N), 158.55 (C-2′), 161.93 (C-4′), 162.33 (C=O). HRMS (ESI) *m*/*z* calcd for C_17_H_21_N_2_O_3_ [M + H]^+^ 301.15467; found 301.15401.

*(R,E)-4-Methoxy-N’-(2-methyl-5-(prop-1-en-2-yl)cyclohex-2-en-1-ylidene)benzohydrazide* (**3d**). White solid, yield: 85%;. The ^1^H and ^13^C NMR spectra of compound **3d** were previously reported [51] (https://doi.org/10.1080/17568919.2025.2515821); herein, the detailed spectral assignment at 600 MHz is provided. ^1^H NMR (600 MHz, DMSO-*d*_6_) δ 1.75 (s, 3H, CH_3_), 1.85 (br s, 3H, cyclo-CH_3_), 2.08–2.14 (m, 1H, H-4), 2.18–2.20 (m, 1H, H-6), 2.26 (td, J = 5.1, 17.4 Hz, 1H, H-4), 2.35 (m, 1H, H-5), 2.94 (d, J = 15.5 Hz, 1H, H-6), 3.82 (s, 3H, OCH_3_), 4.78 (t, J = 1.5 Hz, 1H, =CH_2_), 4.81 (t, J = 0.8 Hz, 1H, =CH_2_), 6.20 (br s, 1H, H-3), 7.01 (d, J = 8.3 Hz, 2H, Ar-H), 7.82 (d, J = 8.7 Hz, 2H, Ar-H), 10.50 (s, 1H, NH). ^13^C NMR (151 MHz, DMSO-*d*_6_) δ = 17.94 (cyclo-CH_3_), 20.55 (CH_3_), 29.74 (C-4), 29.95 (C-6), 40.31 (C-5), 55.37 (OCH_3_), 110.14 (=CH_2_), 113.45 (C-3′ and C-5′), 126.26 (C-1′), 129.67 (C-2′ and C-6′), 132.44 (C-2), 133.89 (C-3), 147.67 (C=), 157.00 (C=N), 161.66 (C-4′), 163.00 (C=O). HRMS (ESI) *m*/*z* calcd for C_18_H_23_N_2_O_2_ [M + H]^+^ 299.17540; found 299.17483.

*(R,E)-2-Methoxy-N’-(2-methyl-5-(prop-1-en-2-yl)cyclohex-2-en-1-ylidene)benzohydrazide* (**3e**). White solid; yield: 79%; m.p. 115–116 °C. Compound **3e** existed as a 1:0.16 mixture of synperiplanar and antiperiplanar conformers around the amide bond in DMSO-*d*_6_. ^1^H NMR (600 MHz, DMSO-*d*_6_) δ 1.77 (s, 3H, CH_3_), 1.88 (s, 3H, cyclo-CH_3_), 2.11 (tdd, J = 2.5, 11.4, 17.4 Hz, 1H, H-6), 2.24 (dd, J = 12.6, 16.0 Hz, 1H, H-4), 2.29 (td, J = 5.1, 17.5 Hz, 1H, H-6), 2.38–2.43 (m, 1H, H-5), 2.80 (dd, J = 3.9, 15.8 Hz, 1H, H-4), 3.93 (s, 3H, OCH_3_), 4.82 (s, 1H, =CH_2_), 4.83 (s, 1H, =CH_2_), 6.22 (d, J = 5.2 Hz, 1H, H-3), 7.10 (dt, J = 0.8, 7.5 Hz, 1H, H-5′), 7.20 (d, J = 8.2 Hz, 1H, H-3′), 7.53 (ddd, J = 1.7, 8.4, 7.3 Hz, 1H, H-4′), 7.83 (dd, J = 1.7, 7.6 Hz, 1H, H-6′), 10.78 (s, 1H, NH); resolved signals for minor *antiperiplanar* conformer around the amide bond: 1.34 (s, 3H, cyclo-CH_3_), 1.74 (s, 3H, CH_3_), 3.71 (s, 3H, OCH_3_), 4.78 (s, 1H, =CH_2_), 4.80 (s, 1H, =CH_2_), 10.75 (s, 1H, NH). ^13^C NMR (151 MHz, DMSO-*d*_6_) δ 17.83 (cyclo-CH_3_), 20.61 (CH_3_), 29.43 (C-4), 29.54 (C-6), 40.05 (C-5), 56.24 (OCH_3_), 110.05 (=CH_2_), 112.24 (C-3′), 120.91 (C-5′), 122.16 (C-1′), 130.68 (C-6′), 132.36 (C-4′), 132.69 (C-2), 133.86 (C-3), 147.64 (C=), 154.08 (C=N), 156.90 (C-2′), 161.17 (C=O). HRMS (ESI) *m*/*z* calcd for C_18_H_23_N_2_O_2_ [M + H]^+^ 299.17540; found 299.17483.

*(R,E)-4-(Dimethylamino)-N’-(2-methyl-5-(prop-1-en-2-yl)cyclohex-2-en-1-ylidene)benzohydrazide* (**3f**). White solid; yield: 86%; m.p. 167–169 °C. ^1^H NMR (600 MHz, DMSO-*d*_6_) δ 1.75 (s, 3H, CH_3_), 1.84 (s, 3H, cyclo-CH_3_), 2.11 (tdd, J = 2.4, 11.2, 17.2 Hz, 1H, H-4), 2.17 (t, J = 14.3 Hz, 1H, H-6), 2.25 (td, J = 5.3, 17.4 Hz, 1H, H-4), 2.32–2.38 (m, 1H, H-5), 2.93 (dd, J = 3.3, 16.2 Hz, 1H, H-6), 2.98 (s, 6H, N(CH_3_) _2_), 4.78 (t, J = 1.5 Hz, 1H, =CH_2_), 4.81 (t, J = 0.8 Hz, 1H, =CH_22_), 6.17 (d, J = 4.8 Hz, 1H, H-3), 6.72 (d, J = 8.9 Hz, 2H, Ar-H), 7.75 (d, J = 8.4 Hz, 2H, Ar-H), 10.27 (s, 1H, NH). ^13^C NMR (151 MHz, DMSO-*d*_6_) δ = 17.99 (cyclo-CH_3_), 20.57 (CH_3_), 29.72 (C-4 and C-6), 40.32 (C-5), 110.09 (=CH_2_), 110.66 (C-3′ and C-5′), 120.36 (C-1′), 129.54 (C-2′ and C-6′), 132.54 (C-2), 133.19 (C-3), 147.74 (C=), 152.26 (C=N), 155.76 (C-4′), 163.61 (C=O). HRMS (ESI) *m*/*z* calcd for C_19_H_26_N_3_O [M + H]^+^ 312.20704; found 312.20637.

*(R,E)-2-(1H-Indol-3-yl)-N’-(2-methyl-5-(prop-1-en-2-yl)cyclohex-2-en-1-ylidene)acetohydrazide* (**3g**). White solid; yield: 75%; m.p. 163.9 °C. Compound **3g** existed as a 1:0.64 mixture of *synperiplanar* and *antiperiplanar* conformers around the amide bond in DMSO-*d*_6_. ^1^H NMR (600 MHz, DMSO-*d*_6_) δ 1.73 (s, 3H, CH_3_), 1.89 (s, 3H, cyclo-CH_3_), 2.00 (dd, J = 12.4, 16.6 Hz, 1H, H-6), 2.03–2.12 (m, 1H, H-4), 2.10–2.35 (m, 2H, H-5 and H-4), 2.91 (dd, J = 3.6, 16.6 Hz, 1H, H-6), 4.00 (s, 2H, CH_2_), 4.79 (s, 2H, =CH_2_), 6.14 (d, J = 3.7 Hz, 1H, H-3), 6.96 (t, J = 7.5 Hz, 1H, H-5′), 7.05 (t, J = 7.0 Hz, 1H, H-6′), 7.17 (d, J = 2.2 Hz, 1H, H-2′), 7.34 (d, J = 8.1 Hz, 1H, H-7′), 7.53 (d, J = 7.9 Hz, 1H, H-4′), 10.37 (s, 1H, CONH), 10.84 (s, 1H, NH). Signals corresponding to the minor *antiperiplanar* conformer around the amide bond were observed at δ 1.74 (s, 3H, CH_3_), 1.78 (s, 3H, cyclo-CH_3_), 2.06 (dd, J = 13.1, 16.3 Hz, 1H, H-6), 2.84 (dd, J = 3.6, 16.2 Hz, 1H, H-6), 3.70 (s, 2H, CH_2_), 4.76 (s, 2H, =CH_2_), 6.15 (d, J = 3.3 Hz, 1H, H-3), 6.97 (t, J = 8.4 Hz, 1H, H-5′), 7.07 (t, J = 7.1 Hz, 1H, H-6′), 7.23 (d, J = 2.0 Hz, 1H, H-2′), 7.34 (d, J = 8.1 Hz, 1H, H-7′), 7.60 (d, J = 7.8 Hz, 1H, H-4′), 10.29 (s, 1H, CONH), 10.88 (s, 1H, NH). ^13^C NMR (151 MHz, DMSO-*d*_6_) for the major *synperiplanar* conformer δ = 17.87 (cyclo-CH_3_), 20.77 (CH_3_), 29.11 (CH_2_), 29.15 (C-6), 29.55 (C-4), 39.96 (C-5), 108.32 (C-3′), 109.94 (=CH_2_), 111.28 (C-7′), 118.25 (C-5′), 118.67 (C-4′), 120.86 (C-6′), 123.80 (C-2′), 127.46 (C-2), 132.20 (C-3a’), 132.47 (C-3), 136.00 (C-7a’), 147.56 (C=), 149.04 (C=N), 173.40 (C=O); resolved signals for minor *antiperiplanar* conformer around the amide bond: 20.50 (CH_3_), 29.46 (C-6), 31.34 (CH_2_), 40.28 (C-5), 108.59 (C-3′), 110.20 (=CH), 111.33 (C7′), 118.29 (C-5′), 118.74 (C-4′), 120.97 (C-6′), 123.82 (C-2′), 127.18 (C-2), 132.40 (C-3a’), 133.23 (C-3), 136.08 (C-7a’), 147.65 (C=), 153.41 (C=N), 167.24 (C=O). HRMS (ESI) *m*/*z* calcd for C_20_H_24_N_3_O [M + H]^+^ 322.19139; found 322.19062.

*(R,E)-N’-(2-Methyl-5-(prop-1-en-2-yl)cyclohex-2-en-1-ylidene)-1H-indole-3-carbohydrazide* (**3h**). White solid; yield: 77%; m.p. 219–221 °C. ^1^H NMR (600 MHz, DMSO-*d*_6_) δ 1.77 (s, 3H, CH_3_), 1.92 (s, 3H, cyclo-CH_3_), 2.11 (tdd, J = 2.5, 11.3, 17.3 Hz, 1H, H-4), 2.15 (dd, J = 12.6, 16.1 Hz, 1H, H-6), 2.27 (td, J = 5.0, 17.2 Hz, 1H, H-4), 2.36 (tt, J = 4.0, 11.6 Hz, 1H, H-5), 3.01 (dd, J = 3.6, 16.4 Hz, 1H, H-6), 4.79 (t, J = 1.4 Hz, 1H, =CH_2_), 4.84 (s, 1H, =CH_2_), 6.17 (d, J = 5.0 Hz, 1H, H-3), 7.13 (ddd, J = 1.0, 7.0, 7.9 Hz, 1H, H-5′), 7.17 (ddd, J = 1.3, 6.9, 8.1 Hz, 1H, H-6′), 7.45 (d, J = 8.0 Hz, 1H, H-7′), 8.22 (br s, 1H, H-4′), 8.35 (br s, 1H, H-2′), 10.12 (s, 1H, CONH), 11.70 (s, 1H, NH). ^13^C NMR (151 MHz, DMSO-*d*_6_) δ = 18.34 (cyclo-CH_3_), 20.70 (CH_3_), 29.23 (C-6), 29.64 (C-4), 40.19 (C-5), 108.27 (brs, C-3′), 110.06 (=CH_2_), 111.77 (C-7′), 120.62 (C-5′), 121.38 (brs, C-4′), 122.05 (C-6′), 127.30 (brs, C-2), 132.43 (C-3a’), 132.65 (C-3), 135.62 (brs, C-7a’), 147.72 (C=). HRMS (ESI) *m*/*z* calcd for C_19_H_22_N_3_O [M + H]^+^ 308.17574; found 308.17513.

*(R,E)N’-(2-Methyl-5-(prop-1-en-2-yl)cyclohex-2-en-1-ylidene)isonicotinohydrazide* (**3i**). White solid; yield: 91%; m.p. 144–145 °C. Compound **3i** existed as a 1:0.34 mixture of synperiplanar and *antiperiplanar* conformers around the amide bond in DMSO-*d*_6_. ^1^H NMR (600 MHz, DMSO-*d*_6_) δ 1.74 (s, 3H, CH_3_), 1.87 (s, 3H, cyclo-CH_3_), 2.13 (t, J = 14.4 Hz, 1H, H-4), 2.19 (dd, J = 13.2, 16.2 Hz, 1H, H-6), 2.27 (d, J = 16.9 Hz, 1H, H-4), 2.37 (t, J = 11.8 Hz, 1H, H-5), 2.95 (dd, J = 2.8, 16.4 Hz, 1H, H-6), 4.78 (s, 1H, =CH_2_), 4.81 (s, 1H, =CH_2_), 6.27 (d, J = 3.4 Hz, 1H, H-3), 7.73 (d, J = 5.1 Hz, 2H, H-2′ and H-6′), 8.73 (d, J = 5.2 Hz, 2H, H-3′ and H-5′), 10.88 (s, 1H, NH). Signals corresponding to the minor *antiperiplanar* conformer around the amide bond were observed at δ 1.57 (s, 3H, cyclo-CH_3_), 3.01 (d, J = 16.4 Hz, 1H, H-6), 6.13 (br s, 1H, H-3), 7.59 (br s, 2H, Ar-H), 8.66 (br s, 2H, Ar-H), 11.10 (s, 1H, NH). ^13^C NMR (151 MHz, DMSO-*d*_6_) δ = 17.88 (cyclo-CH_3_), 20.47 (CH_3_), 29.81 (C-4), 30.15 (C-6), 40.36 (C-5), 110.27 (=CH_2_), 121.81 (C-2′ and C-6′), 132.27 (C-2), 135.03 (C-3), 141.34 (C-1′), 147.56 (C=), 150.06 (C-3′ and C-5′), 158.57 (C=N), 162.27 (C=O). HRMS (ESI) *m*/*z* calcd for C_16_H_20_N_3_O [M + H]^+^ 270.16009; found 270.15942.

#### 3.2.4. Single-Crystal X-Ray Diffraction

The structures of **3i** was confirmed by single-crystal X-ray analysis (Figure 2). X-ray analysis was performed on a Bruker D8 Venture diffractometer, using Mo radiation and CMOS Photon II detector and processed using the APEX3 software package (Bruker AXS GmbH, Karlsruhe, Germany) [70]. The data were processed with Apex 5 software and corrected for absorption using SADABS [70]. The structure was solved by direct method using the program SHELXTL, version 1.0.1818 [46] and were refined by full-matrix least squares technique on *F*^2^ using anisotropic displacement parameters. The non-hydrogen atoms were refined anisotropically. In these compounds, the nitrogen H atoms were located from Fourie difference map will all other H atoms were placed on position calculated geometrically, with isotropic displacement parameters set to 1.2 times the equivalent isotropic *U* values of the parent carbon atoms. The crystallographic data for **3i** (CCDC 2564483) have been submitted to the Cambridge Crystallographic Data Centre (https://www.ccdc.cam.ac.uk/data_request/cif, accessed on 29 March 2026). Table 9 displays the most important data collection and refinement indicators for **3i**. Tables S1–S4 (Supplementary Materials) display the main geometrical parameters of compound **3i**, e.g., bond length values, bond angles, torsion angles and hydrogen bonding interactions.

### 3.3. In Silico Studies

#### 3.3.1. Molecular Docking

Molecular docking studies were performed using the Molecular Operating Environment software package (MOE, version 2024). The X-ray structure of human butyrylcholinesterase (hBChE) was retrieved from the Protein Data Bank (PDB ID: 3DJY) and used as the receptor model. The crystallographic structure was inspected in MOE, and all non-essential crystallographic species were removed before receptor preparation. Hydrogen atoms were added, and the protonation state of the protein was assigned at pH 7.0, 0.15 M NaCl and 300 K using the protonation procedure implemented in MOE. The R configurations of compounds **3a**–**i** and the R/S configurations of donepezil were generated in MOE and protonated according to their expected ionization states at pH 7.0. The ligands were energy-minimized using the AMBER12:EHT force field. For each ligand, conformational sampling was performed using the LowModeMD method. Conformations within an energy window of 7 kcal/mol above the lowest-energy conformer were retained for the docking procedure. The binding site was defined within the active-site gorge of hBChE. The Site Finder algorithm implemented in MOE was used to identify and characterize the binding pocket. The docking region included the catalytic site, the oxyanion-hole region, the choline-binding/aromatic region, the acyl-binding pocket and the peripheral region of the gorge. The following residues were considered during binding-site inspection and pose analysis: Trp82, Asp70, Tyr332, Ser198, His438, Glu325, Gly116, Gly117, Ala199, Trp231, Leu286, Val288 and Phe329. Ligand placement was carried out using the Alpha PMI placement method. The generated poses were initially scored with the London dG scoring function. For each ligand, the best 30 poses from the placement stage were retained and further refined using the induced-fit protocol. During refinement, receptor atoms within 6 A of the ligand were allowed to relax, while the remaining receptor atoms were kept restrained. The refinement step was performed using the AMBER12:EHT force field and reaction-field solvation model. The refined poses were rescored using the GBVI/WSA dG scoring function. Final pose ranking was based on the resulting score-function values. Donepezil was used as an internal reference and was treated as a racemic reference compound. The R and S configurations of donepezil were docked separately under the same conditions, giving score-function values of −7.15 and −7.10 kcal/mol, respectively. For the synthesized compounds **3a**–**i**, only the R-form docking outputs were used for the discussion, in accordance with the stereochemical form evaluated experimentally. Representative R-form poses were selected according to the docking score, correct occupation of the hBChE active-site gorge and absence of evident steric artifacts. Two-dimensional ligand–receptor interaction maps were generated in MOE for the selected poses.

#### 3.3.2. ADMET Prediction

ADME screenings were performed using the online tool SwissADME of the Swiss Institute of Bioinformatics Institute (https://www.swissadme.ch/ accessed on 18 June 2026). The SwissADME tool is based on multiple linear regression, binary classification, and SVM algorithms performed over large data sets of known inhibitors/non-inhibitors, as well as on substrates/non-substrates [46]. The integrated tool Ketcher, version 3.7.0 (https://lifescience.opensource.epam.com/ketcher/index.html accessed on 18 June 2026) was used to obtain the molecular files and to simplify the representation of each compound. The Indigo toolkit (https://lifescience.opensource.epam.com/indigo/ accessed on 18 June 2026) was used to convert the compounds representation into a canonical SMILES description. The web service ProTox-III (https://tox.charite.de/protox3/ accessed on 18 June 2026) was used to predict the toxicity of the synthesized compounds. The tool incorporates computer models based on chemical similarities statistics, complex machine-learning algorithms [56]. The models were trained on specific databases of real data to estimate the accuracy and the specific confidence range (probability) at each classification.

### 3.4. Cytotoxicity Assay of Cannabidiol and Novel CBD Analogues

#### 3.4.1. Cell Culture Conditions

The human neuroblastoma cell line SH-SY5Y was obtained from the European Collection of Authenticated Cell Cultures (ECACC; supplied through Sigma-Aldrich), and the murine Neuro-2a neuroblastoma cell line was obtained from the American Type Culture Collection (ATCC). Both human SH-SY5Y and murine Neuro-2a neuroblastoma cell lines were cultured in RPMI 1640 medium supplemented with 10% fetal bovine serum (FBS) and 2 mM L-glutamine. Cells were maintained under standard cell culture conditions at 37 °C in a humidified atmosphere containing 5% CO_2_. Cell cultures were routinely monitored under an inverted microscope, and the growth medium was replaced as required to maintain optimal nutrient conditions and ensure consistent cell viability and proliferation throughout the experimental period.

#### 3.4.2. MTT Cell Viability Assay

The cytotoxic effects of cannabidiol (CBD) and nine newly synthesized CBD analogues (**3a**–**i**, and CBD) were evaluated in human SH-SY5Y and murine Neuro-2a neuroblastoma cell lines. Cells were seeded in 96-well culture plates at a density of 1–2 × 10^4^ cells per well and incubated for 24 h at 37 °C in a humidified atmosphere containing 5% CO_2_ to allow for cell attachment and stabilization. Stock solutions of all tested compounds were prepared in DMSO and subsequently diluted in complete culture medium to obtain the desired final working concentrations; the final DMSO concentration was kept constant and did not exceed 0.5% (*v*/*v*) in any well, including the vehicle control. After the initial 24 h incubation period, the culture medium was replaced with 100 μL of medium containing the tested compounds. Cells were exposed to final concentrations of 1, 10, 50, 100, 300, 400, and 500 μM, while both cell lines were treated under identical experimental conditions. Each concentration was tested in technical triplicate within a single concentration–response experiment. Following treatment, cells were incubated for an additional 24 h under standard culture conditions. Cell viability was subsequently assessed using the MTT assay. Briefly, MTT reagent (3-(4,5-dimethylthiazol-2-yl)-2,5-diphenyltetrazolium bromide; 10 mg/mL in PBS) was added to each well, and plates were incubated for 3 h at 37 °C to allow mitochondrial dehydrogenases of viable cells to reduce MTT to insoluble formazan crystals. After incubation, the medium was carefully removed, and formazan crystals were dissolved in 100 μL DMSO with gentle shaking. Absorbance was measured using a microplate reader (Synergy 2, BioTek Instruments, Inc., Winooski, VT, USA) at 570 nm, with 690 nm used as a reference wavelength for background correction. After subtraction of the blank, cell viability was expressed as a percentage of the vehicle-treated control (set to 100%), and IC_50_ values were determined from dose–response curves using nonlinear regression analysis (log[inhibitor] vs. normalized response). Curve fitting and IC_50_ estimation were performed using GraphPad Prism version 8 (GraphPad Software, San Diego, CA, USA). IC_50_ values are reported together with their 95% confidence intervals (CI) derived from the profile-likelihood method of the nonlinear regression. Because IC_50_ values were obtained from a single concentration–response experiment, no between-experiment statistics (e.g., ANOVA) were applied, and the reported confidence intervals reflect the precision of the individual curve fit.

### 3.5. Cholinesterase Inhibition Assays

AChE and BChE inhibitory activity was measured using the microplate assay described by Ellman et al. [60] with the modifications added by López et al. [61]. Compounds were tested at concentrations between 10^−3^ and 10^−6^ M. First, all compounds were dissolved in MeOH (or MeOH:DMSO 4:1 *v*/*v* in one instance to ensure solubility) in a concentration of 5 mg/mL. Then, they were diluted using phosphate buffer (PBS) (8 mM K_2_HPO_4_, 2.3 mM NaH_2_PO_4_, 0.15 M NaCl, pH 7.5) to provide the concentration range needed. The largest and second-largest concentration solutions were put in an ultrasound bath for 30 min at room temperature to ensure that precipitates do not interfere with subsequent measurements. AChE from *Electrophorus electricus* and BChE from equine serum were used with a substrate solution of 5,5′-dithiobis(2-nitrobenzoic acid) (DTNB) with acetylthiocholine iodide (ATCI) or butyrylthiocholine iodide (BTCI), respectively (0.04 M Na_2_HPO_4_, 0.2 mM DTNB, 0.24 mM ATCI or BTCI, pH 7.5). Fifty microliters of enzyme solution (0.25 U/mL, in PBS) and 50 μL of the tested compound solution were added to the wells. Incubation of the plates was performed at room temperature for 30 min. Then, 100 μL of substrate solution were added to start the enzymatic reaction. The absorbances were read in a microplate reader (BIOBASE, ELISA-EL10A, Jinan, China) at 405 nm after 5 min for AChE and 10 min for BChE. Enzyme activity was calculated as an inhibition percentage compared to an assay including PBS instead of an inhibitor. Donepezil was used as a positive control. Data were analyzed using the software package Prism 3 (Graph Pad Inc., San Diego, CA, USA). The IC_50_ values were measured in triplicate and the results are presented as means ± SD. Inhibition percentages of 500 μM and enzyme selectivity indices are also reported.

### 3.6. Antioxidant Assays

#### 3.6.1. DPPH Radical Scavenging Activity

The free radical scavenging activity of the synthesized compounds was evaluated using the DPPH assay according to Grochowski et al. [71] with slight modifications. Briefly, 50 μL of compound solutions in methanol (1.0, 0.5, 0.25, 0.125, and 0.0625 mg/mL) were mixed with 100 μL of DPPH methanolic solution (2 mg/mL). After incubation in the dark at room temperature for 30 min, the absorbance was measured at 517 nm. The percentage of DPPH radical scavenging activity was calculated using the following equation:

DPPH radical scavenging activity (%) = ((Abs_contr._ − Abs_sample_)/Abs_contr._) ×100,

where Abs_contr._ is the absorbance of DPPH radical with 50 μL MeOH, Abs_sample_ is the absorbance of DPPH radical solution mixed with sample. Ascorbic acid was used as a positive control. The IC_50_ values were determined from concentration–response curves. All measurements were performed in triplicate (n = 3).

#### 3.6.2. ABTS Radical Scavenging Assay

The ABTS radical scavenging activity was determined according to Grochowski et al. [71] with minor modifications. The ABTS radical cation (ABTS•^+^) was generated by mixing equal volumes of 7 mM ABTS and 2.4 mM potassium persulfate solutions and allowing the mixture to react in the dark at room temperature for 14 h. Prior to analysis, the solution was diluted with methanol to obtain an absorbance of 0.605 ± 0.01 at 734 nm. Compound solutions (50 μL; 1.0, 0.5, 0.25, 0.125, and 0.0625 mg/mL) were mixed with 200 μL of ABTS working solution and incubated for 5 min at room temperature. Absorbance was measured at 734 nm. The ABTS scavenging capacity of the compound was calculated as follows:

ABTS radical scavenging activity (%) = ((Abs_contr._ − Abs_sample_)/Abs_contr._) ×100,

where Abs_contr._ is the absorbance of ABTS radical with 300 μL MeOH, Abs_sample_ is the absorbance of ABTS radical solution mixed with the sample. IC_50_ values were calculated from concentration–response curves. Ascorbic acid served as a positive control. All experiments were carried out in triplicate (n = 3) and the results are presented as means ± SD.

#### 3.6.3. Ferric-Reducing/Antioxidant Power (FRAP)

The ferric-reducing antioxidant power (FRAP) assay was performed according to Benzie and Strain [56] with slight modifications. The FRAP reagent was freshly prepared by mixing 25 mL of 300 mM acetate buffer (pH 3.6), 2.5 mL of 10 mM TPTZ solution in 40 mM HCl, and 2.5 mL of 20 mM FeCl_3_·6H_2_O solution. The reagent was preheated to 37 °C before use. An aliquot of 10 μL of each compound solution (5 mM in methanol) was mixed with 300 μL of FRAP reagent and incubated for 30 min in the dark. The absorbance of the resulting Fe^2+^–TPTZ complex was measured at 593 nm. A Trolox calibration curve was constructed, and the results were expressed as mmol Trolox equivalents per mmol of compound (mmol TE/mmol). Ascorbic acid was used as a positive control. All measurements were performed in triplicate (n = 3) and the results are presented as means ± SD.

#### 3.6.4. Determination of Antioxidant Activity in Linoleic Acid System by the FTC Method

The inhibitory effect of the tested compounds on lipid peroxidation was assessed using the ferric thiocyanate (FTC) method as described by Takao et al. [72] with slight modifications. The reaction mixture consisted of 200 μL of compound solution (1 mg/mL in methanol), 200 μL of linoleic acid emulsion (25 mg/mL in 99% ethanol), and 400 μL of 50 mM phosphate buffer (pH 7.6). The mixtures were incubated at room temperature in the dark. At 24 h intervals, 10 μL aliquots of the reaction mixture were transferred to 200 μL of 70% ethanol and 10 μL of 30% ammonium thiocyanate solution. Subsequently, 10 μL of 20 mM ferrous chloride solution prepared in 3.5% hydrochloric acid was added. After 3 min, the absorbance was measured at 500 nm. Measurements were continued until the absorbance of the control sample reached its maximum value. Ascorbic acid (1 mg/mL) was used as a positive control. All experiments were performed in triplicate (n = 3).

IC_50_ values were calculated by nonlinear regression analysis using concentration–response curves. Statistical analyses were performed using GraphPad Prism version 8 (GraphPad Software, San Diego, CA, USA). Differences were considered statistically significant at *p* < 0.05.

## 4. Conclusions

The present study provides an initial multitarget pharmacological profiling of a new series of CBD-like hydrazone derivatives through integrated in silico and in vitro investigations. Although the current findings provide only preliminary evidence of their multitarget anti-Alzheimer potential, they identify promising lead structures for further development. Additional mechanistic studies, expansion of the chemical space to validate and refine the preliminary structure–activity relationship (SAR) observations, and comprehensive in vivo evaluation will be required to further establish their therapeutic potential. All synthesized derivatives (**3a**–**i**) exhibited favorable physicochemical characteristics and complied with Lipinski’s Rule of Five, indicating good drug-likeness and potential oral bioavailability. Their calculated logP values (3.49–4.80) suggest an appropriate balance between lipophilicity and blood–brain barrier permeability, a prerequisite for central nervous system therapeutics. Biological evaluation revealed a clear preference of the synthesized compounds for BChE over AChE inhibition. Among the tested derivatives, compounds **3f** and **3b** emerged as the most potent and selective BChE inhibitors, exhibiting IC_50_ values of 1.67 ± 0.11 μM and 1.94 ± 0.30 μM, respectively. Notably, compound **3f** contains a tertiary amine moiety resembling a key pharmacophoric feature of donepezil, which may contribute to its enhanced biological activity. Cytotoxicity studies further demonstrated a favorable in vitro safety profile, with all derivatives displaying substantially lower toxicity than CBD in neuronal cell models. In particular, compounds **3a**, **3b**, **3f**, **3h**, and **3i** combined low cytotoxicity with promising pharmacological properties, supporting their further investigation. Antioxidant activity evaluation demonstrated complementary radical-scavenging, ferric-reducing, and lipid peroxidation inhibitory activities, with compounds **3b**, **3e**, **3f**, and **3h** showing the highest overall activity. Among them, compounds **3b** and **3f** exhibited the most balanced antioxidant profile, combining efficient free-radical scavenging with strong protection against lipid peroxidation, a key process associated with oxidative neuronal damage in Alzheimer’s disease.

Taken together, the favorable drug-like properties, selective BChE inhibition, low cytotoxicity, and multifunctional antioxidant activity identify compounds **3b** and **3f** as promising lead structures warranting further optimization and comprehensive biological evaluation as potential anti-Alzheimer agents. Ongoing studies are currently evaluating the neuroprotective effects of the most promising derivatives against H_2_O_2_-induced oxidative stress in SH-SY5Y cells. These investigations will be followed by experimental BBB permeability assessment, in vivo validation of their multitarget pharmacological profile and efficacy in Alzheimer’s disease models, and structural optimization through analogue development to expand the chemical space and further refine the preliminary SAR.

## Figures and Tables

**Table 1:** Molecular descriptors and drug-likeness parameters of compounds **3a**–**i.**

Compd.	MW	MR	TPSA	Heavy Atoms	Aromatic Heavy Atoms	Rot. Bonds	HBA	HBD	logP	Lipinski R5
**3a**	284.35	85.35	61.69	21	6	4	3	2	4.80	0
**3b**	284.35	85.35	61.69	21	6	4	3	2	3.80	0
**3c**	300.35	87.37	81.92	22	6	4	4	3	3.51	0
**3d**	298.38	89.82	50.69	22	6	5	3	1	4.10	0
**3e**	298.38	89.82	50.69	22	6	5	3	1	4.10	0
**3f**	311.42	97.53	44.70	23	6	5	2	1	4.16	0
**3g**	321.42	99.77	57.25	24	9	5	2	2	4.51	0
**3h**	337.42	101.7	66.48	25	9	5	3	2	4.59	0
**3i**	269.34	81.12	54.35	20	6	4	3	1	3.49	0
**CBD**	314.46	99.85	40.46	23	6	6	2	2	5.85	1

**Table 2:** Predicted ADME, pharmacokinetic, and drug-likeness properties of the synthesized compounds.

Compd.	Water Solubility	LogS	GI Abs.	BBB	PGp Subs.	CYP 1A2 Inh.	CYP 2C19 Inh.	CYP 2C9 Inh.	CYP 2D6 Inh.	CYP 3A4 Inh.	Log Kp (cm/s)	Synth. Accessib.
**3a**	Soluble	−3.77	High	Yes	No	No	Yes	Yes	No	Yes	−5.53	3.75
**3b**	Soluble	−3.77	High	Yes	No	Yes	Yes	Yes	No	Yes	−5.53	3.75
**3c**	Soluble	−3.98	High	No	No	No	No	Yes	No	Yes	−5.49	3.82
**3d**	Soluble	−3.99	High	Yes	No	No	Yes	Yes	No	Yes	−5.39	3.79
**3e**	Soluble	−3.99	High	Yes	No	No	Yes	Yes	No	Yes	−5.39	3.82
**3f**	Mod. soluble	−4.15	High	Yes	No	Yes	Yes	Yes	No	Yes	−5.36	3.95
**3g**	Mod. soluble	−4.26	High	Yes	No	Yes	Yes	Yes	No	Yes	−5.46	3.90
**3h**	Mod. soluble	−4.38	High	Yes	No	Yes	Yes	Yes	No	Yes	−5.53	3.86
**3i**	Soluble	−3.24	High	Yes	No	No	Yes	Yes	No	No	−5.95	3.83
CBD	Mod. soluble	−5.69	High	Yes	No	No	Yes	Yes	Yes	Yes	−3.59	4.05

**Table 3:** Predicted toxicity and pharmacokinetic-related properties of compounds **3a**–**i** obtained using ProTox-III.

Target	3a		3b		3c		3d		3e		3f		3g		3h		3i	
	Pred	Prob	Pred	Prob	Pred	Prob	Pred	Prob	Pred	Prob	Pred	Prob	Pred	Prob	Pred	Prob	Pred	Prob
***Hepatotoxicity***	Active	0.53	Active	0.53	Active	0.52	Active	0.53	Active	0.53	Inactive	0.57	Inactive	0.50	Active	0.56	Active	0.57
***Neurotoxicity***	Active	0.51	Active	0.51	Inactive	0.65	Active	0.57	Active	0.57	Active	0.58	Active	0.72	Active	0.59	Active	0.60
***Nephrotoxicity***	Inactive	0.53	Inactive	0.53	Active	0.60	Inactive	0.56	Inactive	0.56	Inactive	0.72	Inactive	0.59	Inactive	0.50	Inactive	0.67
***Resp. toxicity***	Active	0.59	Active	0.59	Active	0.51	Active	0.57	Active	0.57	Active	0.71	Active	0.75	Active	0.61	Active	0.72
***Cardiotoxicity***	Inactive	0.58	Inactive	0.58	Inactive	0.62	Inactive	0.56	Inactive	0.56	Inactive	0.57	Inactive	0.67	Inactive	0.70	Inactive	0.63
***Carcinogenicity***	Active	0.68	Active	0.68	Active	0.61	Active	0.58	Active	0.58	Active	0.69	Active	0.67	Active	0.56	Active	0.74
***Immunotox.***	Inactive	0.97	Inactive	0.75	Inactive	0.81	Inactive	0.86	Inactive	0.57	Inactive	0.89	Inactive	0.97	Active	0.52	Inactive	0.98
***Mutagenicity***	Active	0.54	Active	0.54	Inactive	0.52	Active	0.54	Active	0.54	Active	0.57	Inactive	0.56	Inactive	0.51	Inactive	0.57
***Cytotoxicity***	Inactive	0.78	Inactive	0.78	Inactive	0.80	Inactive	0.76	Inactive	0.76	Inactive	0.71	Inactive	0.75	Inactive	0.72	Inactive	0.81
***BBB-barrier***	Active	0.69	Active	0.69	Inactive	0.52	Active	0.71	Active	0.71	Active	0.87	Active	0.82	Active	0.71	Active	0.90
***Ecotoxicity***	Inactive	0.56	Inactive	0.56	Inactive	0.59	Active	0.55	Active	0.55	Active	0.53	Inactive	0.51	Active	0.50	Inactive	0.50
***Clinical toxicity***	Active	0.58	Active	0.58	Active	0.61	Active	0.51	Active	0.51	Active	0.55	Active	0.58	Active	0.55	Active	0.54
***Nutr. toxicity***	Inactive	0.54	Inactive	0.54	Inactive	0.54	Inactive	0.54	Inactive	0.54	Inactive	0.59	Inactive	0.50	Inactive	0.53	Inactive	0.53
***CYP1A2***	Inactive	0.69	Inactive	0.69	Inactive	0.62	Inactive	0.67	Inactive	0.67	Inactive	0.79	Inactive	0.63	Inactive	0.59	Inactive	0.80
***CYP2C19***	Inactive	0.71	Inactive	0.71	Inactive	0.76	Inactive	0.64	Inactive	0.64	Inactive	0.78	Inactive	0.61	Inactive	0.62	Inactive	0.68
***CYP2C9***	Active	0.51	Active	0.51	Inactive	0.51	Active	0.60	Active	0.60	Active	0.55	Active	0.58	Active	0.64	Active	0.55
***CYP2D6***	Inactive	0.61	Inactive	0.61	Inactive	0.55	Inactive	0.61	Inactive	0.61	Inactive	0.56	Active	0.53	Inactive	0.54	Inactive	0.57
***CYP3A4***	Inactive	0.60	Inactive	0.60	Inactive	0.66	Inactive	0.51	Inactive	0.51	Inactive	0.61	Active	0.62	Inactive	0.52	Inactive	0.56
***CYP2E1***	Inactive	0.99	Inactive	0.99	Inactive	0.99	Inactive	0.99	Inactive	0.99	Inactive	0.99	Inactive	0.99	Inactive	0.99	Inactive	0.99
																		
***Pred. LD_50_*** **(mg/kg)**	**324.00**		**324.00**		**324.00**		**324.00**		**324.00**		**370.00**		**341.00**		**370.00**		**510.00**	
***Pred. Tox. Class***	**4**		**4**		**4**		**4**		**4**		**4**		**4**		**4**		**4**	

**Table 4:** Docking score-function values and main close-contact residues for the selected representative R-form BChE docking outputs of compounds **3a**–**i**. Donepezil R and Donepezil S are included as separate internal reference configurations, because donepezil was used experimentally as a racemate.

Compound	Score-Function Value (kcal/mol)	Main Close-Contact Residues in hBChE
Donepezil R, reference configuration	−7.15	Pro285, Trp82, Tyr332, Tyr128, Asp70, Gly115, Thr120, Gly116, His438, Phe329
Donepezil S, reference configuration	−7.10	Pro285, Trp82, Ala328, Tyr128, Tyr332, Asp70, Thr120, Gly116, Phe329, His438
**3f**	−7.07	Ser198, Tyr128, Gly117, Leu286, Trp231, Glu197, Phe329, Ala199, Trp82, His438
**3g**	−6.98	Gly116, Ser287, Thr120, Leu286, Ser198, Glu197, Val288, Gly117, Tyr128
**3e**	−6.87	Ser198, Glu197, Gly117, His438, Trp82, Ser287, Leu286, Gly116
**3d**	−6.67	Glu197, Tyr128, Phe329, Trp82, Asp70, Gly116, His438, Tyr332
**3a**	−6.64	Gly115, Glu197, Tyr332, Trp82, Phe329, Tyr128, His438, Gly116
**3h**	−6.62	Trp82, Ala328, Thr120, Phe329, His438, Trp430, Gly116, Glu197
**3c**	−6.54	Trp231, Val288, Trp82, Gly117, His438, Phe329, Gly116, Leu286
**3i**	−6.21	Ser198, Gly117, Leu286, Ala199, Trp82, Phe329, Glu197, His438
**3b**	−6.12	Ser198, Glu197, Thr120, Pro285, Gly439, His438, Gln119, Trp82, Gly116

Donepezil was used experimentally as a racemate; therefore, its R and S configurations were docked separately and are shown as internal reference configurations. Listed residues represent close contacts from heavy-atom ligand–protein distance matrices.

**Table 5:** Cytotoxic activity of compounds **3a**–**i** against SH-SY5Y and Neuro-2a cells.

Compd.	Formula	SH-SY5Y IC_50_ (µM)	95% CI (Profile Likelihood) *	Neuro-2a IC_50_ (µM)	95% CI (Profile Likelihood) *
**3a**	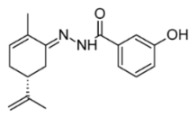	239.90	197.2–278.1	357.70	271.5–401.2
**3b**	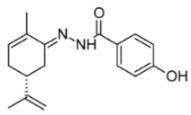	>500	n.a.	101.10	93.50–111.23
**3c**	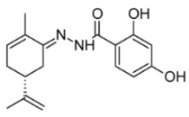	68.57	63.38–75.63	97.15	72.51–110.6
**3d**	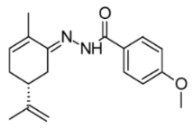	58.55	48.08–70.15	161.20	104.9–493.9
**3e**	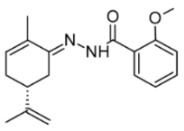	82.33	67.91–123.2	66.02	55.99–85.15
**3f**	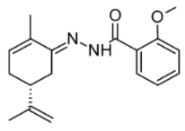	160.40	116.95–189.23	78.44	62.80–83.15
**3g**	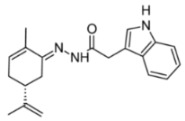	70.10	66.08–74.41	72.45	65.56–78.88
**3h**	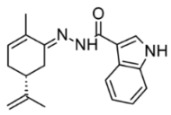	274.80	172.6–286.23	130.00	77.12–139.00
**3i**	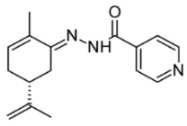	302.10	298.5–316.9	63.76	58.98–68.84
**CBD**		15.40	11.23–19.59	17.52	11.92–24.43

* IC_50_ values were calculated by nonlinear regression analysis and are expressed as μM. Values were obtained from a single concentration–response experiment performed in technical triplicate; the 95% confidence intervals (CI) were estimated by the profile-likelihood method and reflect the precision of the curve fit rather than biological replication. >500, IC_50_ above the highest tested concentration (500 μM), i.e., estimated by extrapolation; n.a., not applicable (IC_50_ outside the tested concentration range).

**Table 6:** In vitro inhibition of AChE from *Electrophorus electricus* and BChE from equine serum by the synthesized compounds and their selectivity indices towards BChE.

Compd.	*ee*AChE		*eq*BChE		Selectivity index (SI) for BChE
	Inhibition (%) at 500 μM (* 1 μM)	IC_50_, (μM)	Inhibition (%) at 500 μM (** 100 μM)	IC_50_, (μM)	(IC_50_ (AChE)/IC_50_ (BChE) *
**3a**	20.73 ± 1.34	>1000	83.24 ± 0.97	30.17 ± 2.00	>33.14
**3b**	22.94 ± 1.47	>1000	76.45 ± 0.87	1.94 ± 0.30	>515.46
**3c**	20.19 ± 1.55	974.47 ± 12.87	77.07 ± 1.95	99.81 ± 2.40	9.76
**3d**	6.76 ± 1.01	>1000	82.42 ± 1.56	29.24 ± 0.45	>34.20
**3e**	25.14 ± 2.68	>1000	79.49 ± 2.25	56.02 ± 2.61	>17.85
**3f**	60.62 ± 1.65	316.20 ± 36.76	90.83 ± 2.98	1.67 ± 0.11	189.34
**3g**	13.11 ± 2.01	>1000	47.57 ± 2.73	544.50 ± 26.23	1.84
**3h**	52.83 ± 2.31	426.03 ± 64.61	64.20 ± 5.27	268.90 ± 7.50	1.58
**3i**	4.20 ± 1.51	>1000	81.94 ± 3.04	51.26 ± 0.56	>19.50
**Donepezil**	* 82.11 ± 1.83	0.099 ± 0.005	** 78.70 ± 0.79	8.49 ± 0.49	0.012 (AChE-selective)

SI = IC_50_(AChE)/IC_50_(BuChE). Values > 1 indicate BChE selectivity, whereas values < 1 indicate AChE selectivity. * Percentage inhibition of AChE determined at a concentration of 500 μM for the tested compounds and at 1 μM for donepezil. Donepezil exhibited pronounced AChE selectivity (SI = 0.012). ** Percentage inhibition of BChE determined at a concentration of 100 μM for both the tested compounds and donepezil.

**Table 7:** DPPH, ABTS radical scavenging and FRAP activity of the synthesized compounds.

Compd.	DPPH IC_50_ [mM]	ABTS IC_50_ [mM]	FRAP [mMTE/mM] *
**3a**	nd	8.69 ± 0.85	10.03 ± 0.30
**3b**	8.52 ± 0.88	0.37 ± 0.01	26.25 ± 0.57
**3c**	nd	17.66 ± 0.21	19.64 ± 0.11
**3d**	nd	9.12 ± 0.51	12.07 ± 1.30
**3e**	13.13 ± 1.10	0.22 ± 0.01	34.76 ± 3.94
**3f**	27.22 ± 1.23	5.35 ± 0.52	13.71 ± 0.54
**3g**	nd	5.51 ± 0.68	25.24 ± 3.44
**3h**	nd	6.96 ± 0.76	51.47 ± 3.46
**3i**	nd	7.72 ± 0.43	9.79 ± 0.83
**Ascorbic acid**	0.28 ± 0.02	0.11 ± 0.01	183.64 ± 1.71
**CBD**	4.13 ± 0.03	0.10 ± 0.01	-

* FRAP values are expressed as mmol Trolox equivalents (TE) per mmol of tested compound (mmol TE/mmol compound). Higher values indicate greater ferric-reducing antioxidant power; nd—no detectable activity sufficient for IC_50_ calculation under the experimental conditions.

**Table 8:** Inhibition of the lipid peroxidation (FTC method) of the synthesized compounds.

Compd.	Absorbance (Mean ± SD)				
	1st Day	2nd Day	3rd Day	4th Day	5th Day
**3a**	0.86 ± 0.01	1.08 ± 0.02	1.12 ± 0.08	1.16 ± 0.03	1.17 ± 0.06
**3b**	1.02 ± 0.05	1.02 ± 0.02	0.99 ± 0.01	0.99 ± 0.02	0.99 ± 0.02
**3c**	0.95 ± 0.03	0.95 ± 0.06	1.02 ± 0.08	1.19 ± 0.03	1.00 ± 0.02
**3d**	0.95 ± 0.02	1.18 ± 0.01	1.02 ± 0.02	1.11 ± 0.02	1.12 ± 0.02
**3e**	0.98 ± 0.01	1.07 ± 0.02	1.10 ± 0.01	1.13 ± 0.04	1.17 ± 0.03
**3f**	0.89 ± 0.01	0.92 ± 0.02	0.92 ± 0.03	0.93 ± 0.07	0.94 ± 0.06
**3g**	0.85 ± 0.01	1.08 ± 0.06	1.15 ± 0.04	1.20 ± 0.01	1.43 ± 0.03
**3h**	0.96 ± 0.01	1.09 ± 0.02	1.12 ± 0.01	1.13 ± 0.01	1.43 ± 0.04
**3i**	0.87 ± 0.01	1.20 ± 0.02	1.23 ± 0.01	1.28 ± 0.01	1.29 ± 0.08
**Ascorbic acid**	0.80 ± 0.01	0.81 ± 0.02	0.83 ± 0.03	0.83 ± 0.02	0.83 ± 0.03
**CBD**	0.88 ± 0.01	1.12 ± 0.02	1.10 ± 0.09	1.08 ± 0.01	1.02 ± 0.03
**Control**	0.89 ± 0.01	1.09 ± 0.01	1.18 ± 0.01	1.20 ± 0.02	1.33 ± 0.01

**Table 9:** Crystal data and structure refinement parameters for **3i**.

Identification code	**3i**
Empirical formula	C_16_H_19_N_3_O
Formula weight	269.34
Temperature/K	290
Crystal system	monoclinic
Space group	P2_1_
a/Å	9.4476(6)
b/Å	17.4289(9)
c/Å	9.5195(6)
α/°	90
β/°	102.622(2)
γ/°	90
Volume/Å^3^	1529.61(16)
Z	4
ρ_calc_g/cm^3^	1.170
μ/mm^−1^	0.075
F(000)	576.0
Crystal size/mm^3^	0.3 × 0.35 × 0.1
Radiation	MoKα (λ = 0.71073)
2Θ range for data collection/°	4.384 to 57.466
Reflections collected	44 963
Independent reflections	7885
Data consistency/merging indicators	R_int_ = 0.0619, R_sigma_ = 0.0469
Data/restraints/parameters	7885/1/374
Goodness-of-fit on F^2^	1.035
Final R indexes [I>=2σ (I)]	R_1_ = 0.0475, wR_2_ = 0.0936
Final R indexes [all data]	R_1_ = 0.1038, wR_2_ = 0.1165
Largest diff. peak/hole/e Å^−3^	0.14/−0.15
Flack parameter	0.1(6)
CCDC number	2 564 483

## Data Availability

Data is contained within the article and Supplementary Materials. The crystallographic data for **3i** (CCDC 2564483) have been submitted to the Cambridge Crystallographic Data Centre (www.ccdc.cam.ac.uk/data_request/cif, accessed on 29 July 2026).

## Supplementary Materials

The following supporting information can be downloaded at: https://www.mdpi.com/article/10.3390/molecules31152657/s1, Figures S1–S62: NMR spectra and HRMS; Table S1–S4: X-ray data.
