# Supplementary material for: Design, Synthesis, In Silico and In Vitro Pharmacological Profiling of Cannabidiol-like Synthetic Analogues as Multi-Target Anti-Alzheimer’s Agents
Source: Molecules. 2026 Jul 30;31(15):2657. doi: 10.3390/molecules31152657 (PMC13468590; doi:10.3390/molecules31152657)
Supplement: Supplementary file 1 [file molecules-31-02657-s001.zip › molecules-4434815-supplementary.pdf]

# Supporting information

# Design, Synthesis, In Silico and In Vitro Pharmacological Profiling of Cannabidiol-like Synthetic Analogues as Multi-Target Anti-Alzheimer's Agents

**Boris P. Stoyanov [1], Borislav Georgiev [2], Denitsa Stefanova [1], Virginia Tzankova [1], Elena Kalcheva-Yovkova [3], Nikolay Vassilev [4], Miroslav Rangelov [4], Nadezhda Todorova [2], Dimitrina Zheleva-Dimitrova [5], Boris Shivachev [6] and Violina T. Angelova [7,*]**

[1] Department of Pharmacology, Pharmacotherapy and Toxicology, Faculty of Pharmacy, Medical University of Sofia, 1000 Sofia, Bulgaria; borisstoyanov03@gmail.com (B.P.S.); denitsa.stefanova@pharmfac.mu-sofia.bg (D.S.); virginia_tzankova@yahoo.com (V.T.)
[2] Institute of Biodiversity and Ecosystem Research, Bulgarian Academy of Sciences, 1113 Sofia, Bulgaria; bobogeorgiev5@gmail.com (B.G.); nadeshdahr@gmail.com (N.T.)
[3] Faculty of Computer Systems and Technologies, Technical University–Sofia, 1000 Sofia, Bulgaria; elena@tu-sofia.bg
[4] Institute of Organic Chemistry with Centre of Phytochemistry, Bulgarian Academy of Sciences, 1113 Sofia, Bulgaria; nikolay.vassilev@orgchm.bas.bg (N.V.), marangelov@gmail.com (M.R.)
[5] Department of Pharmacognosy, Faculty of Pharmacy, Medical University of Sofia, 1000 Sofia, Bulgaria; dzheleva@pharmfac.mu-sofia.bg
[6] Institute of Mineralogy and Crystallography "Acad. Ivan Kostov", Bulgarian Academy of Sciences, 1113 Sofia, Bulgaria; blshivachev@gmail.com
[7] Department of Chemistry, Faculty of Pharmacy, Medical University of Sofia, 1000 Sofia, Bulgaria
* Correspondence: v.stoyanova@pharmfac.mu-sofia.bg

## $^{1}H$ NMR, $^{13}C$ NMR, DEPT-135 NMR, 2D COSY NMR, 2D HSQC NMR, 2D HMBC NMR spectra and HRMS (Figures S1-S62)

## Single-crystal X-ray diffraction analysis of compound 3i. Tables S1-S4

1. ($R$,$E$)-3-hydroxy-$N'$-(2-methyl-5-(prop-1-en-2-yl)cyclohex-2-en-1-ylidene)benzohydrazide, **3a**

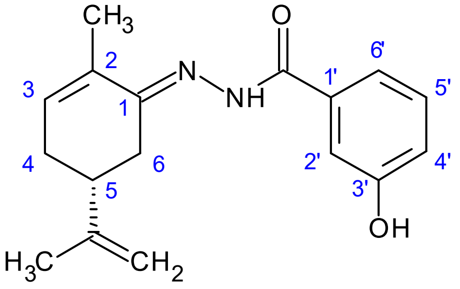

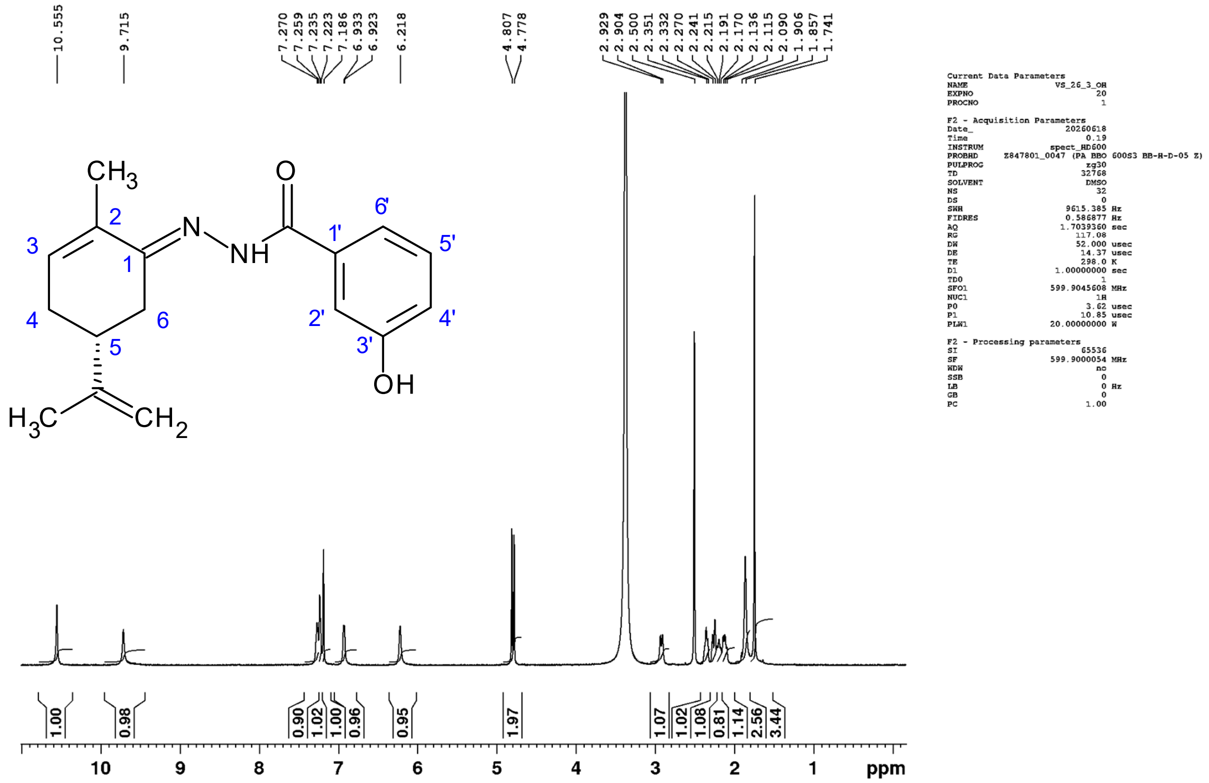


**Figure S1.** $^{1}$H NMR spectrum of **3a** in DMSO-$d_6$.

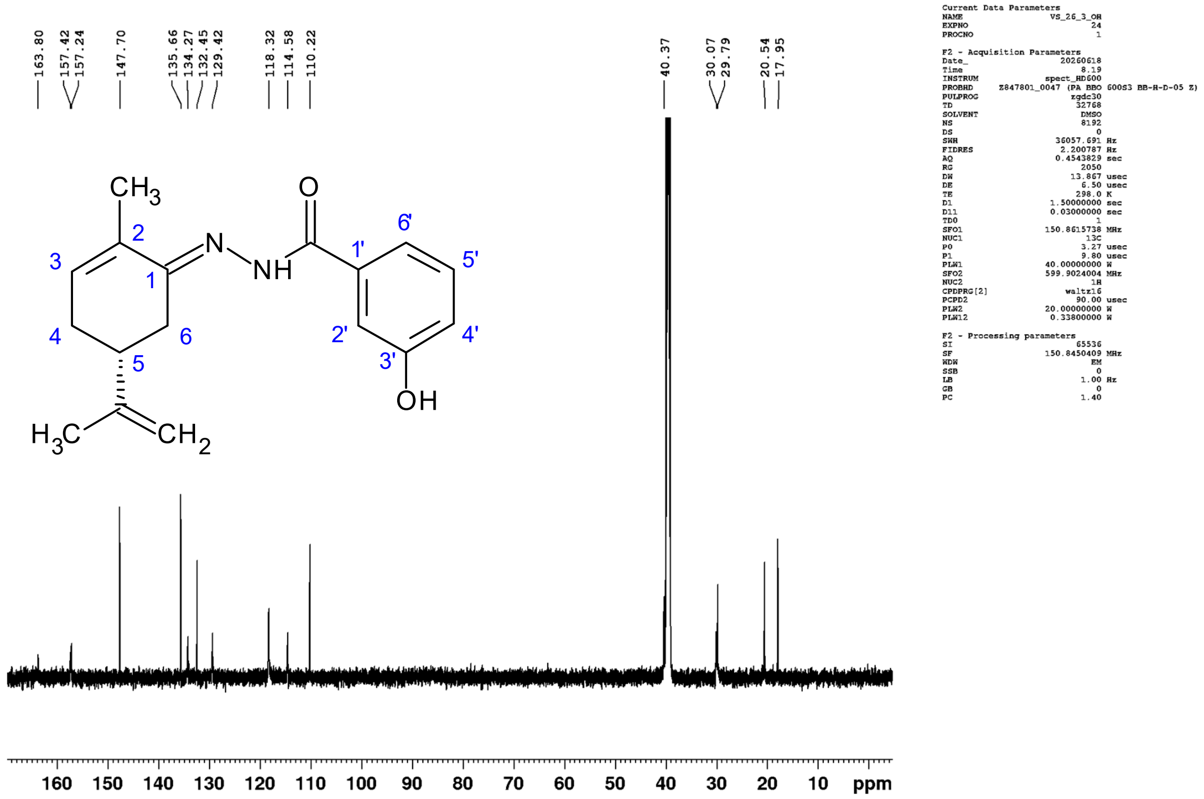


Figure S2. $^{13}$C NMR spectrum of **3a** in DMSO-$d_6$.

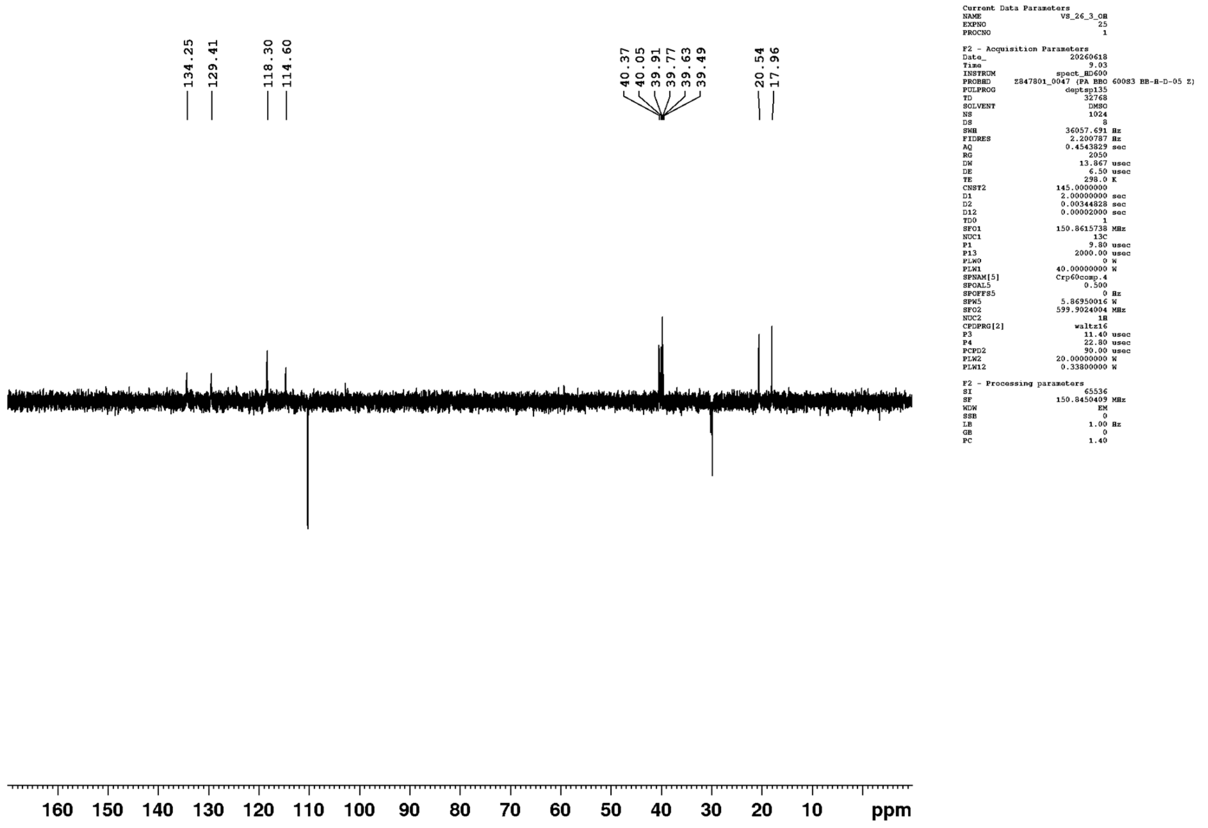


**Figure S3.** DEPT-135 NMR spectrum of **3a** in DMSO-$d_6$.

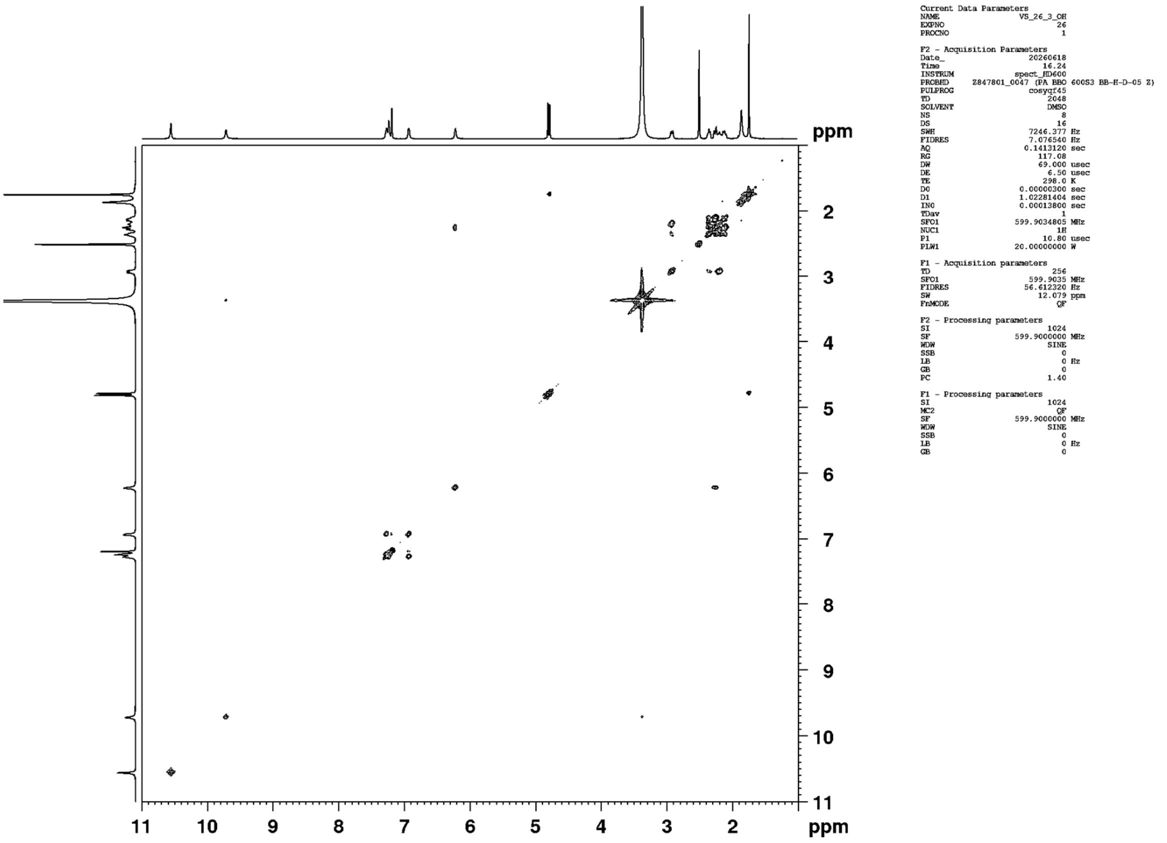


**Figure S4.** 2D COSY NMR spectrum of **3a** in DMSO-$d_6$.

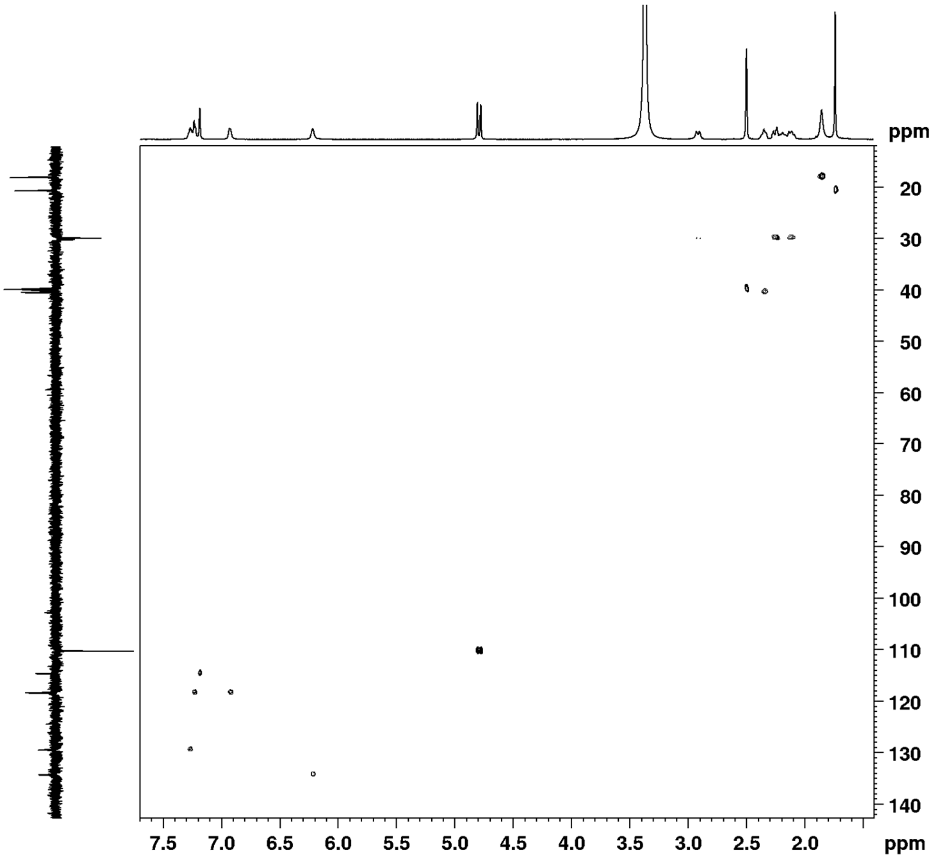


**Figure S5.** 2D HSQC NMR spectrum of **3a** in DMSO-$d_6$.

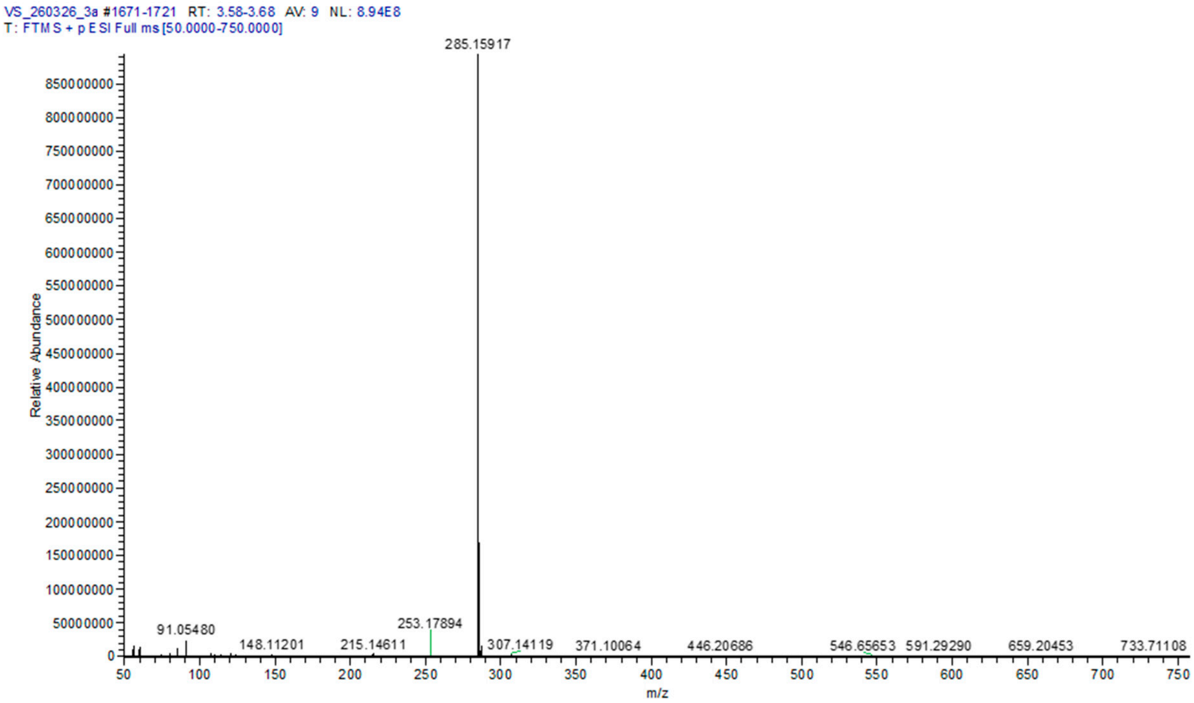


**Figure S6**. HRMS of **3a.**

2. (*R*,*E*)-4-hydroxy-*N*'-(2-methyl-5-(prop-1-en-2-yl)cyclohex-2-en-1-ylidene)benzohydrazide, **3b**

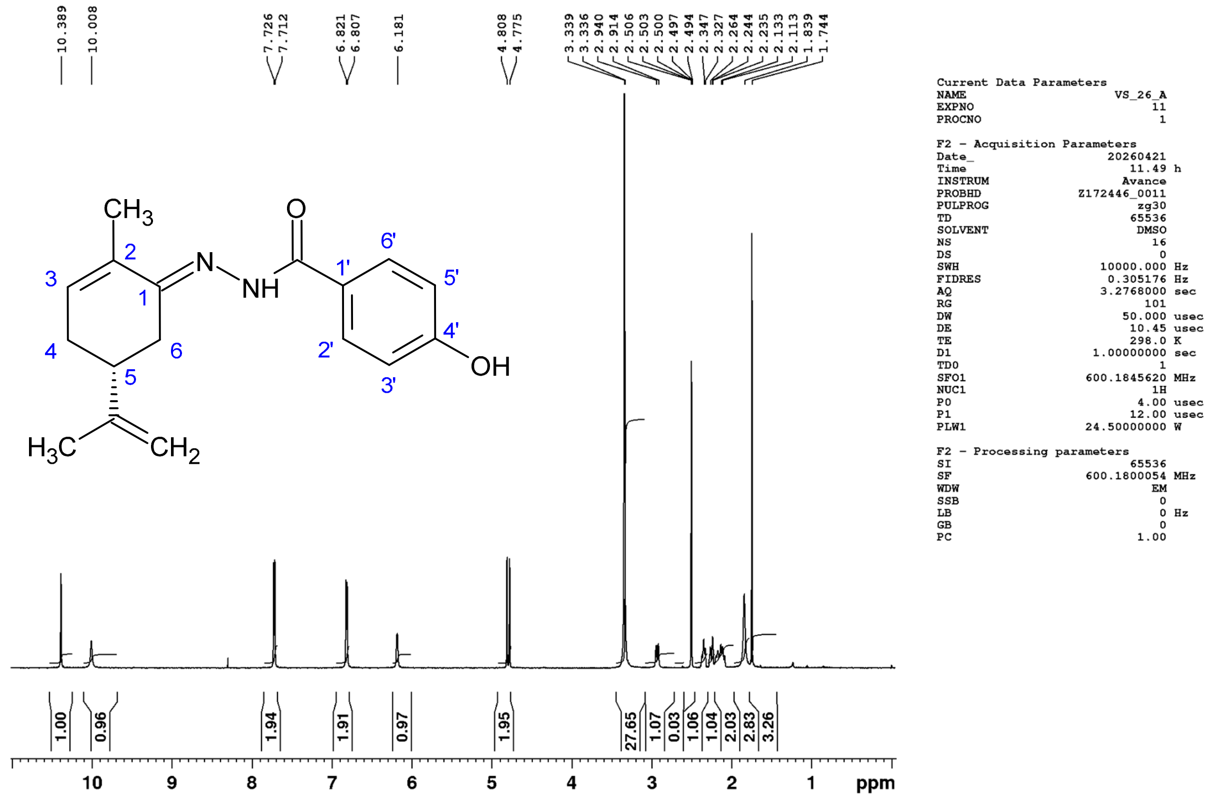


**Figure S7.** $^{1}H$ NMR spectrum of **3b** in DMSO-$d_6$.

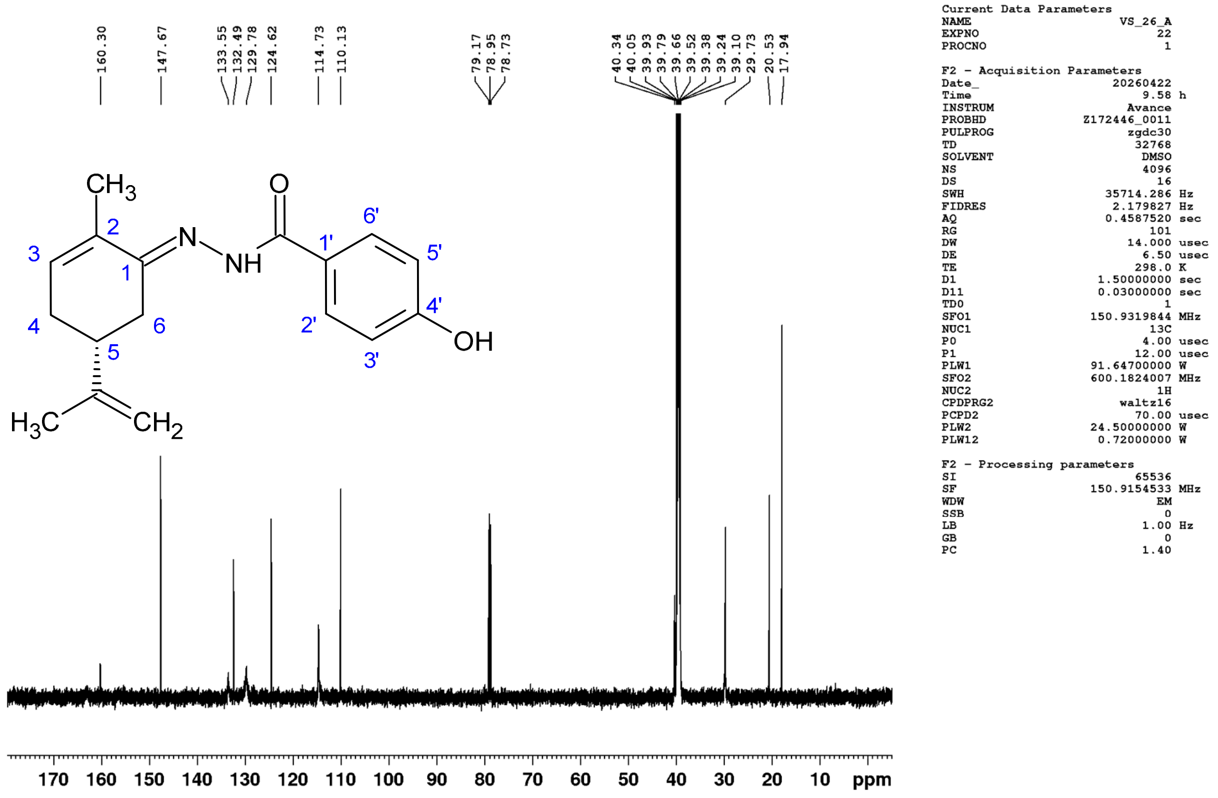


**Figure S8.** $^{13}C$ NMR spectrum of **3b** in DMSO-$d_6$.

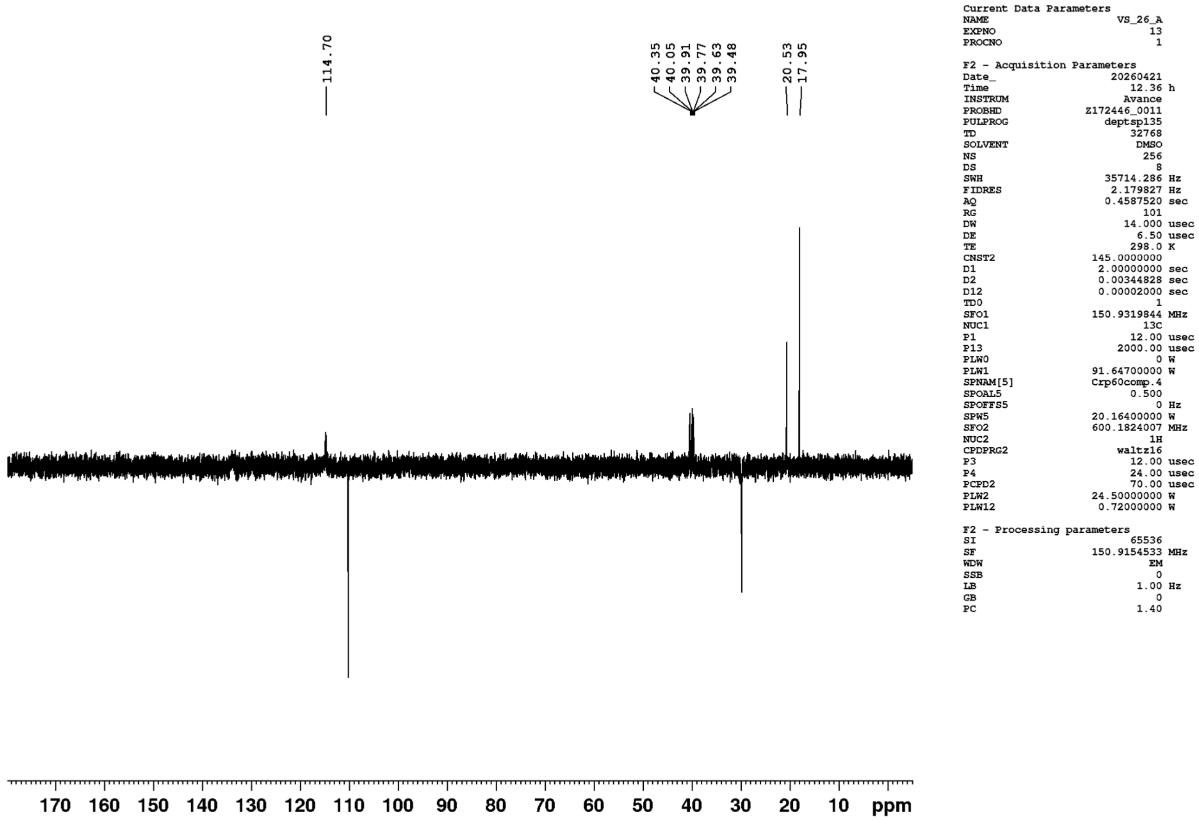


**Figure S9.** DEPT-135 NMR spectrum of **3b** in DMSO-$d_6$.

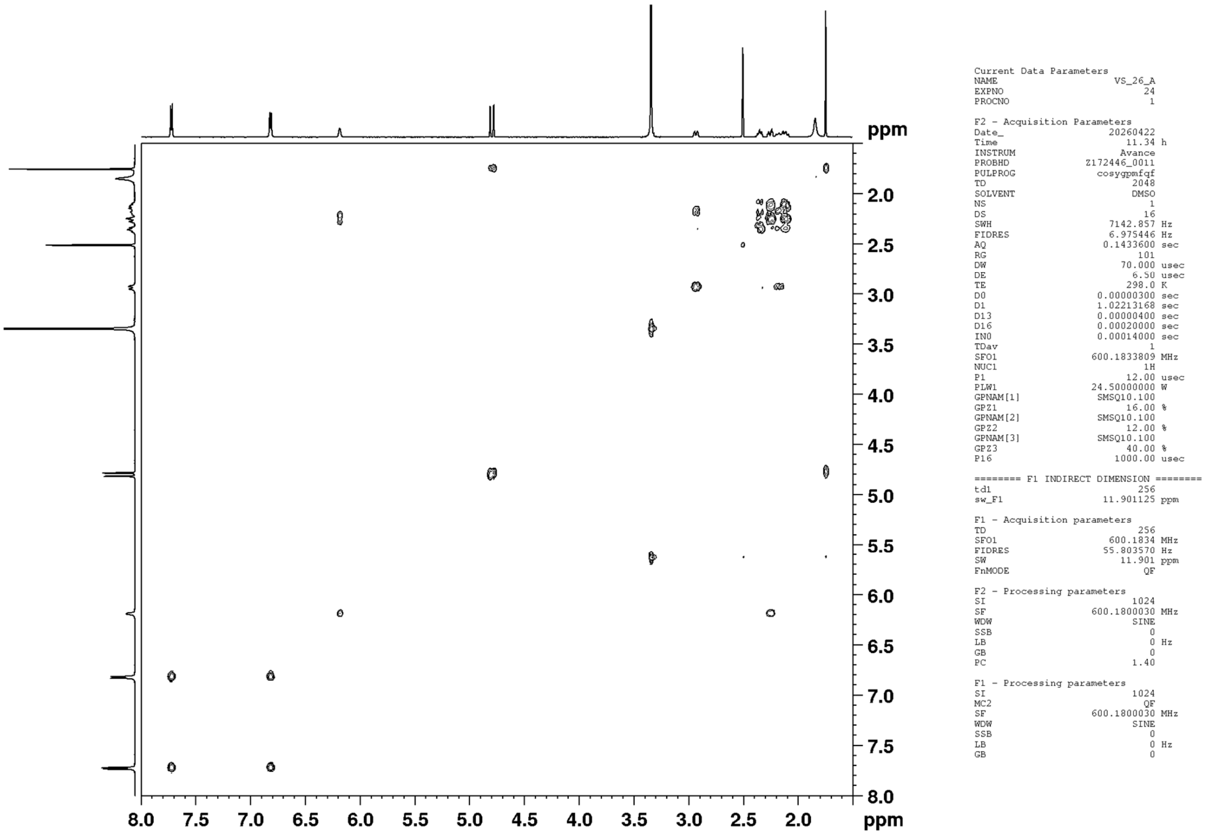


**Figure S10.** 2D COSY NMR spectrum of **3b** in DMSO-$d_6$.

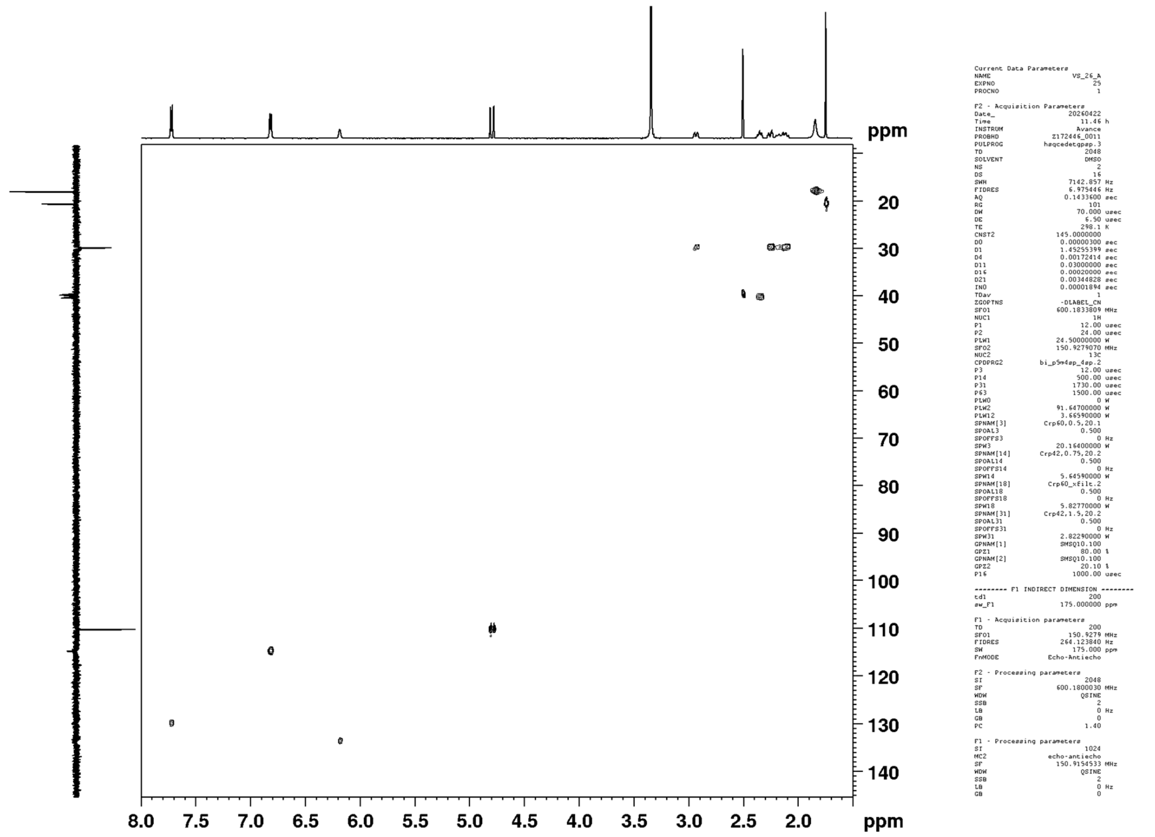


**Figure S11.** 2D HSQC NMR spectrum of **3b** in DMSO-$d_6$.

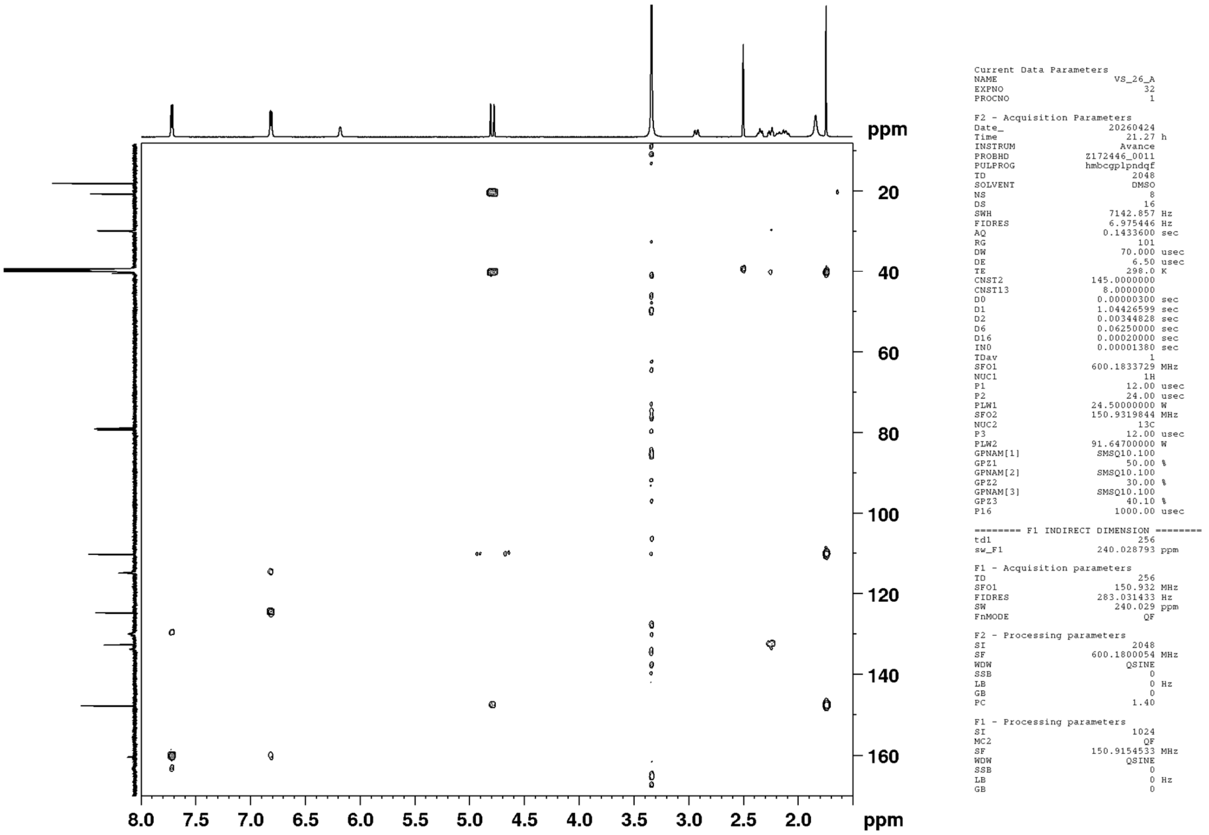


**Figure S12.** 2D HMBC NMR spectrum of **3b** in DMSO-$d_6$.

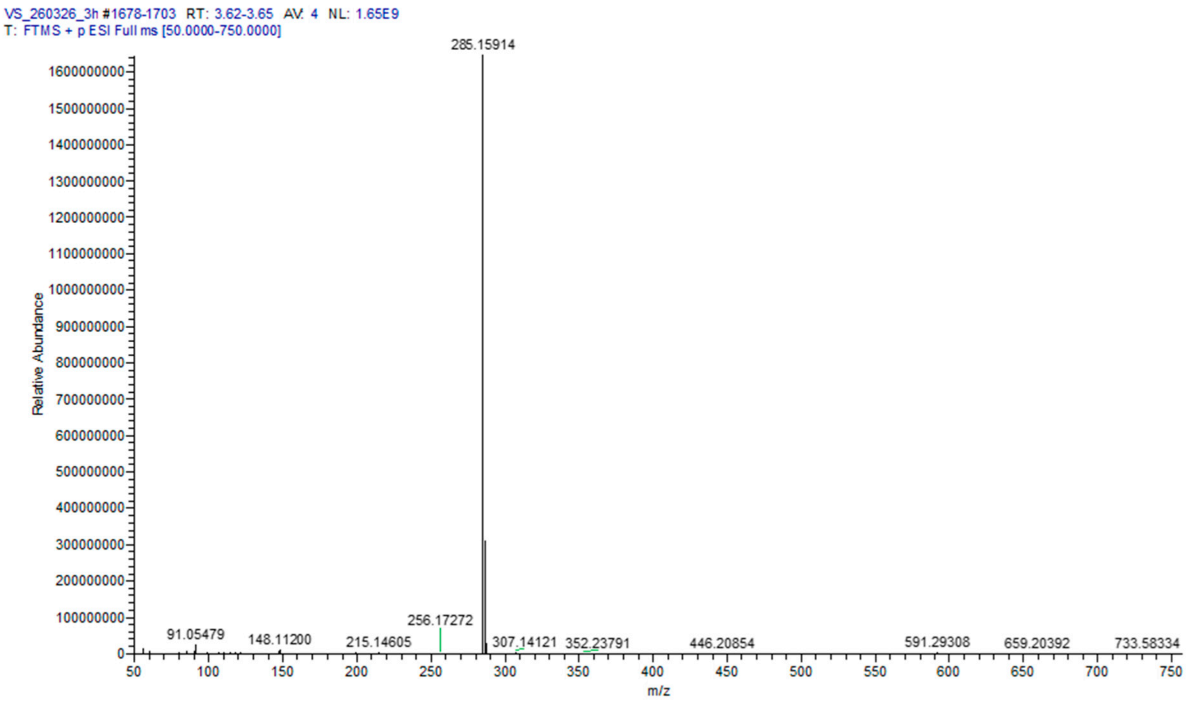


**Figure S13.** HRMS of **3b**.

3. (*R*,*E*)-2,4-dihydroxy-*N'*-(2-methyl-5-(prop-1-en-2-yl)cyclohex-2-en-1-ylidene) benzohydrazide, **3c**

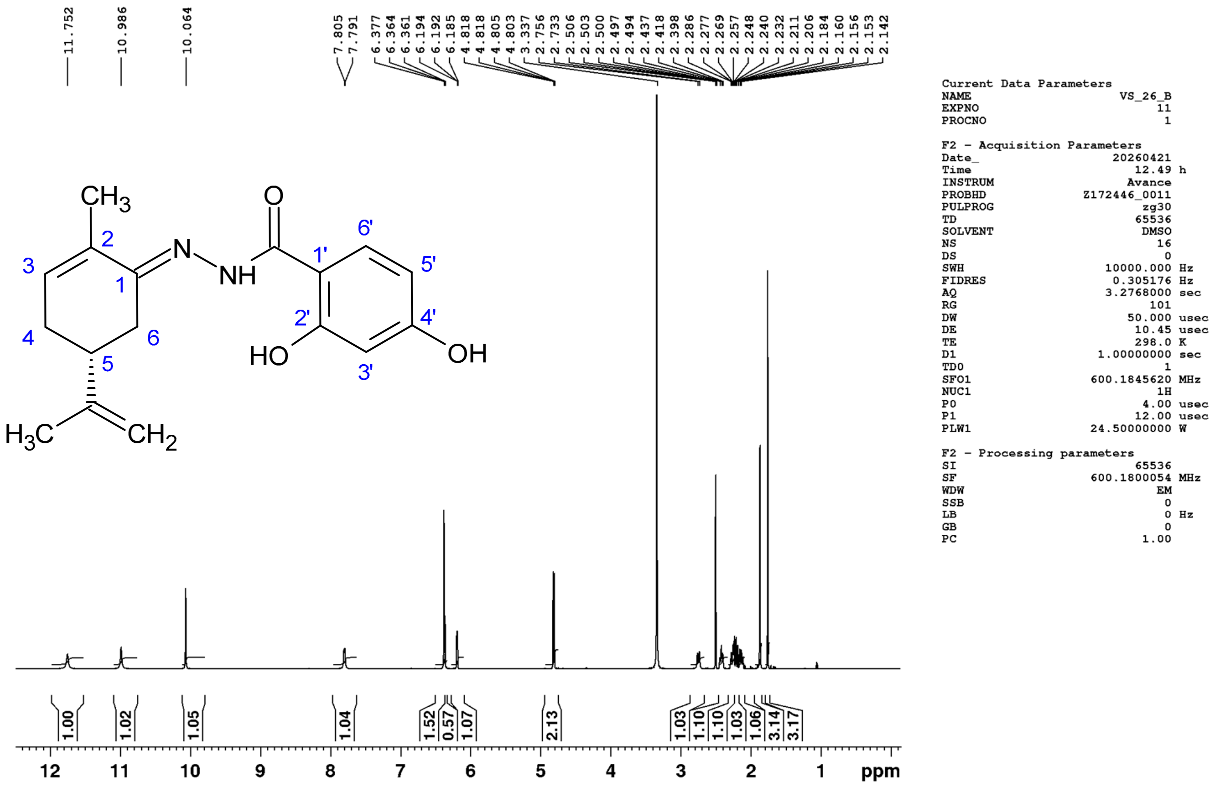


**Figure S14.** $^{1}$H NMR spectrum of **3c** in DMSO-$d_6$.

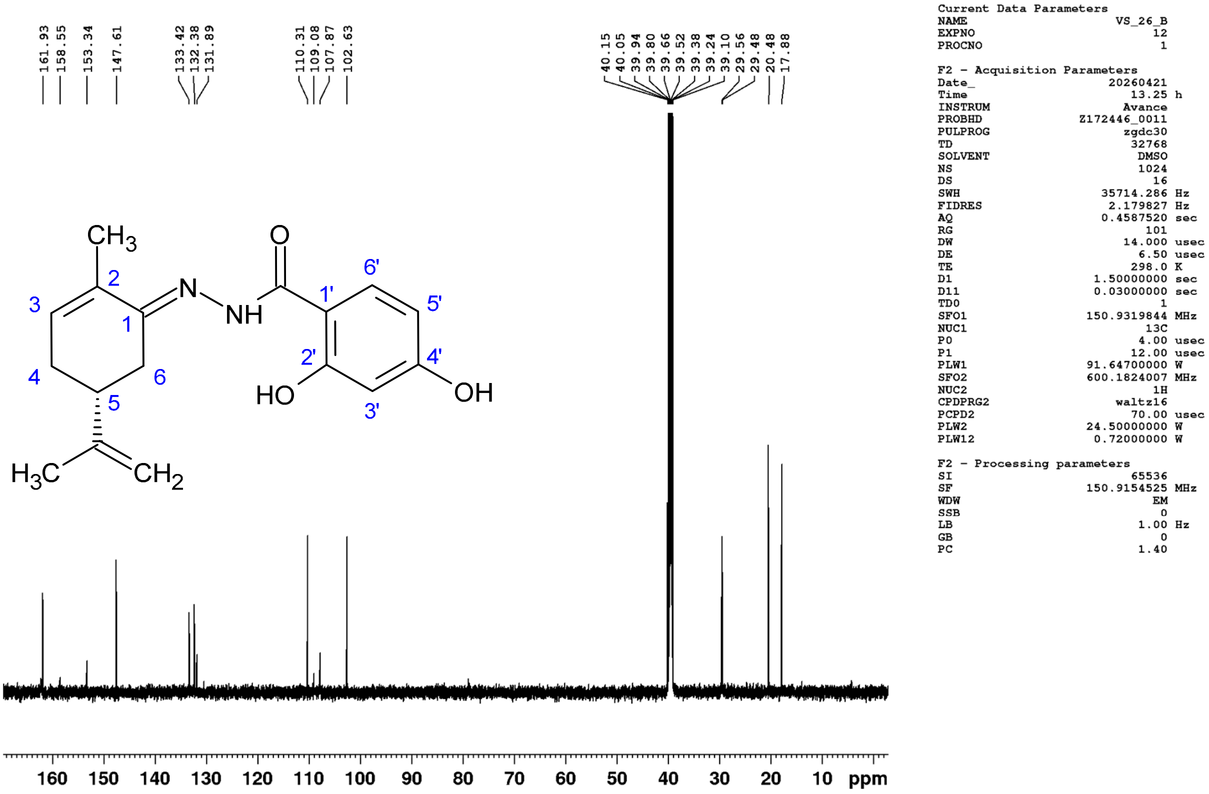


**Figure S15.** $^{13}$C NMR spectrum of **3c** in DMSO-$d_6$.

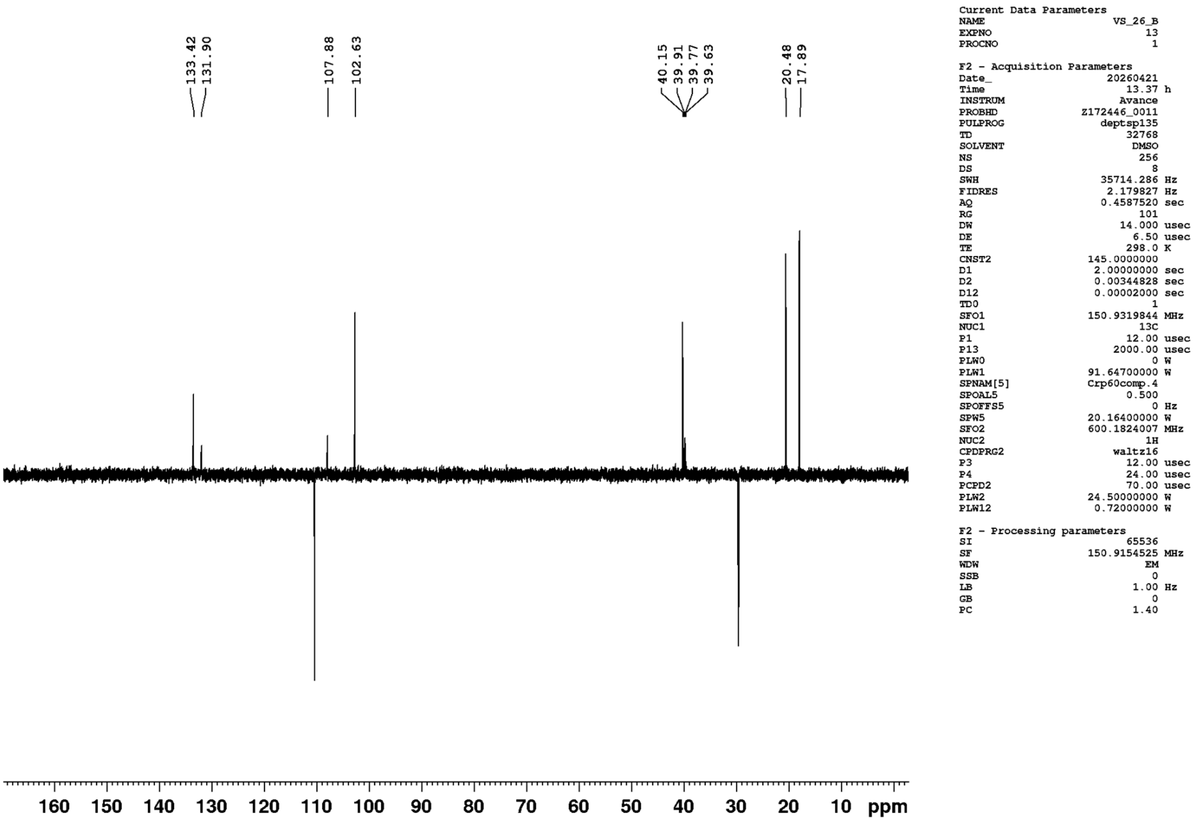


**Figure S16.** DEPT-135 NMR spectrum of **3c** in DMSO-$d_6$.

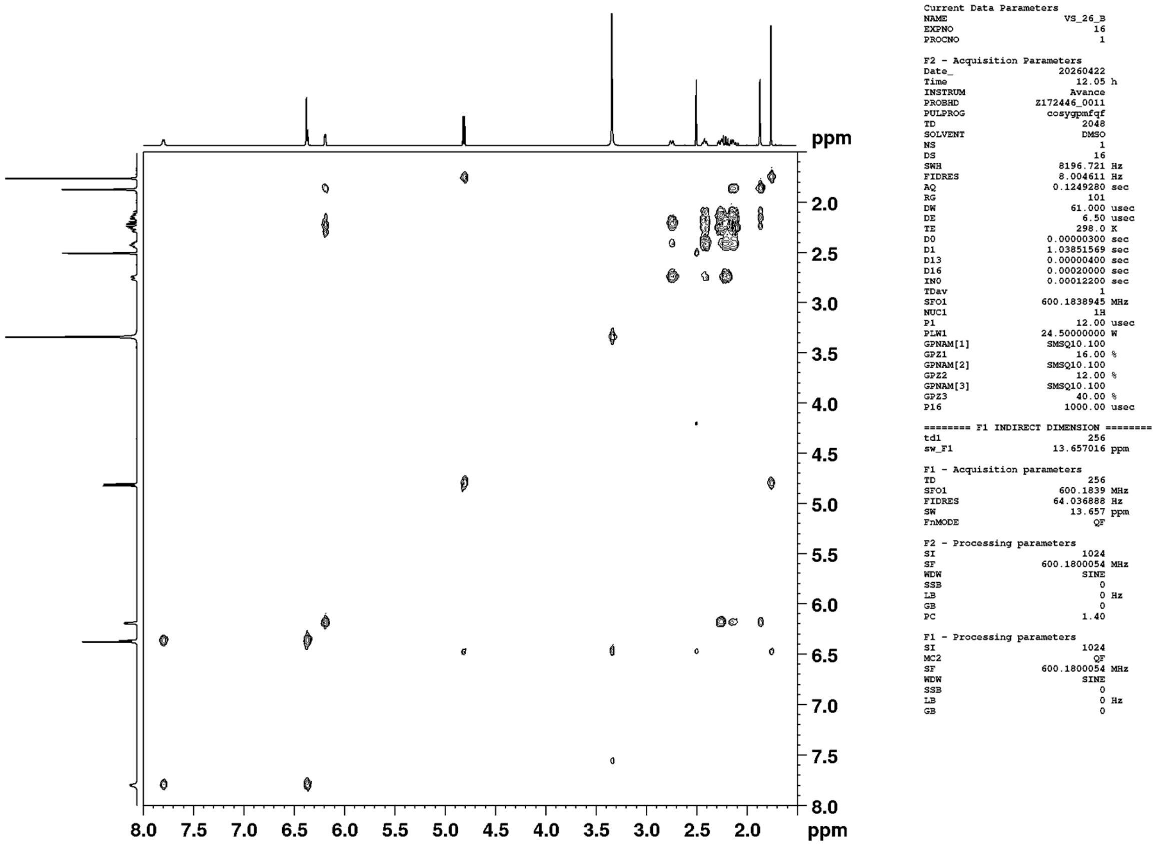


**Figure S17.** 2D COSY NMR spectrum of **3c** in DMSO-$d_6$.

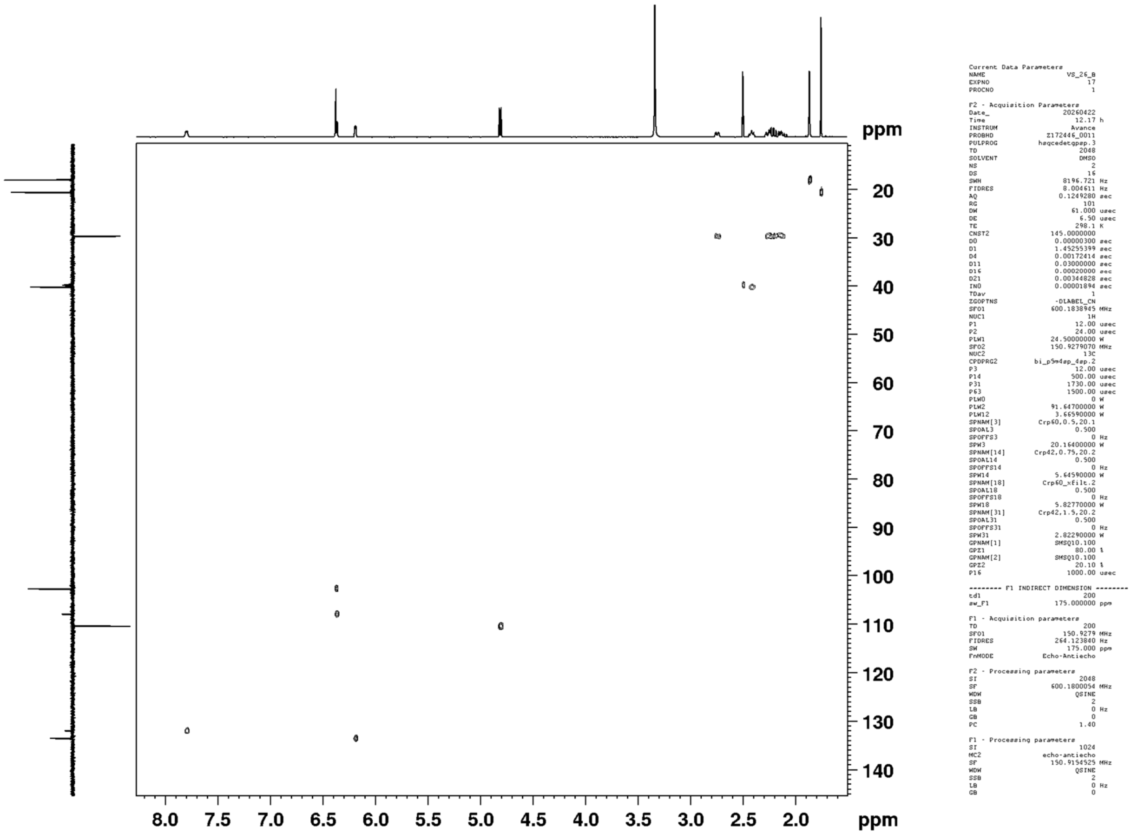


**Figure S18.** 2D HSQC NMR spectrum of **3c** in DMSO-$d_6$.

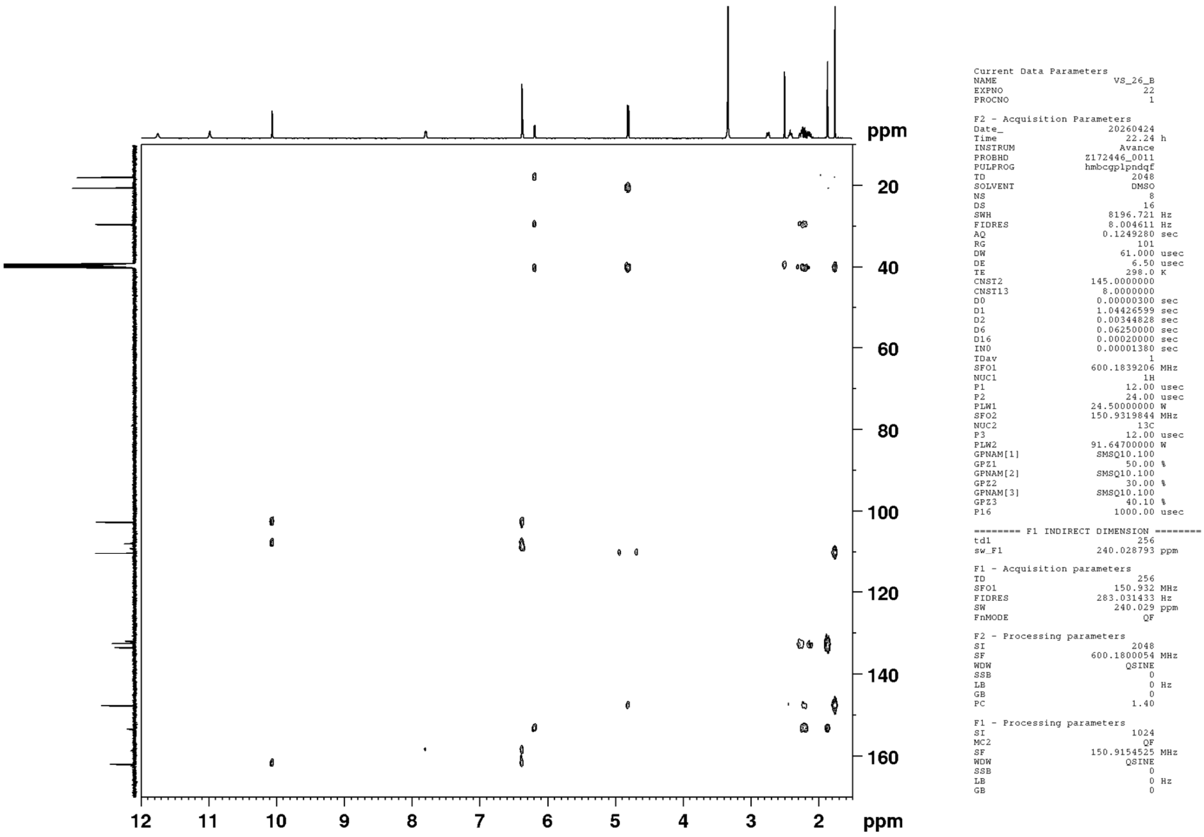


**Figure S19.** 2D HMBC NMR spectrum of **3c** in DMSO-$d_6$.

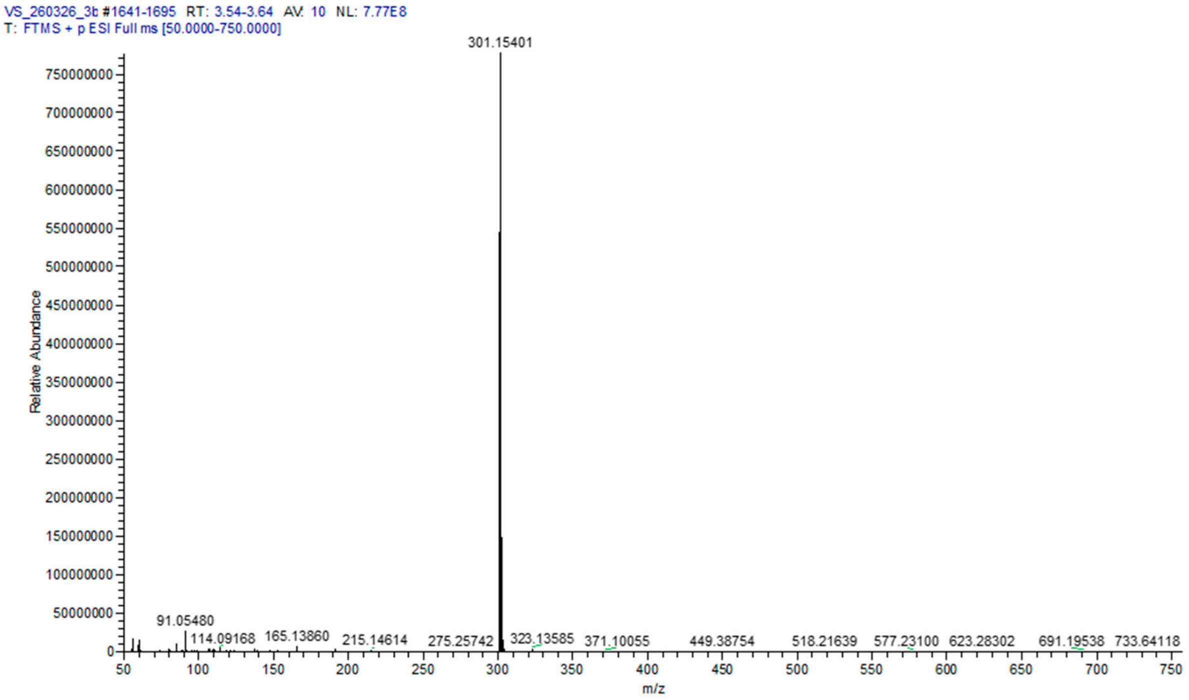


**Figure 20S**. HRMS of **3c.**

($R$,$E$)-4-methoxy-$N'$-2-methyl-5-(prop-1-en-2-yl)cyclohex-2-en-1-ylidene]benzohydrazide, **3d**

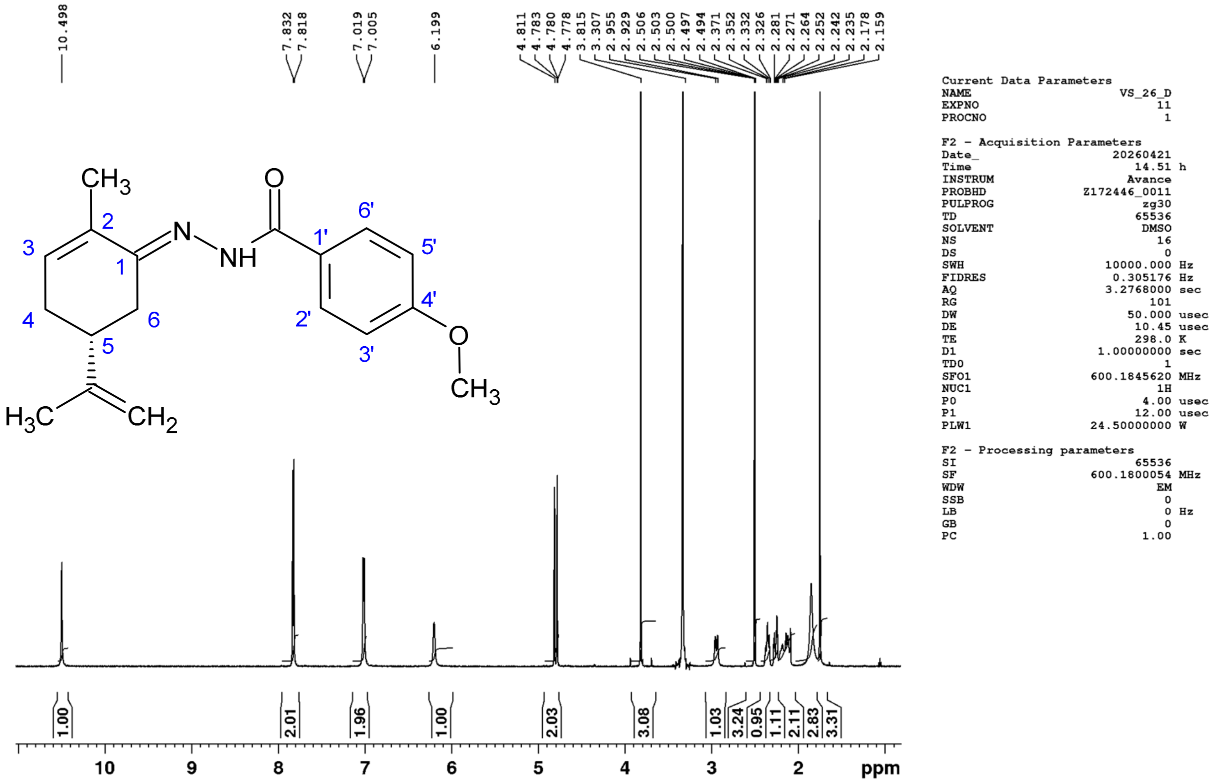


**Figure S21.** $^1$H NMR spectrum of **3d** in DMSO-$d_6$.

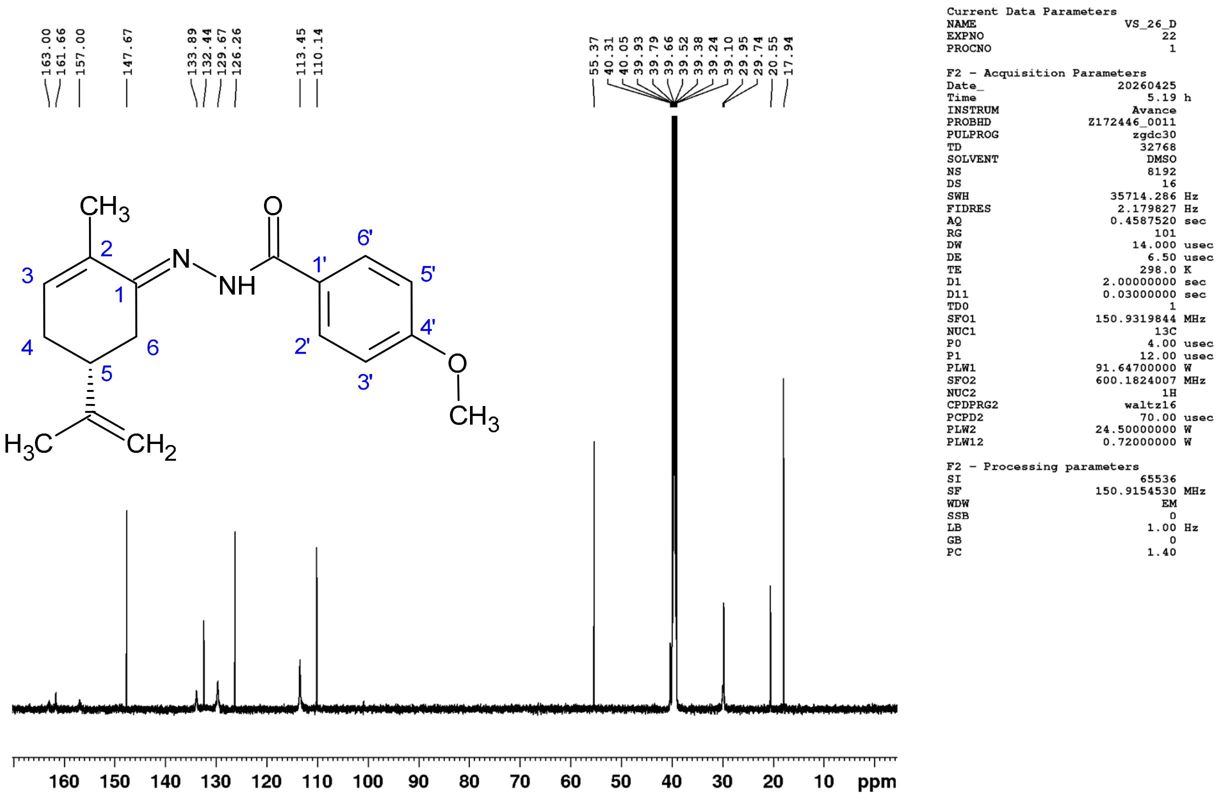


**Figure S22.** $^{13}$C NMR spectrum of **3d** in DMSO-$d_6$.

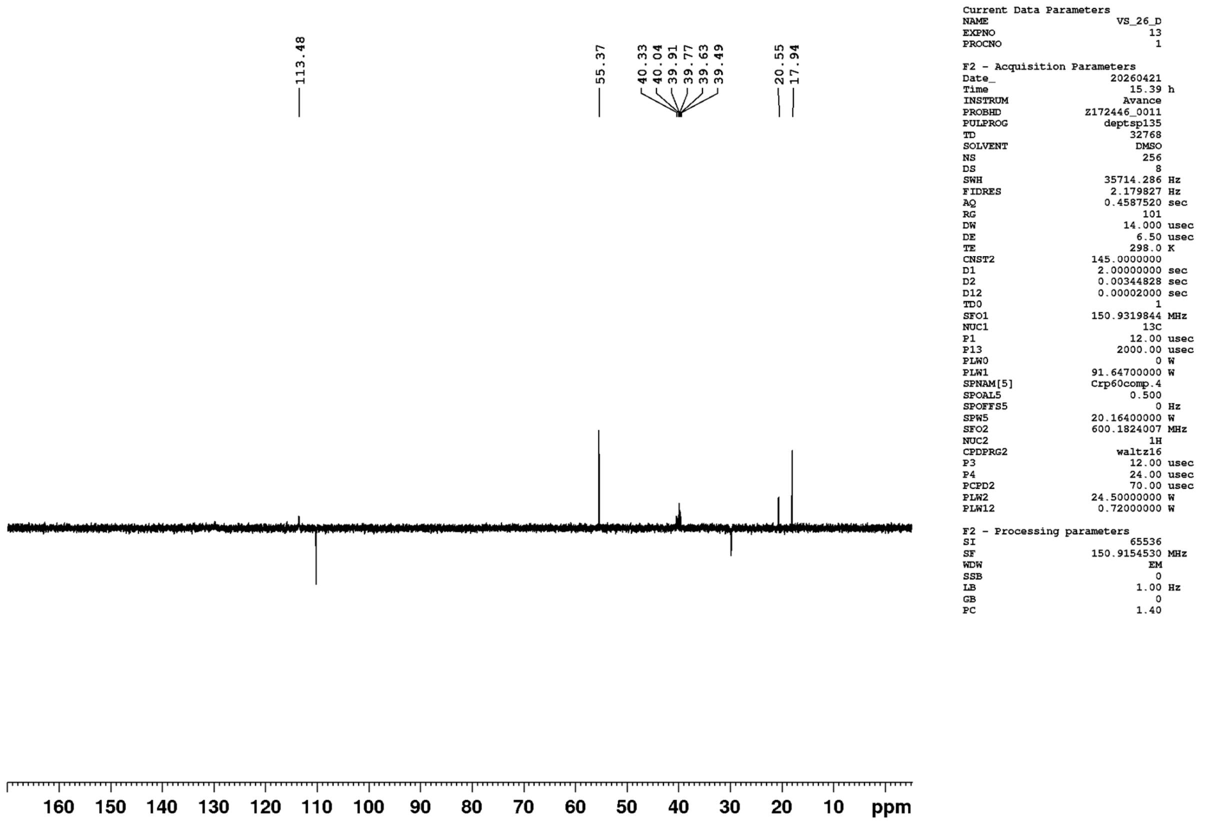


**Figure S23.** DEPT-135 NMR spectrum of **3d** in DMSO-$d_6$.

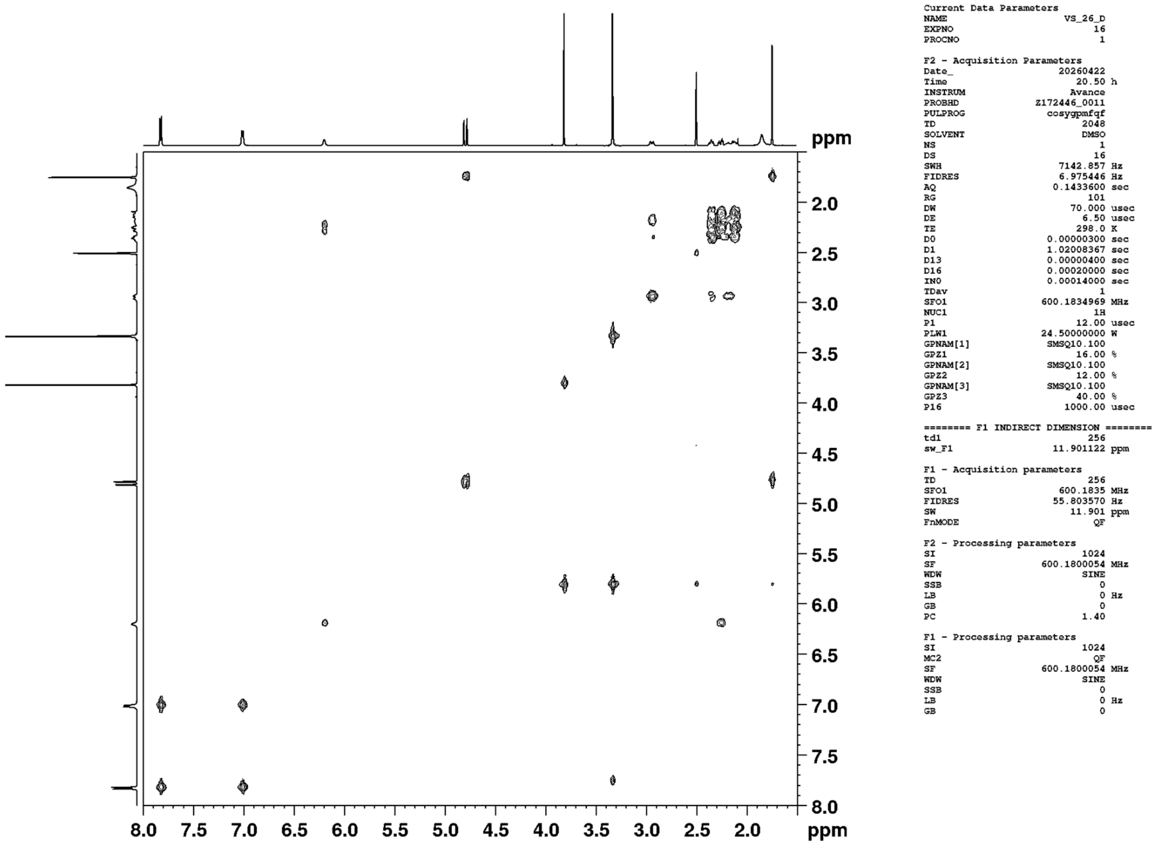


**Figure S24.** 2D COSY NMR spectrum of **3d** in DMSO-$d_6$.

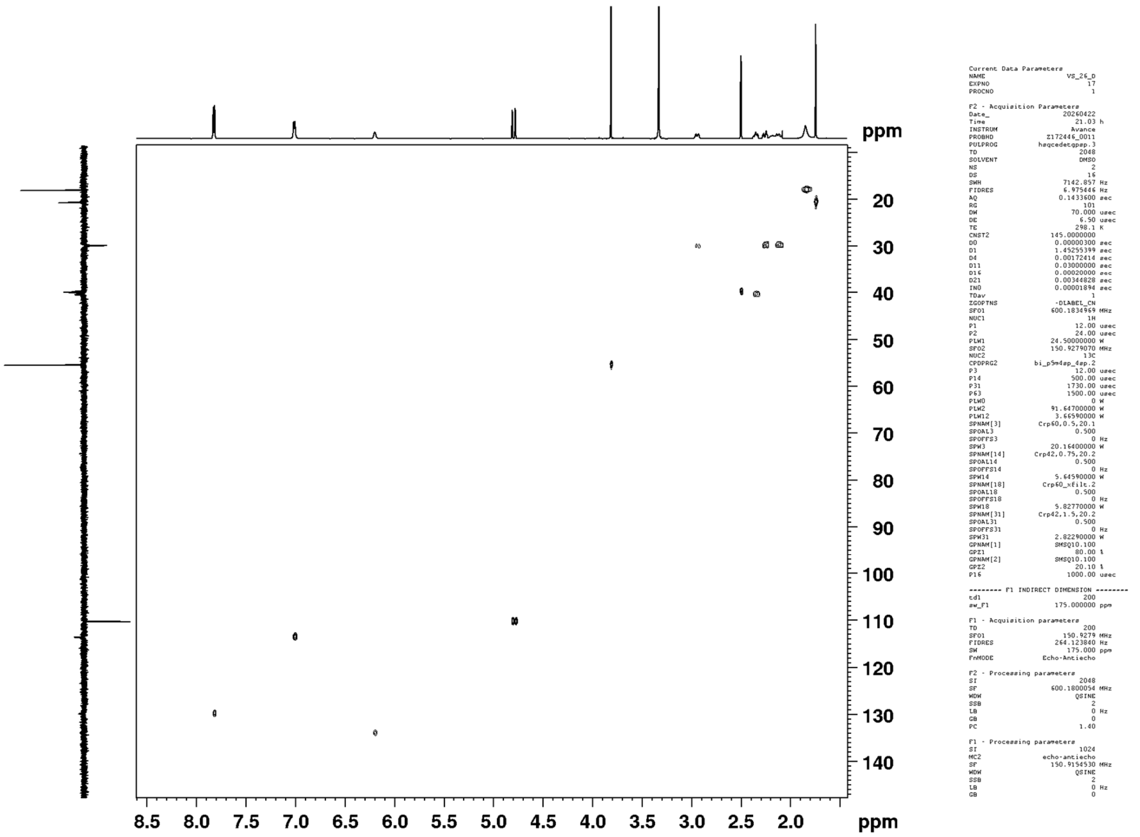


**Figure S25.** 2D HSQC NMR spectrum of **3d** in DMSO-$d_6$.

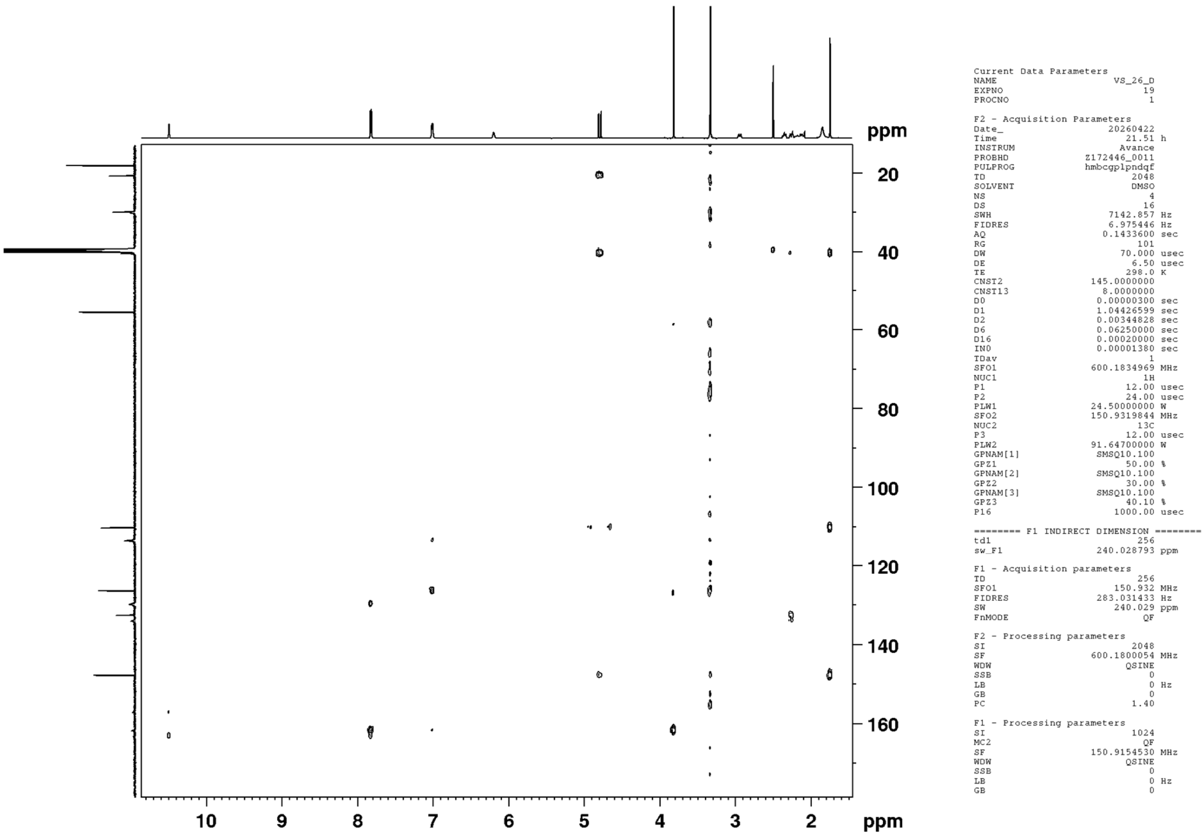


**Figure S26.** 2D HMBC NMR spectrum of **3d** in DMSO-$d_6$.

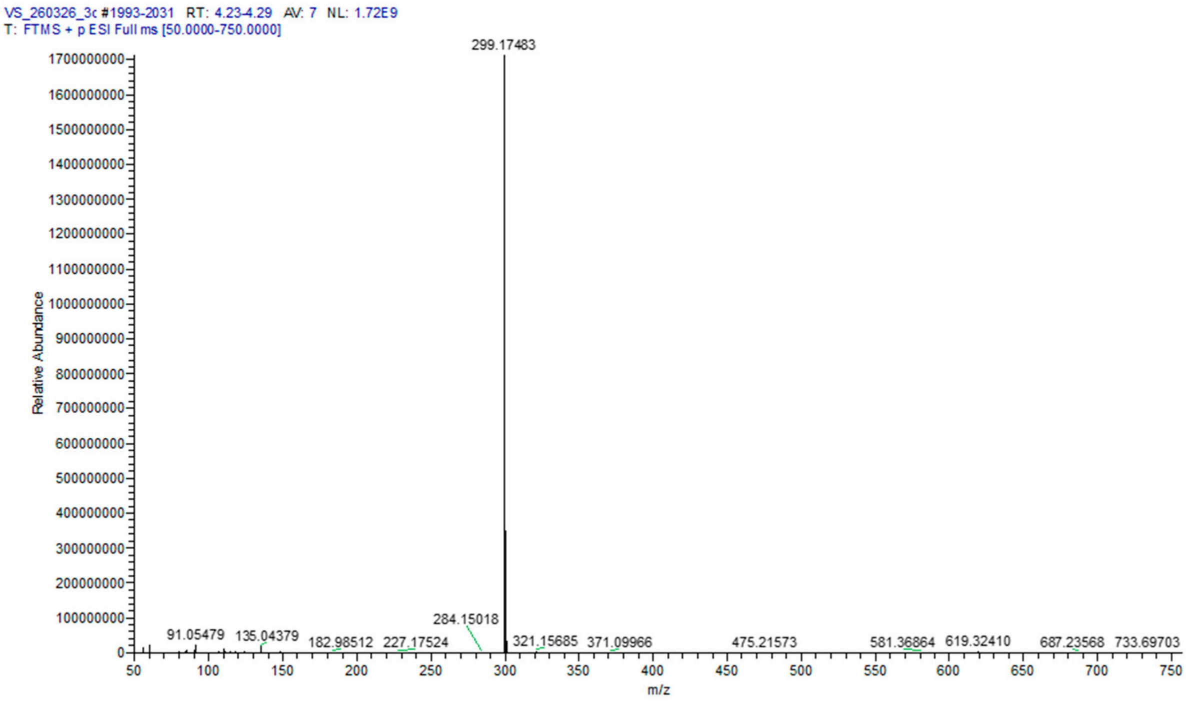


**Figure S27**. HRMS of **3d.**

4. (*R*,*E*)-2-methoxy-*N'*-2-methyl-5-(prop-1-en-2-yl)cyclohex-2-en-1-ylidene]benzohydrazide, **3e**

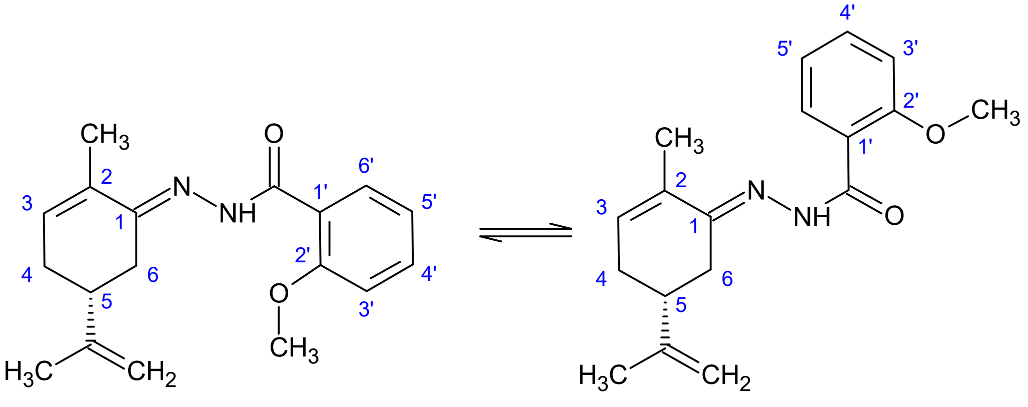


*Syn* *Anti*

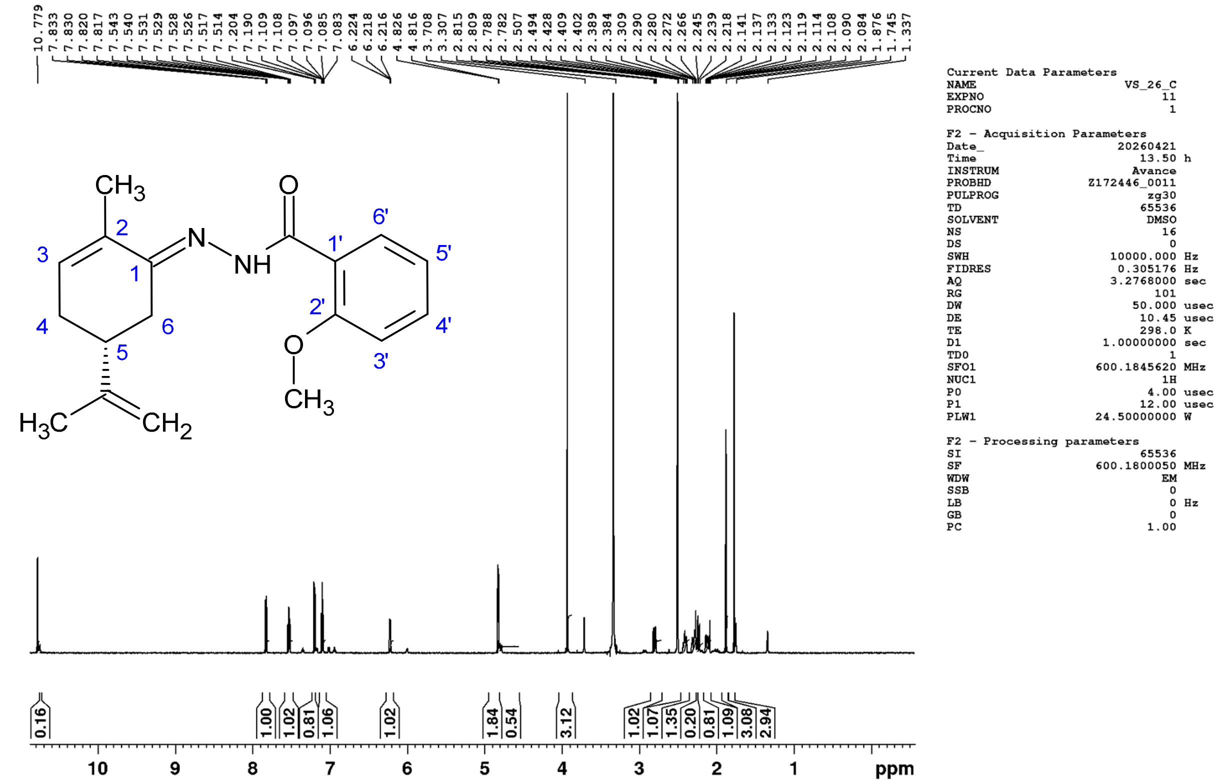


**Figure S28.** $^{1}H$ NMR spectrum of **3e** in DMSO-$d_6$.

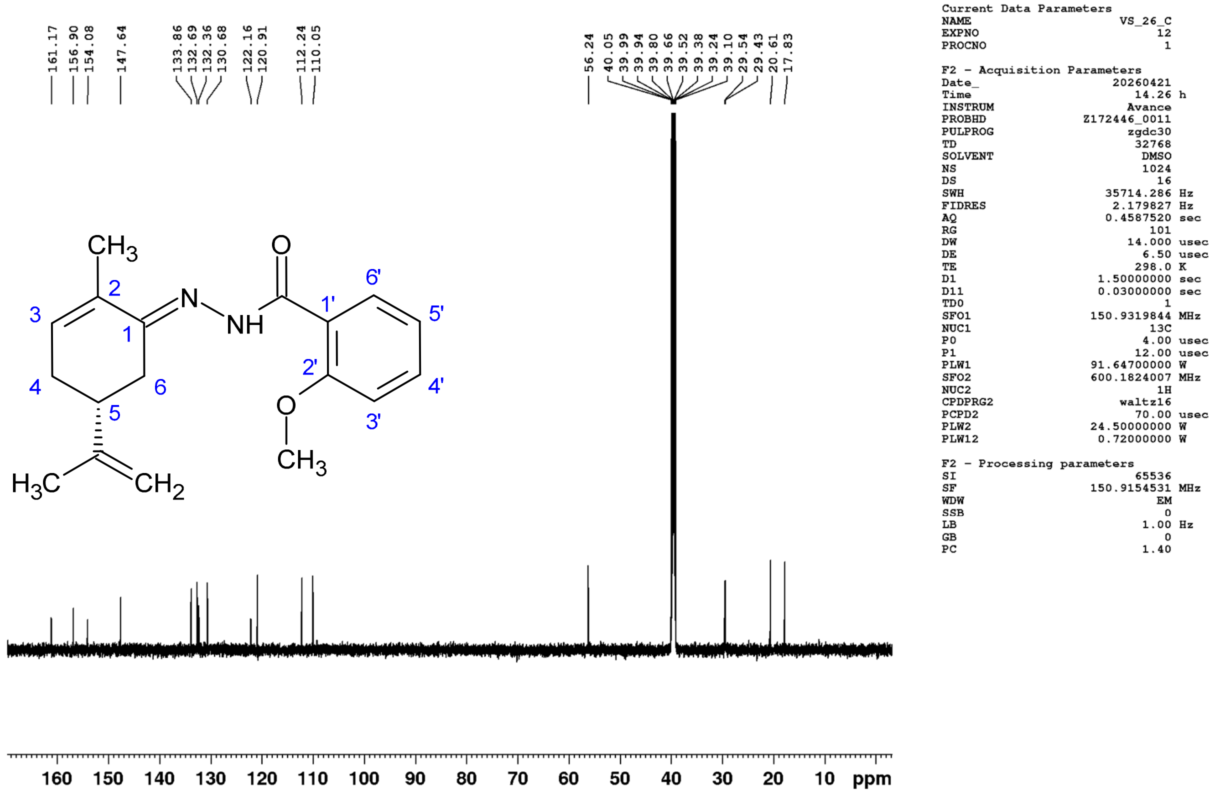


**Figure S29.** $^{13}$C NMR spectrum of **3e** in DMSO-$d_6$.

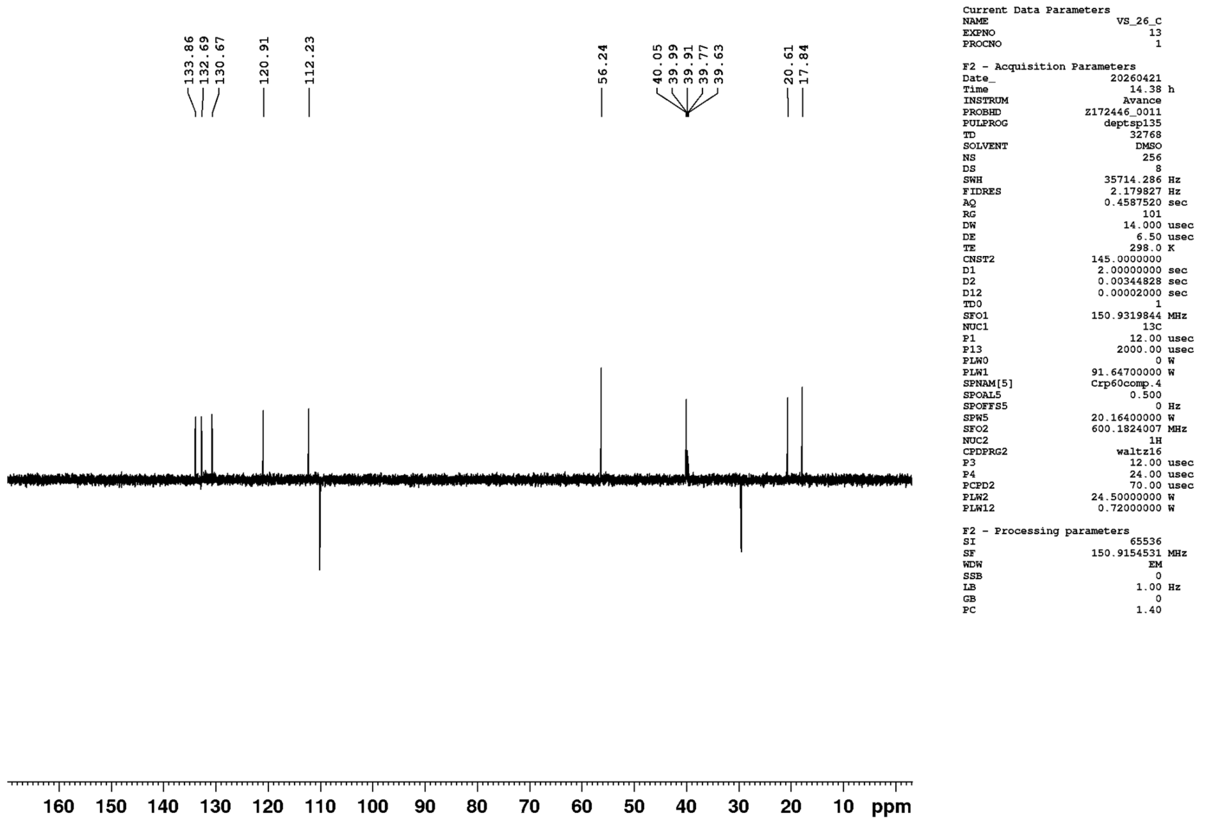


**Figure 30S.** DEPT-135 NMR spectrum of **3e** in DMSO-$d_6$.

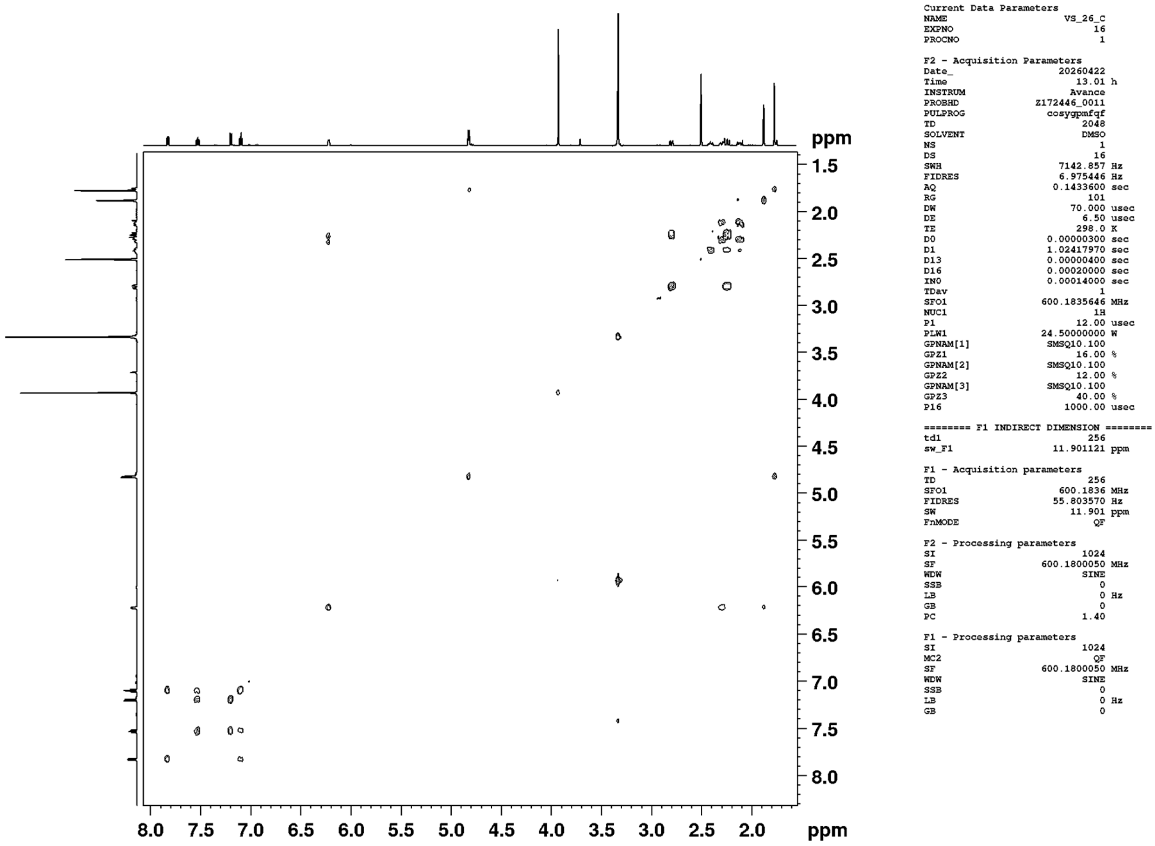


**Figure S31.** 2D COSY NMR spectrum of **3e** in DMSO-$d_6$.

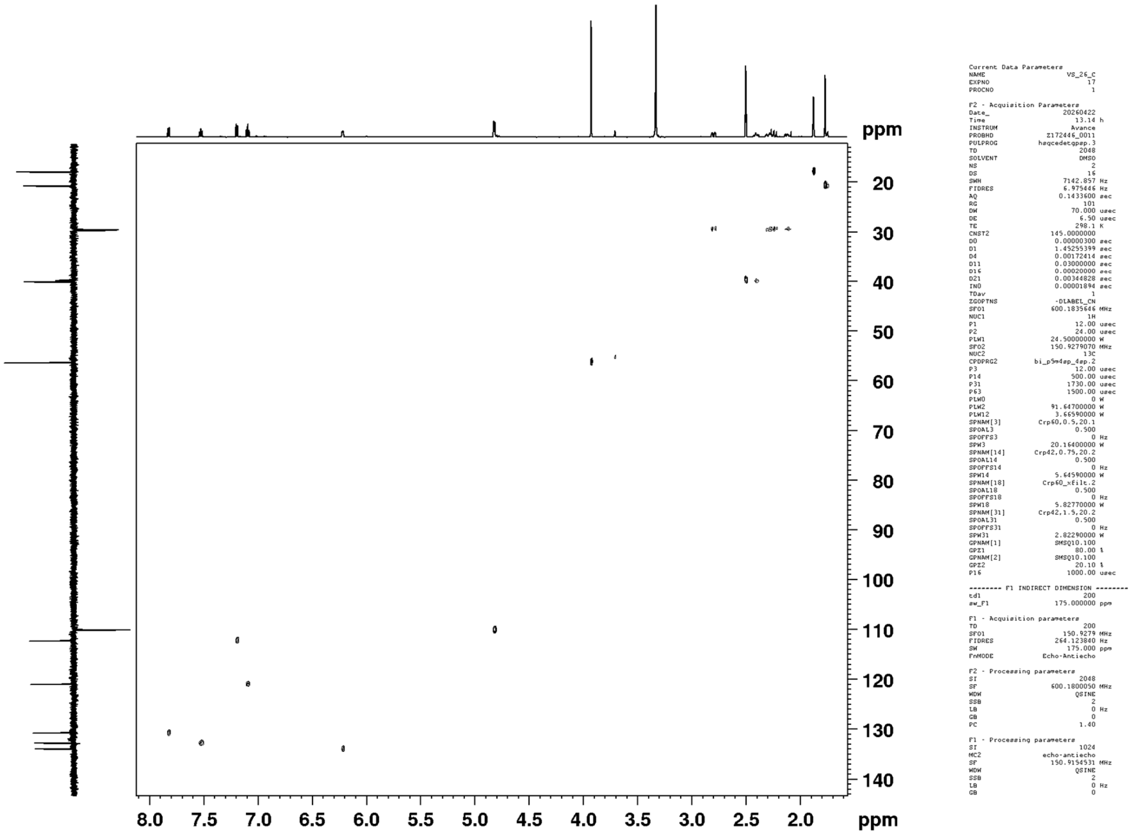


**Figure S32.** 2D HSQC NMR spectrum of **3e** in DMSO-$d_6$.

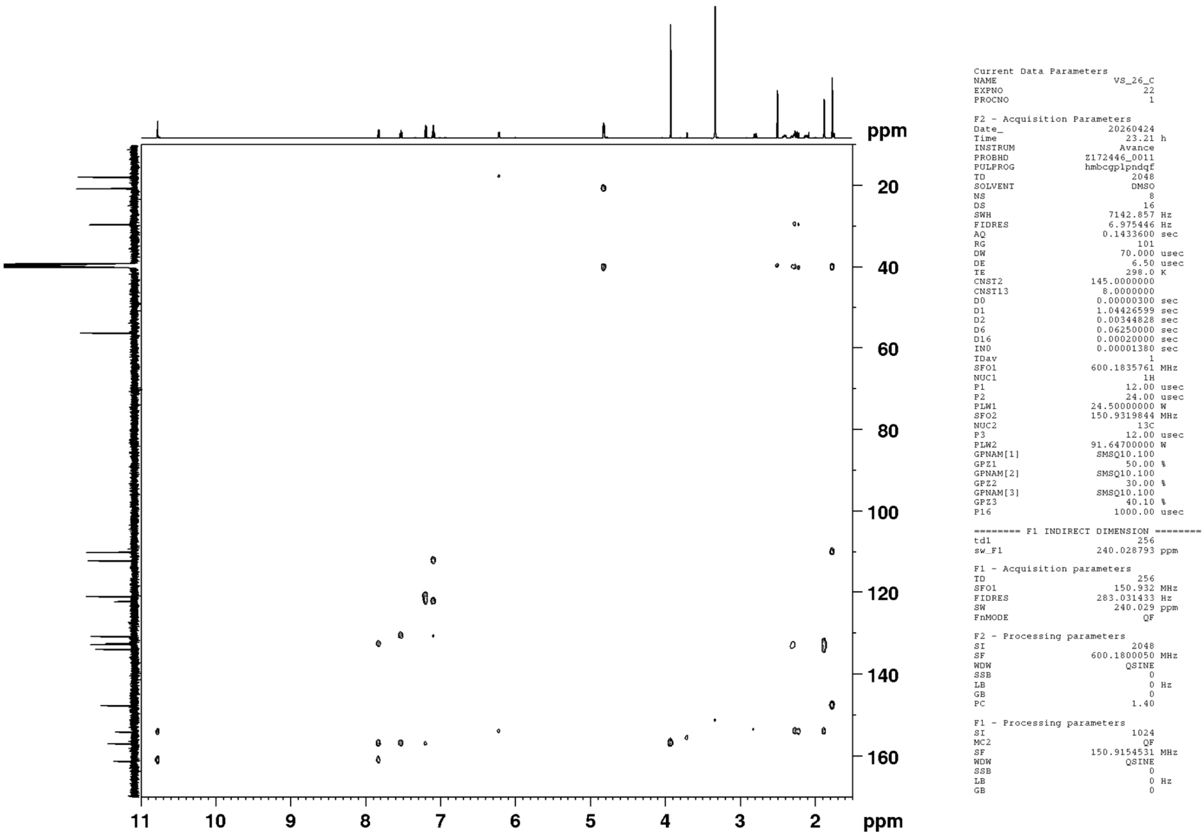


**Figure S33.** 2D HMBC NMR spectrum of **3e** in DMSO-$d_6$.

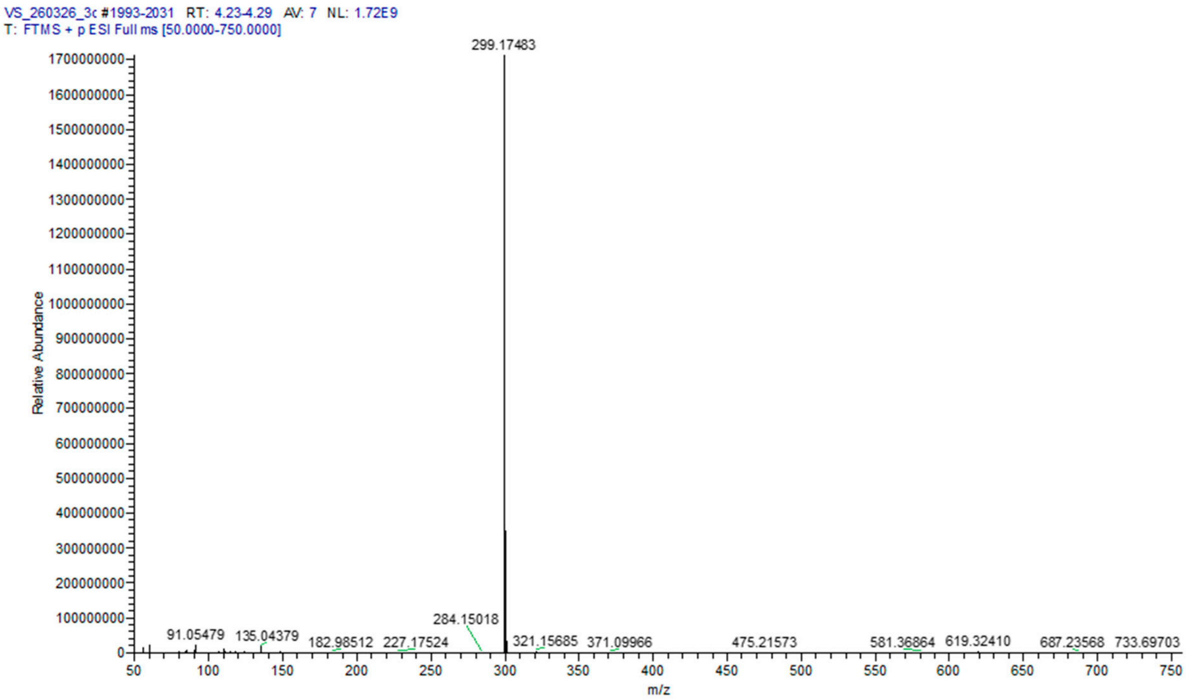


**Figure S34**. HRMS of **3e.**

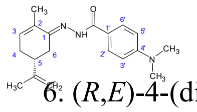

6. (*R*,*E*)-4-(dimethylamino)-*N'*-2-methyl-5-(prop-1-en-2-yl)cyclohex-2-en-1-ylidene] benzohydrazide, **3f**

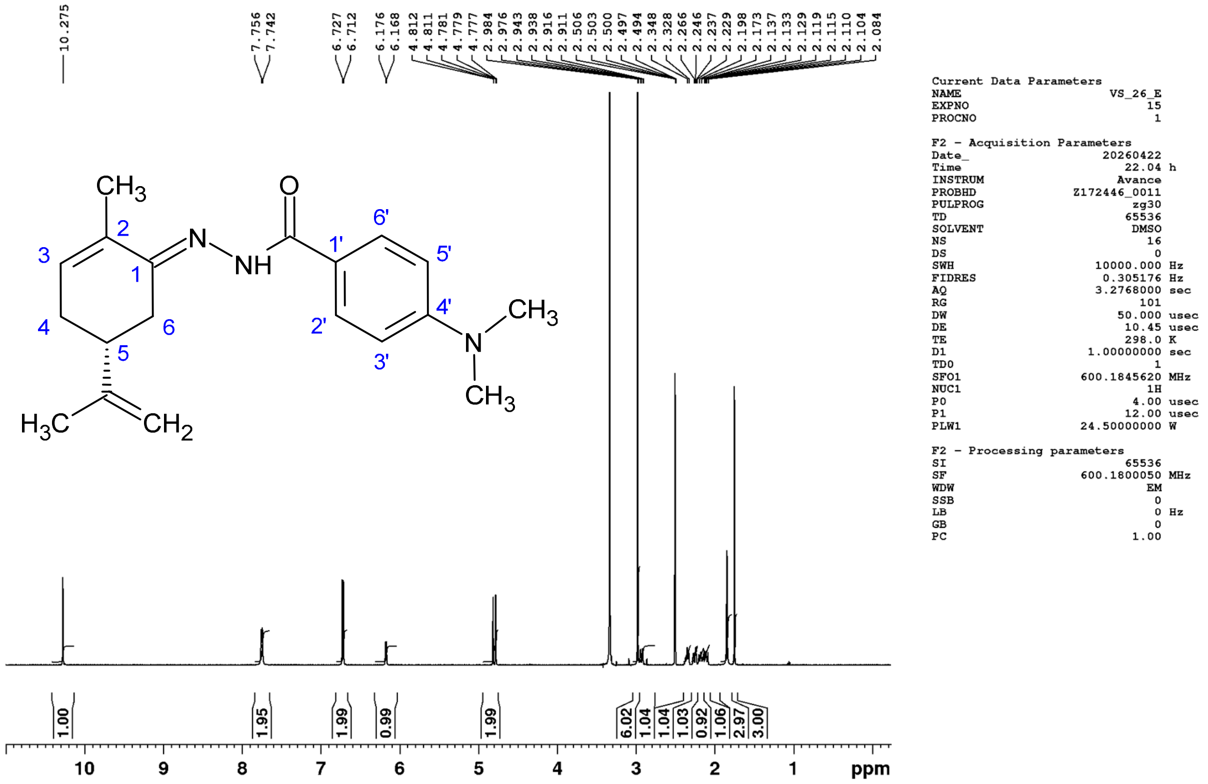


**Figure S35.** $^{1}H$ NMR spectrum of **3f** in DMSO-$d_6$.

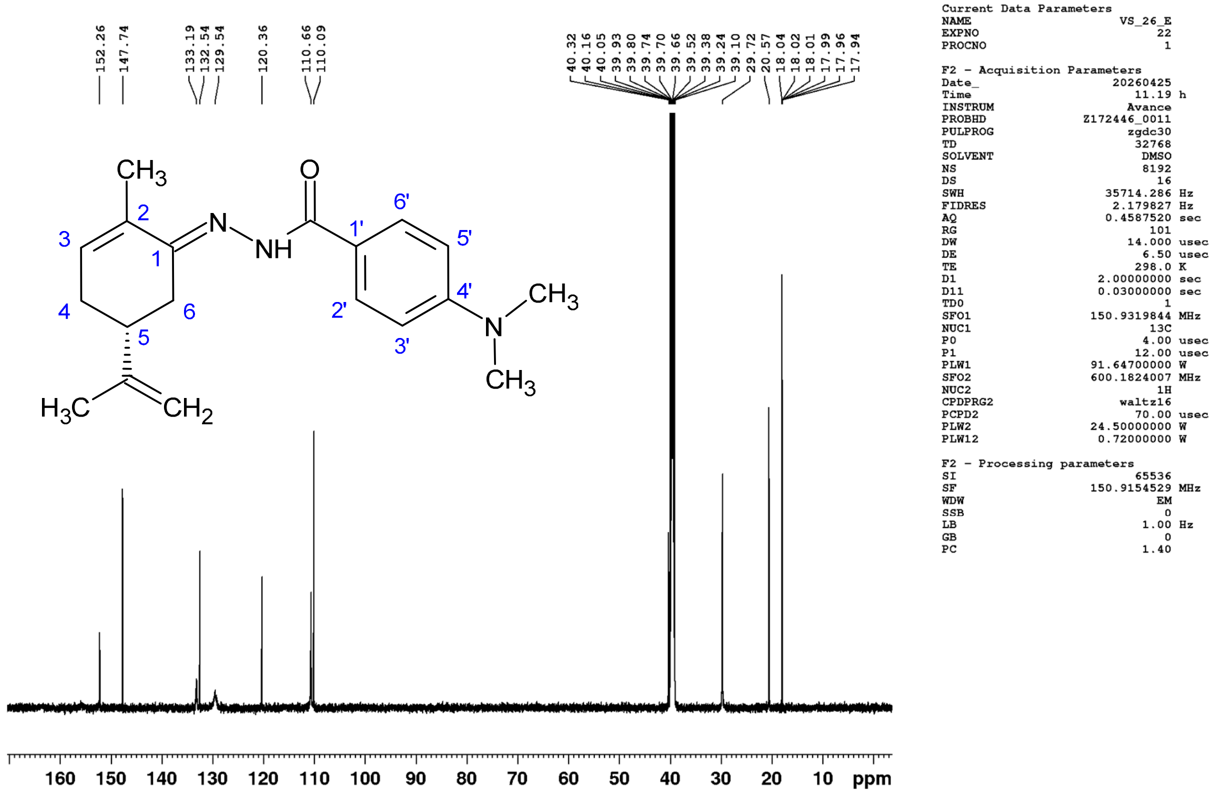


**Figure S36.** $^{13}$C NMR spectrum of **3f** in DMSO-$d_6$.

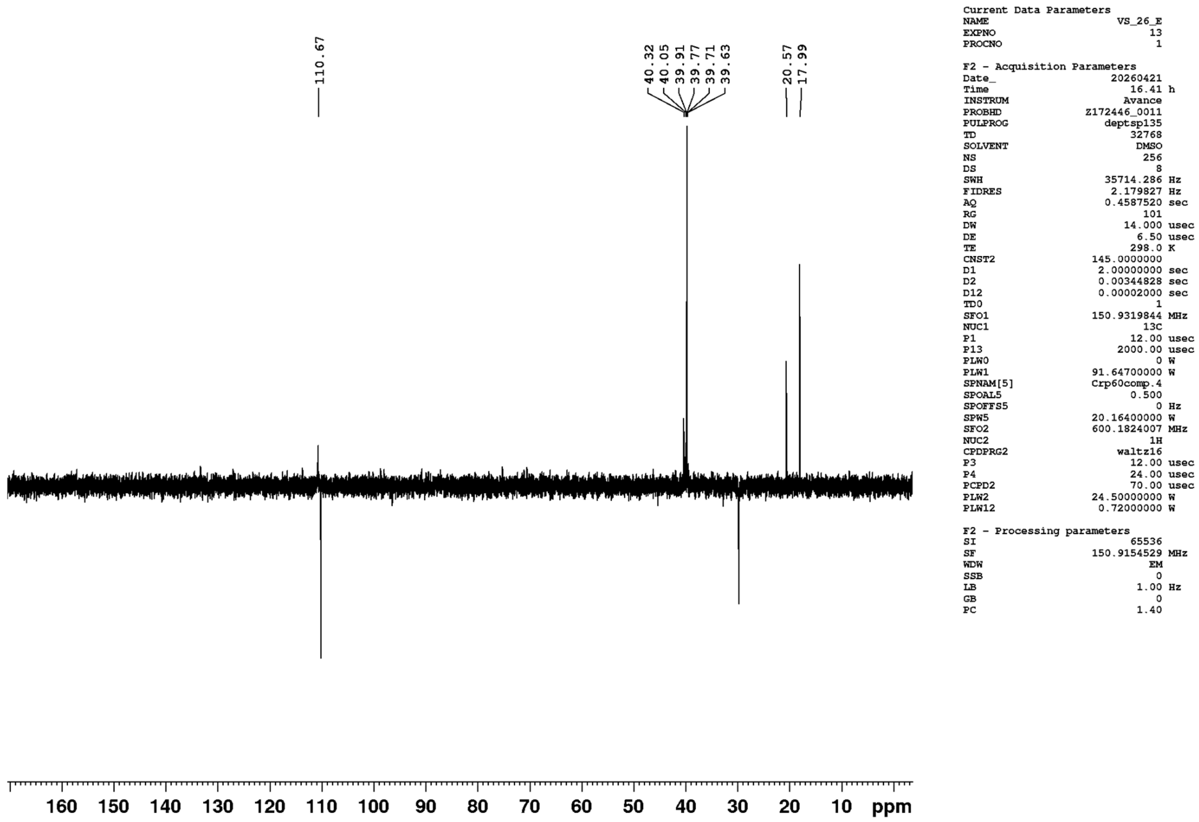


**Figure S37.** DEPT-135 NMR spectrum of **3f** in DMSO-$d_6$.

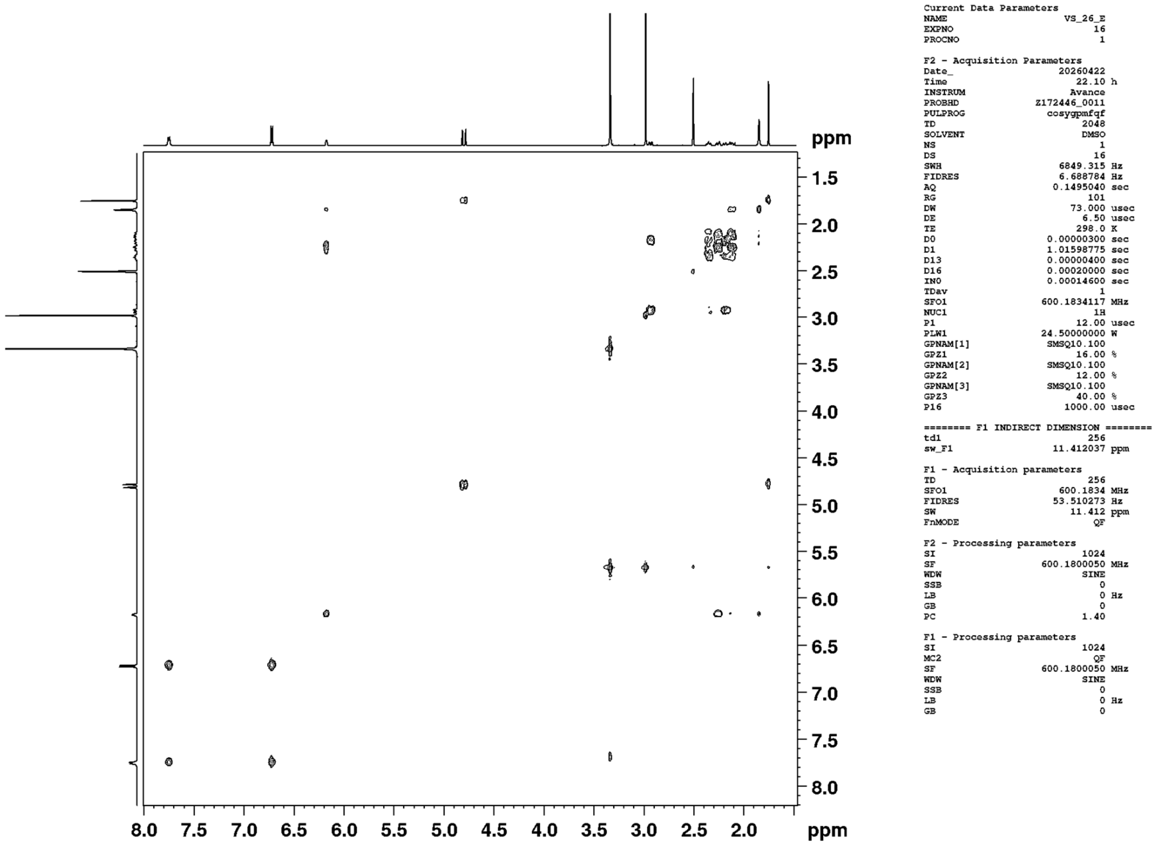


**Figure S38.** 2D COSY NMR spectrum of **3f** in DMSO-$d_6$.

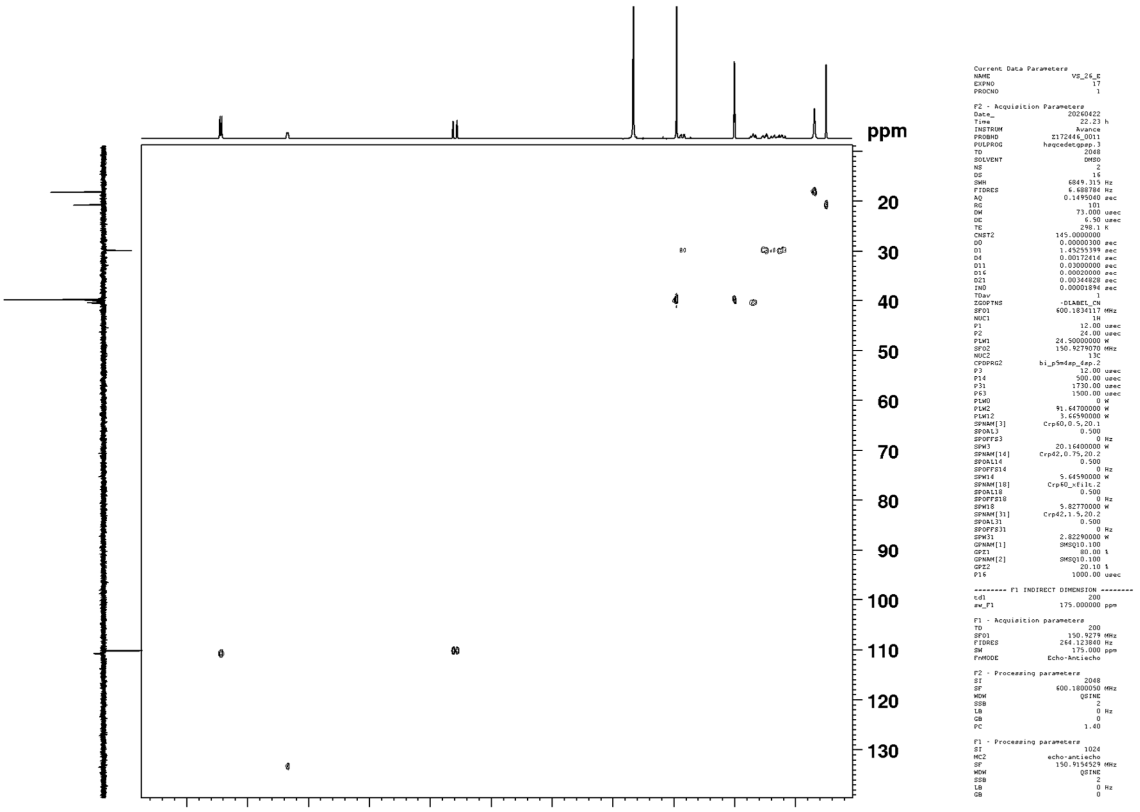


**Figure S39.** 2D HSQC NMR spectrum of **3f** in DMSO-$d_6$.

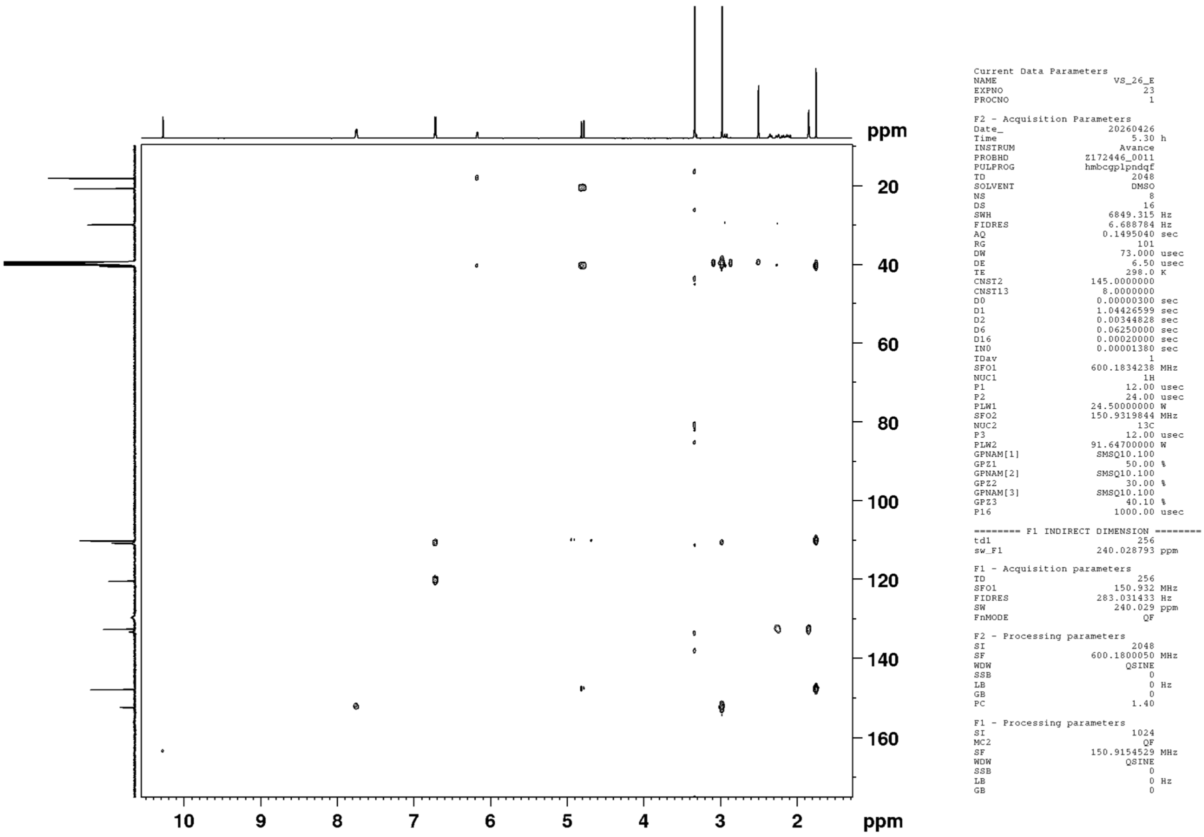


**Figure 40S.** 2D HMBC NMR spectrum of **3f** in DMSO-$d_6$.

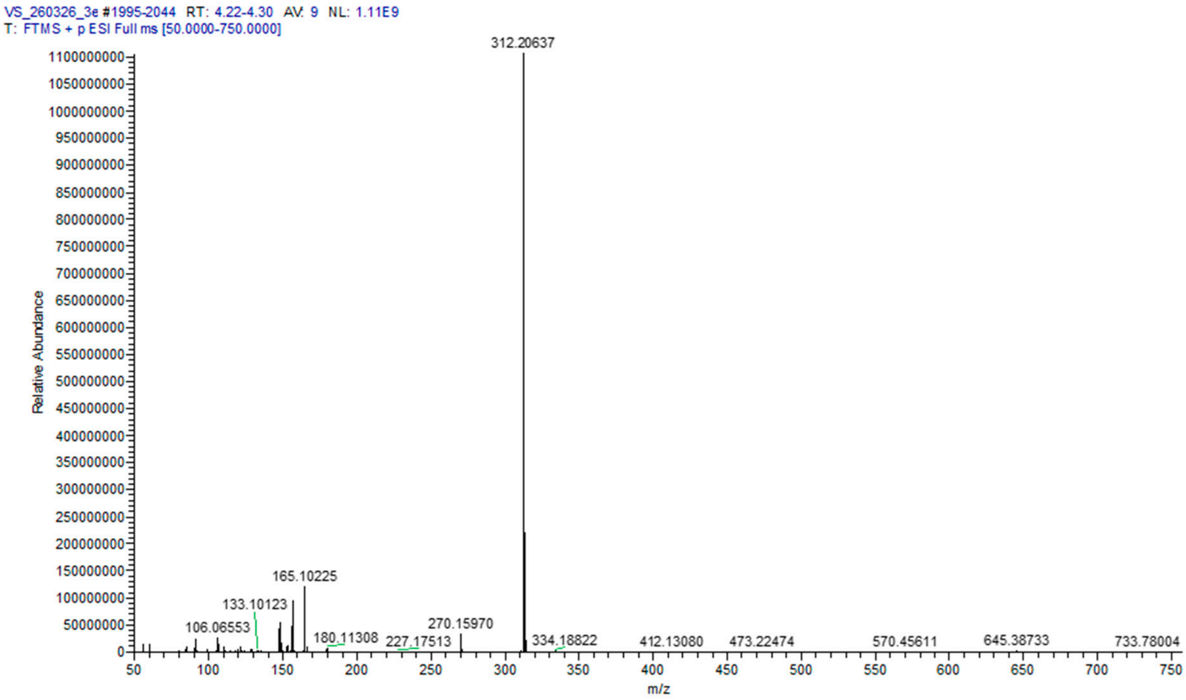


**Figure S41**. HRMS of **3f.**

7. ($R$,$E$)-2-(1$H$-indol-3-yl)-$N'$-[2-methyl-5-(prop-1-en-2-yl)cyclohex-2-en-1-ylidene] acetohydrazide, **3g**

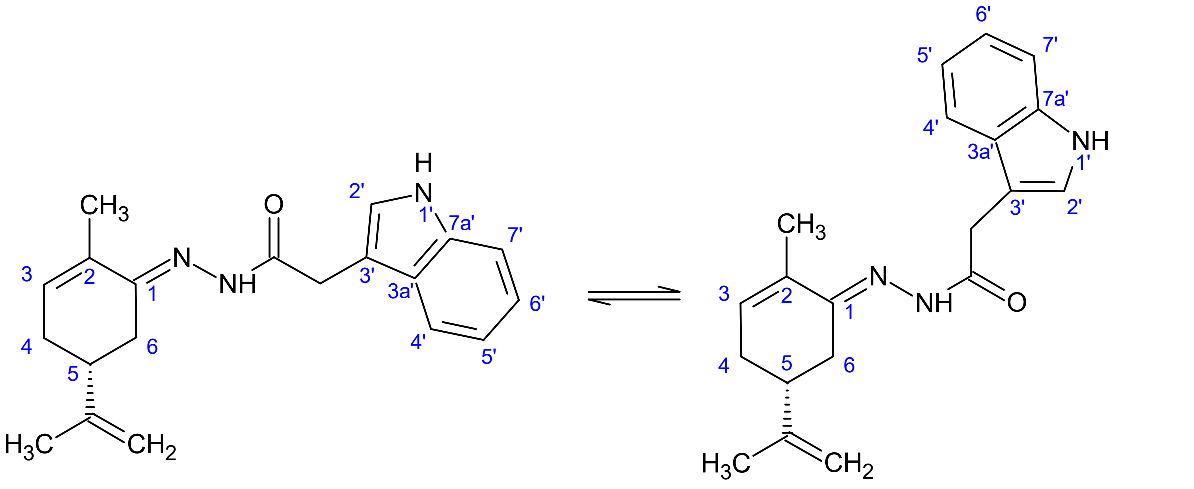


*Syn* *Anti*

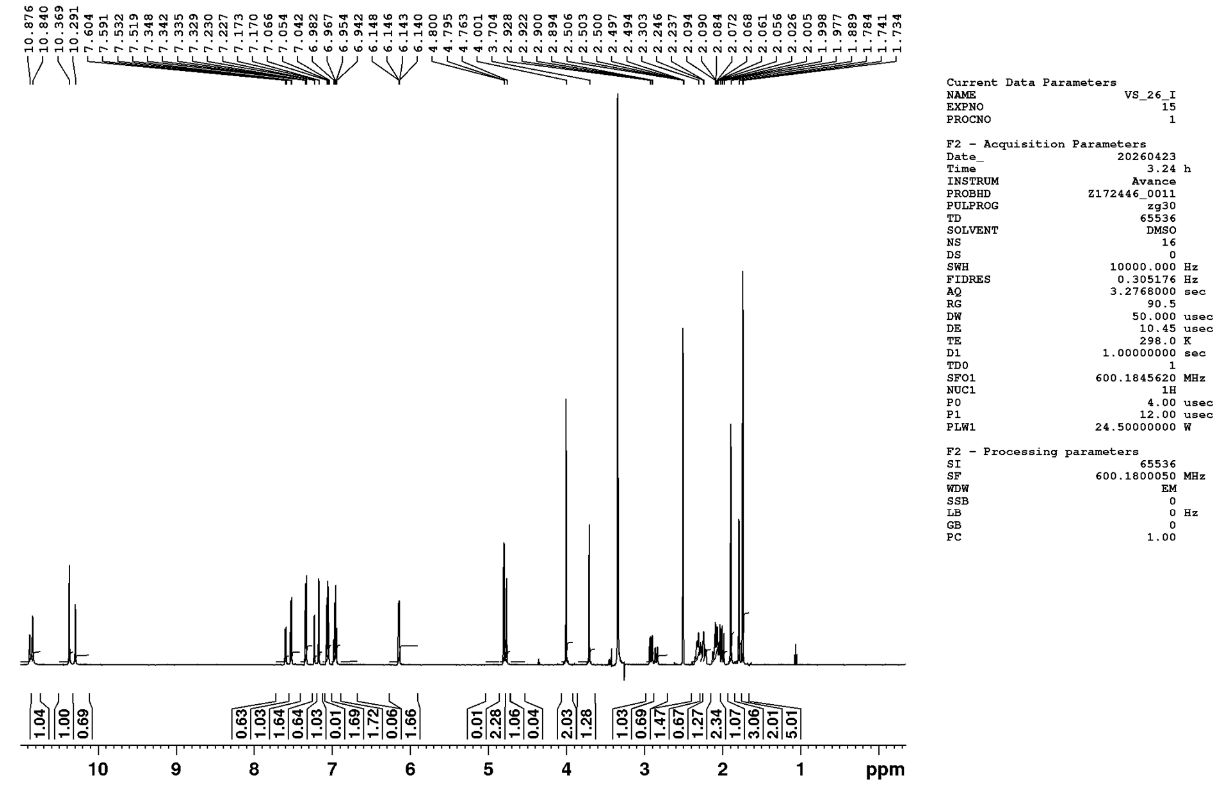


**Figure S42.** $^{1}$H NMR spectrum of **3g** in DMSO-$d_6$.

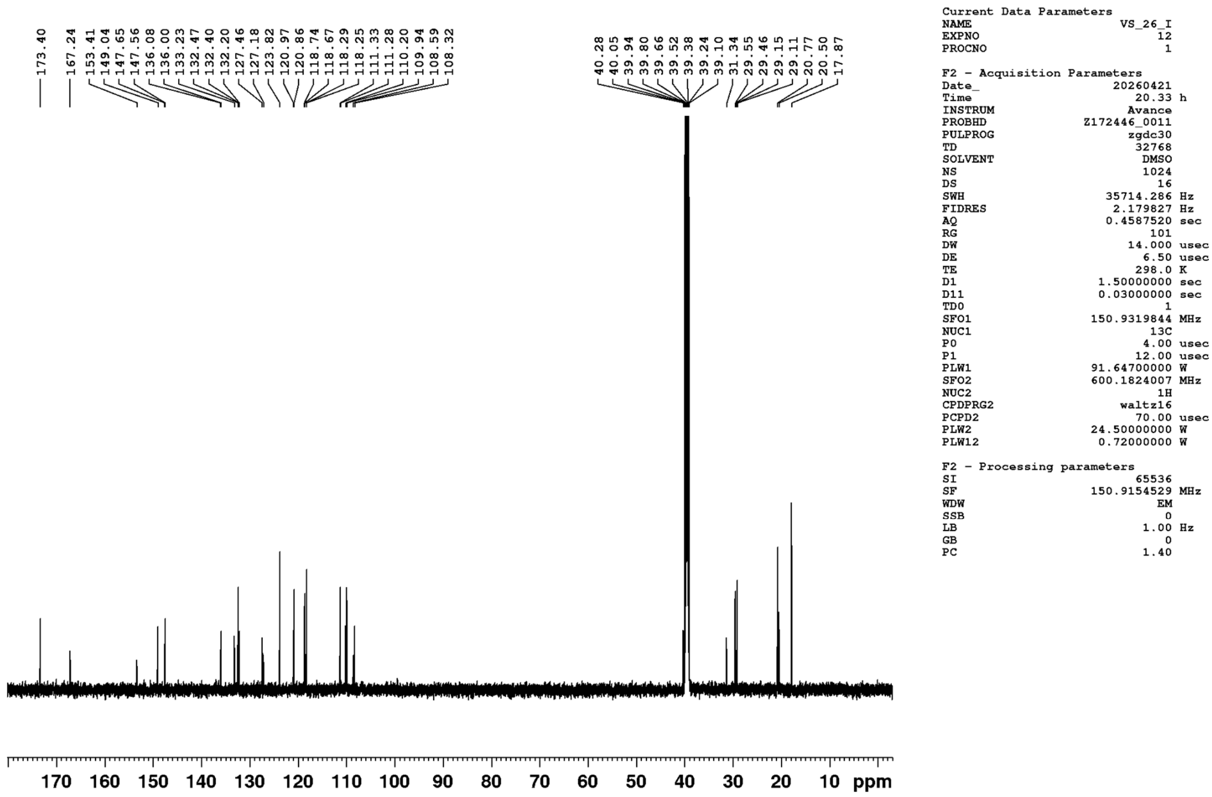


**Figure S43.** $^{13}C$ NMR spectrum of **3g** in DMSO-$d_6$.

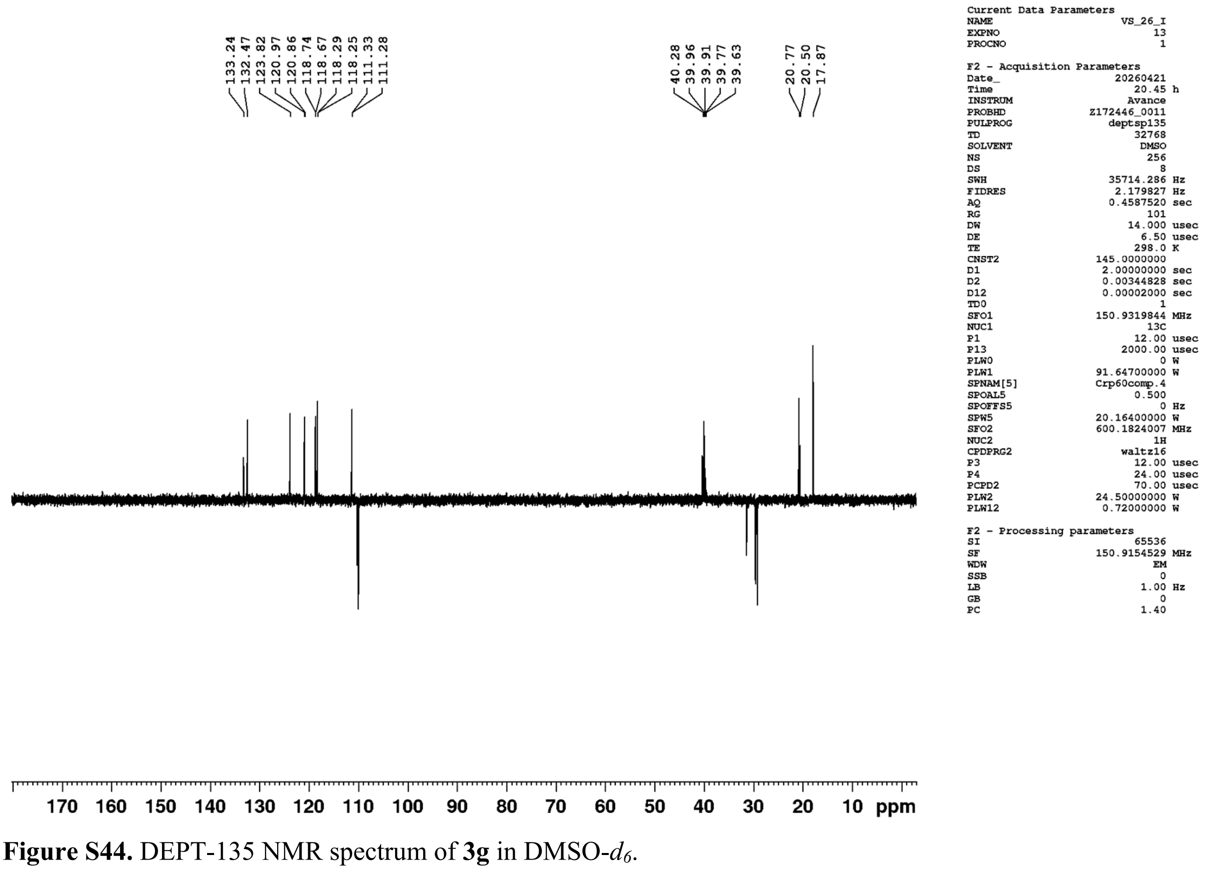


**Figure S44.** DEPT-135 NMR spectrum of **3g** in DMSO-$d_6$.

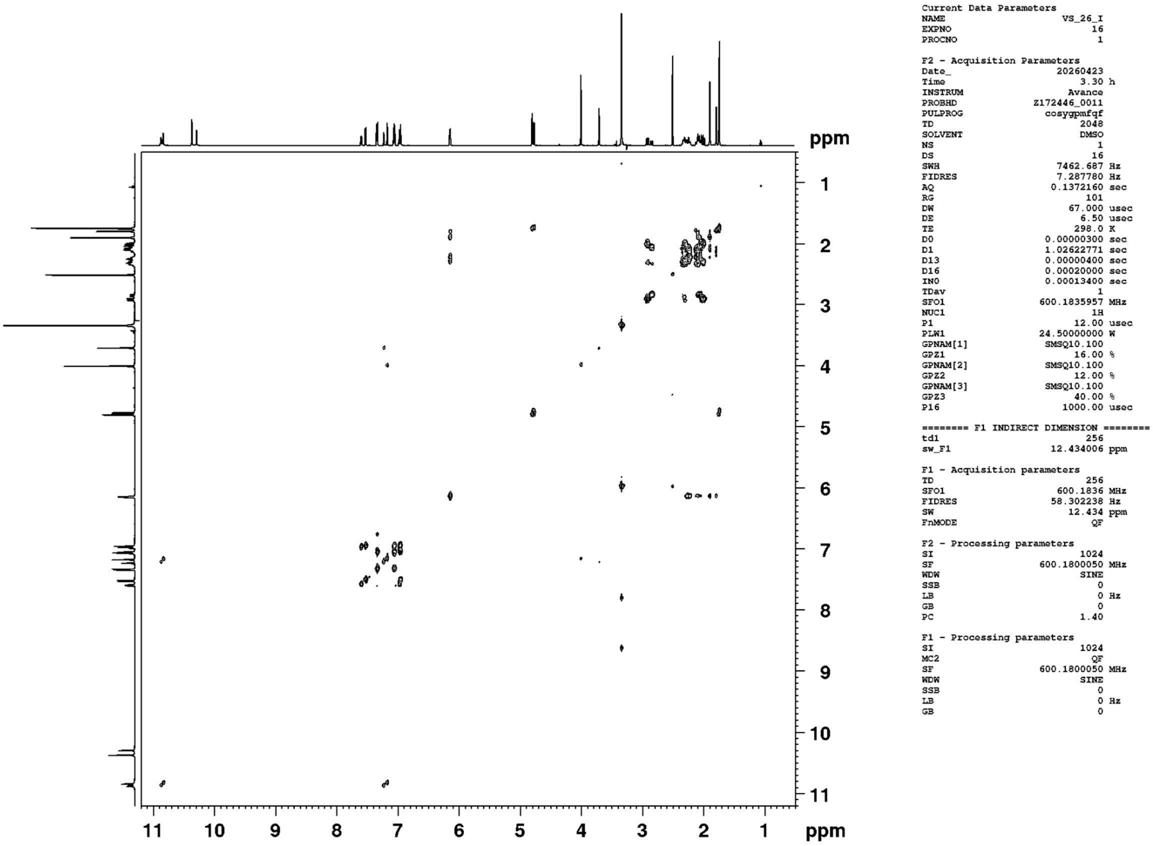


**Figure S45.** 2D COSY NMR spectrum of **3g** in DMSO-$d_6$.

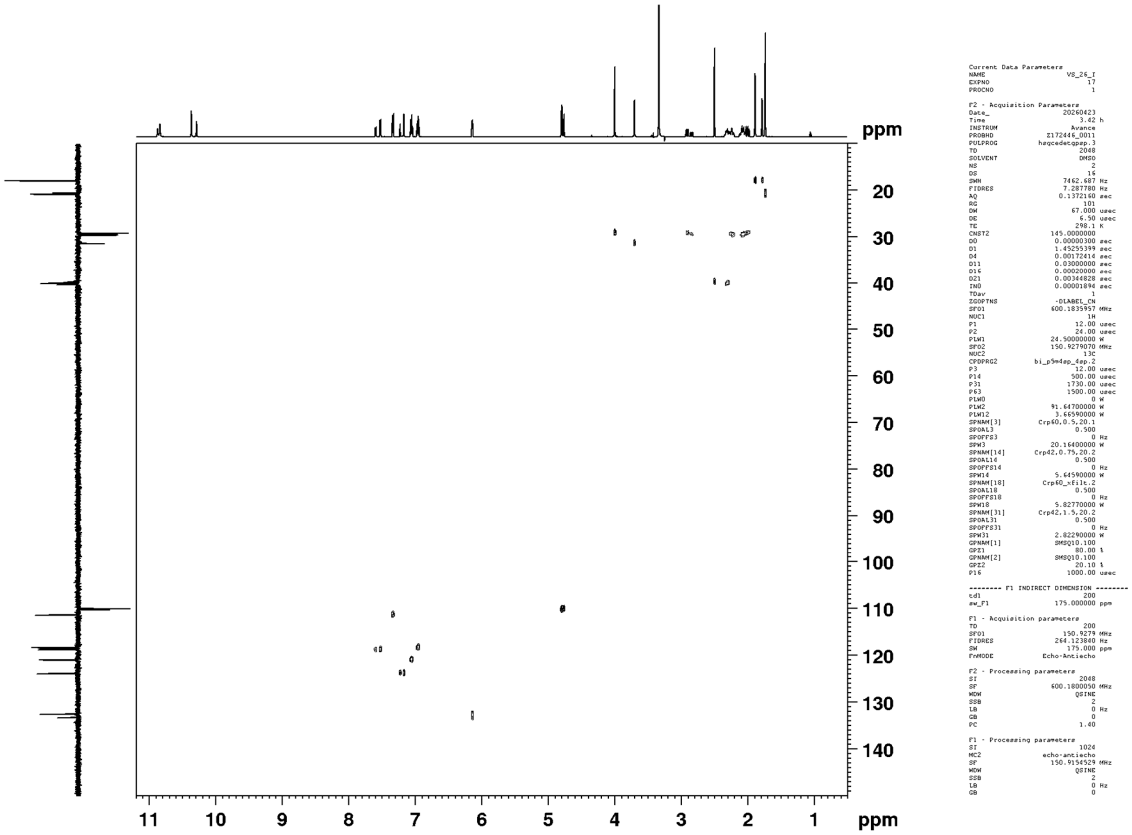


**Figure S46.** 2D HSQC NMR spectrum of **3g** in DMSO-$d_6$.

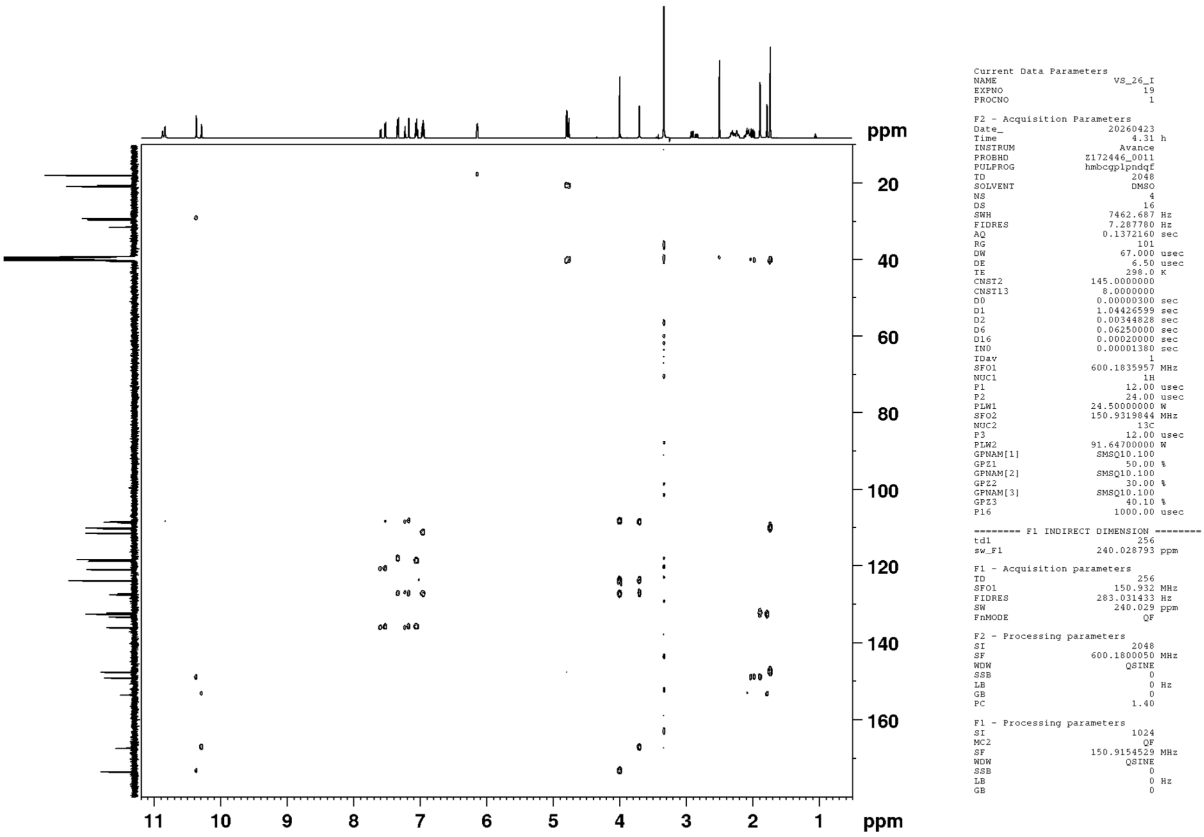


**Figure S47.** 2D HMBC NMR spectrum of **3g** in DMSO-$d_6$.

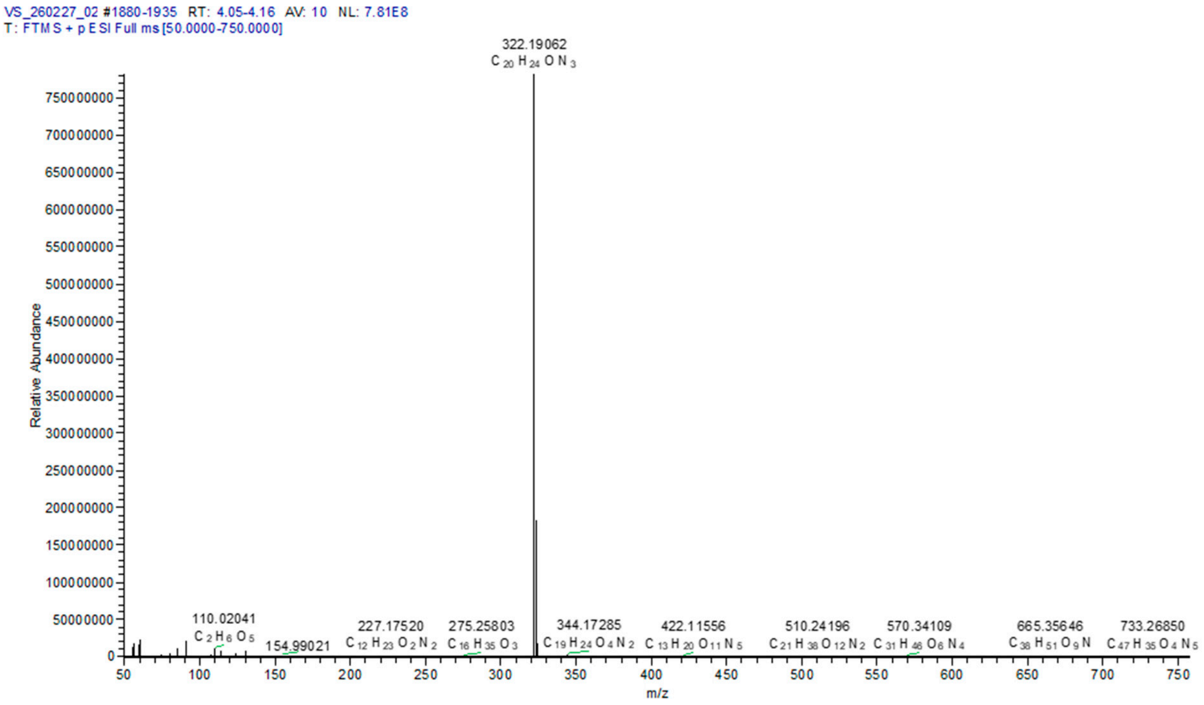


**Figure S48**. HRMS of **3g**.

8. (*R*,*E*)-*N*'-[2-methyl-5-(prop-1-en-2-yl)cyclohex-2-en-1-ylidene]-1*H*-indole-3-carbohydrazide, **3h**

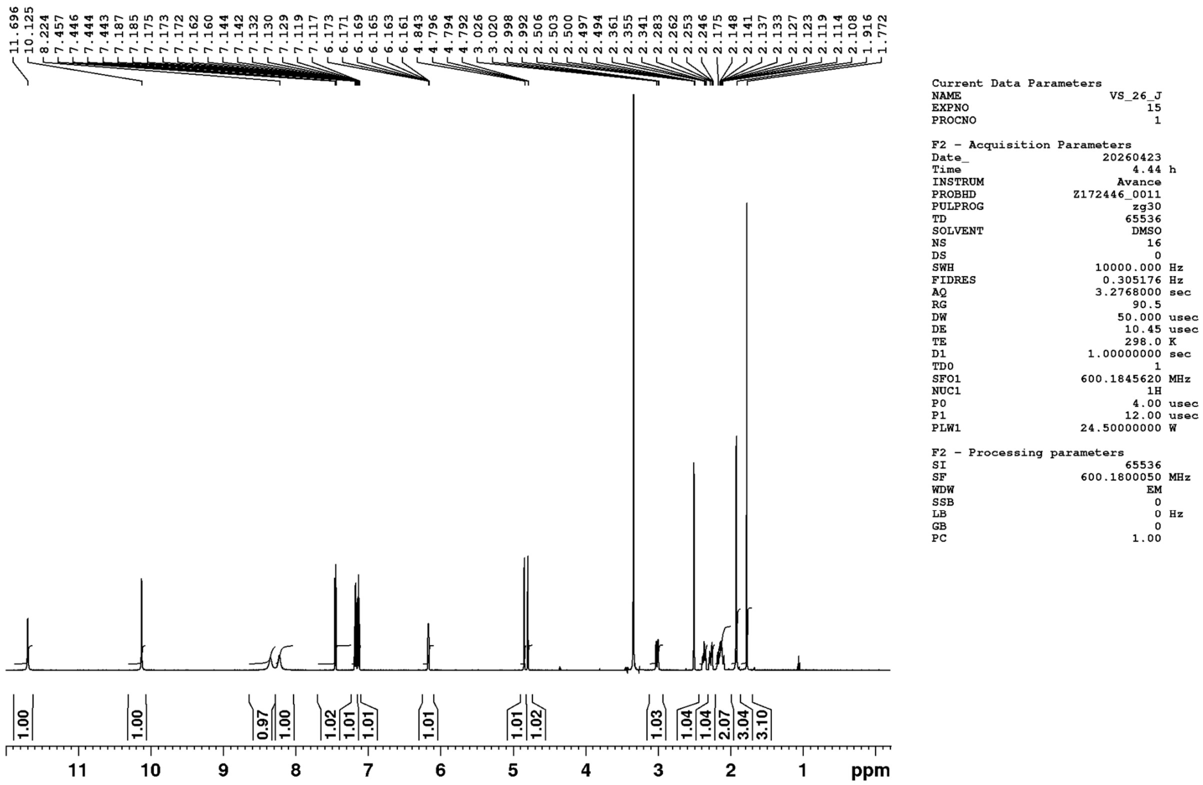


**Figure S49.** $^{1}$H NMR spectrum of **3h** in DMSO-$d_6$.

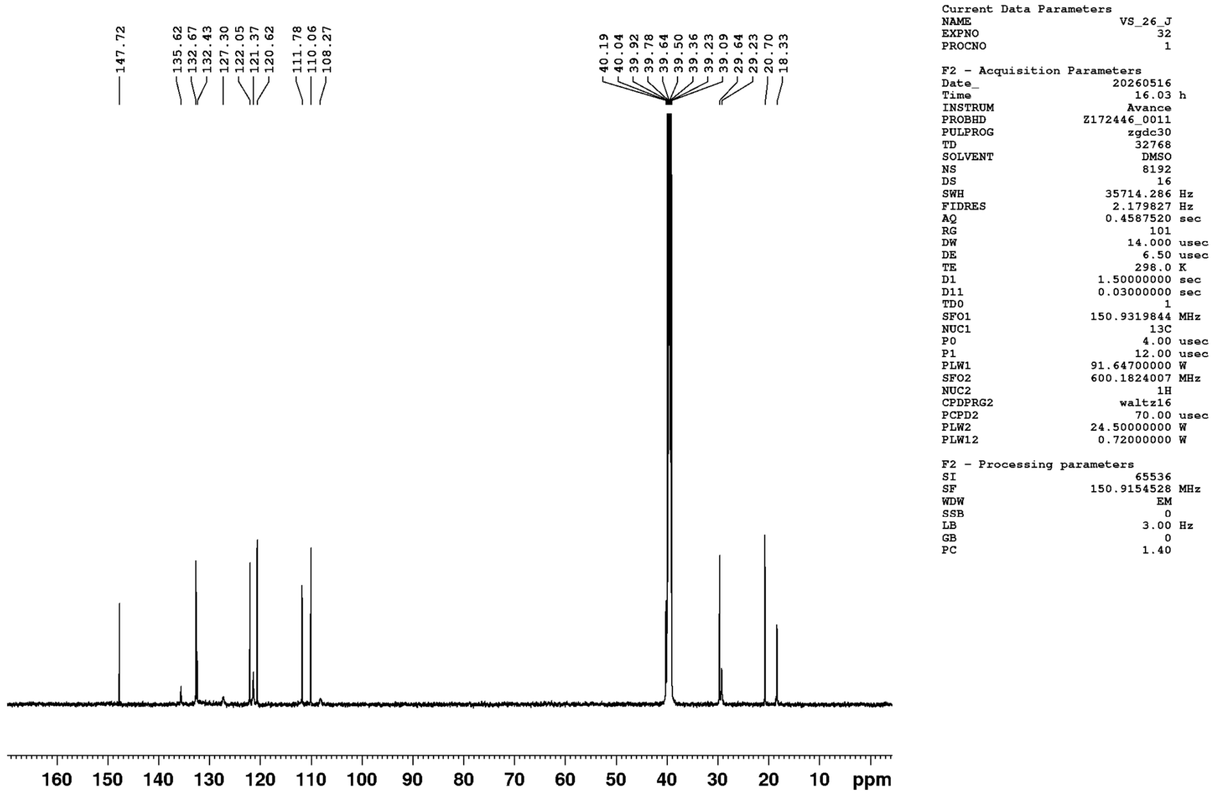


**Figure 50S.** $^{13}$C NMR spectrum of **3h** in DMSO-$d_6$.

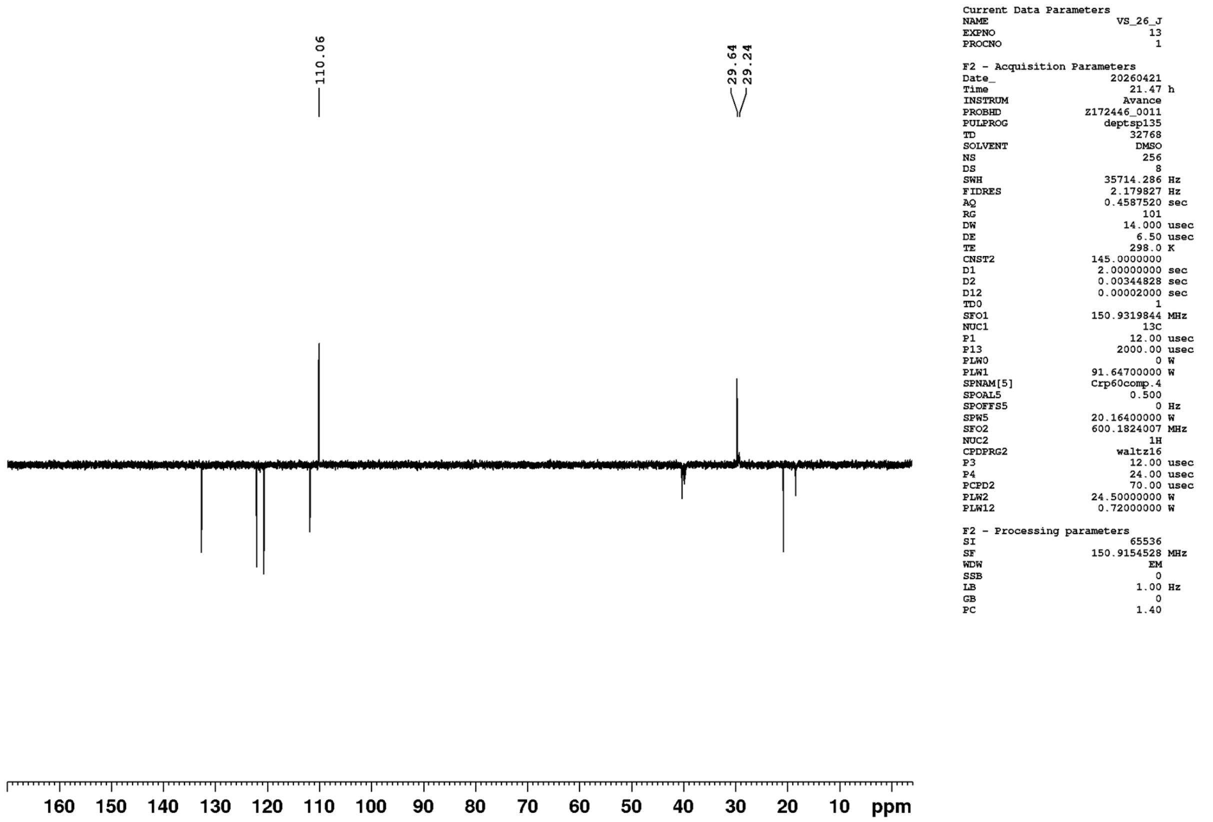


**Figure S51.** DEPT-135 NMR spectrum of **3h** in DMSO-$d_6$.

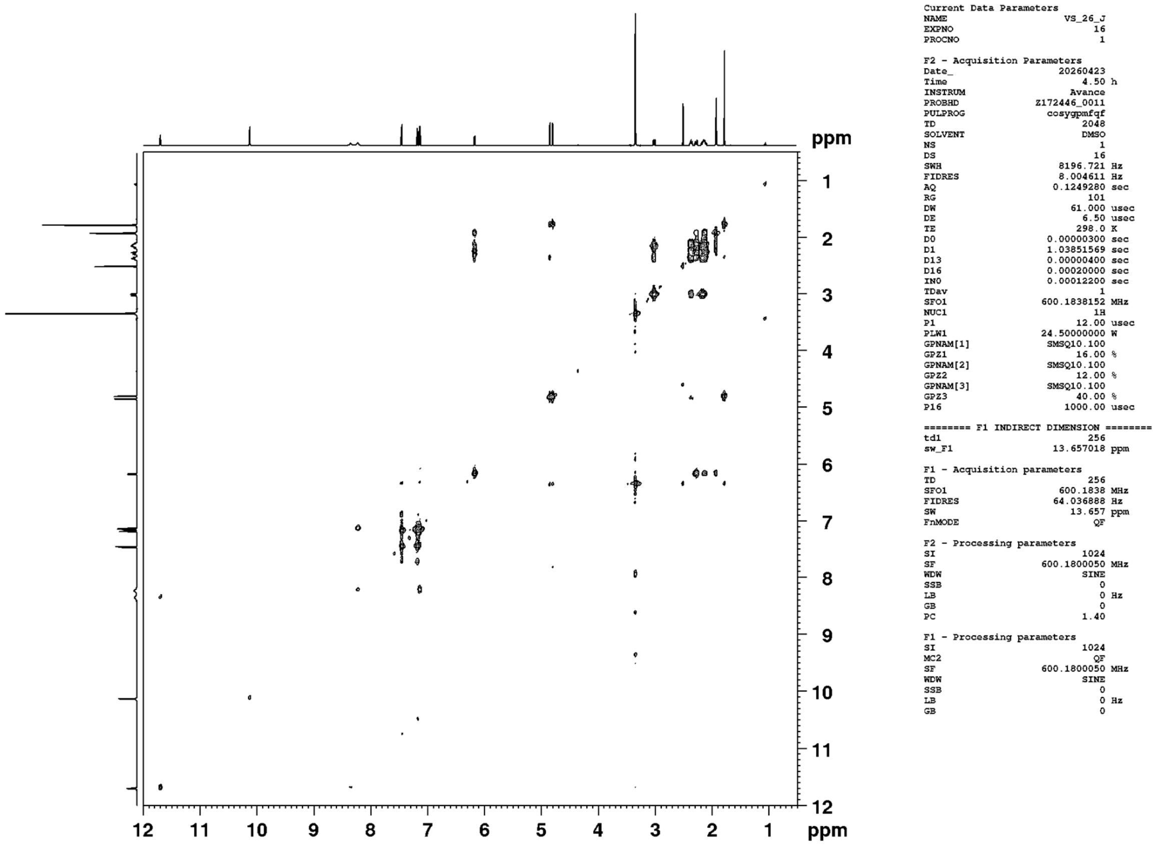


**Figure S52.** 2D COSY NMR spectrum of **3h** in DMSO-$d_6$.

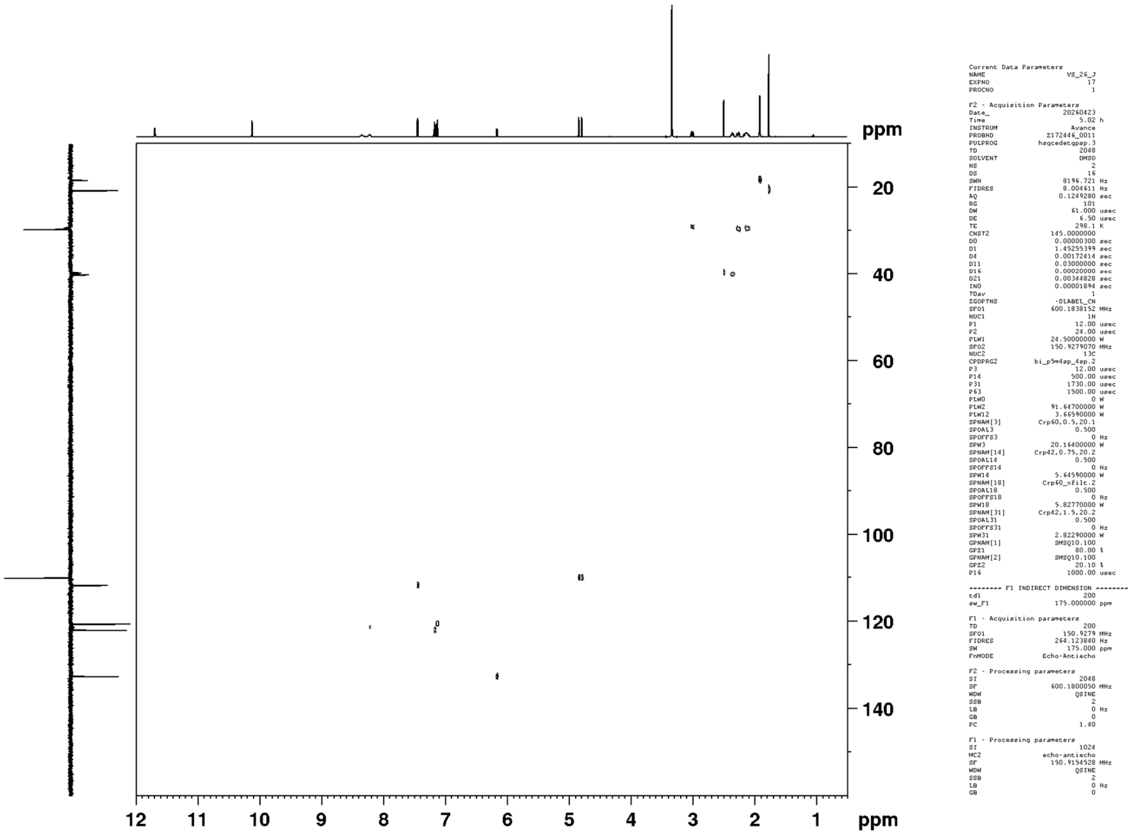


**Figure S53.** 2D HSQC NMR spectrum of **3h** in DMSO-$d_6$.

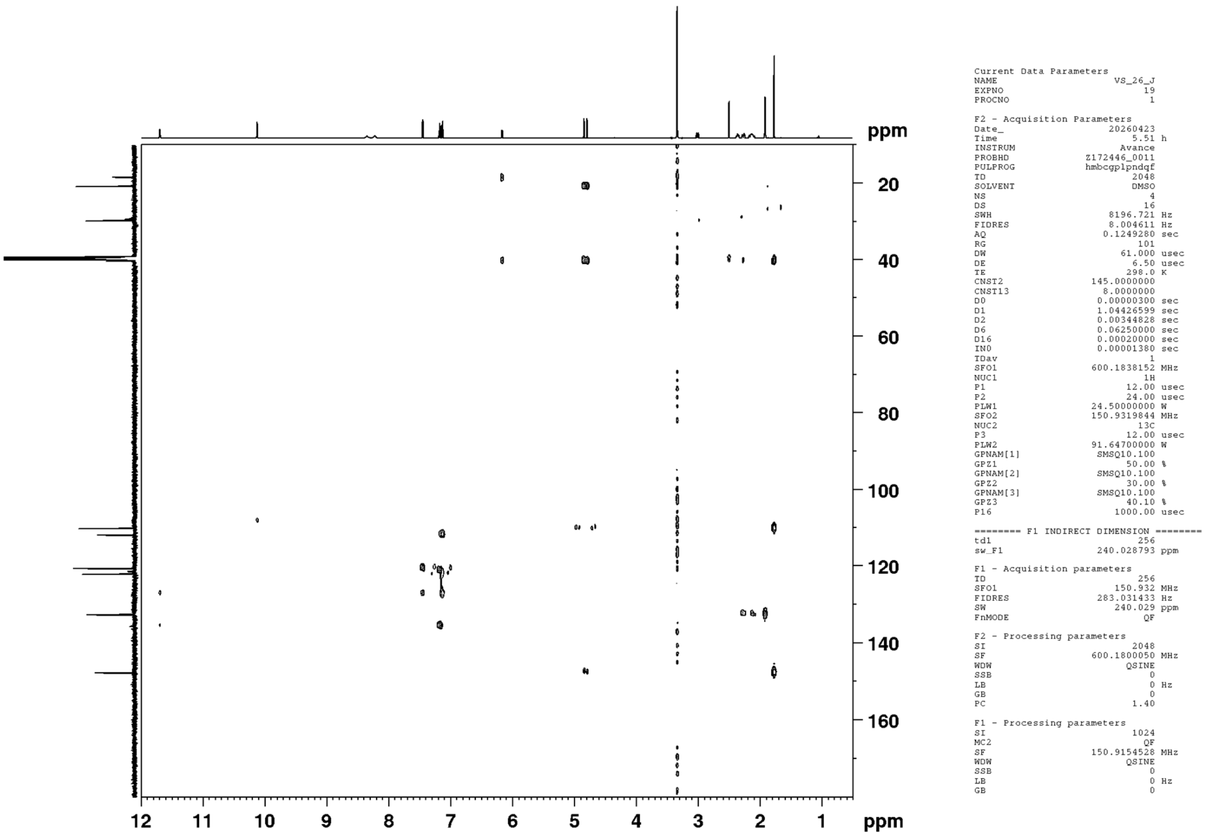


**Figure S54.** 2D HMBC NMR spectrum of **3h** in DMSO-$d_6$.

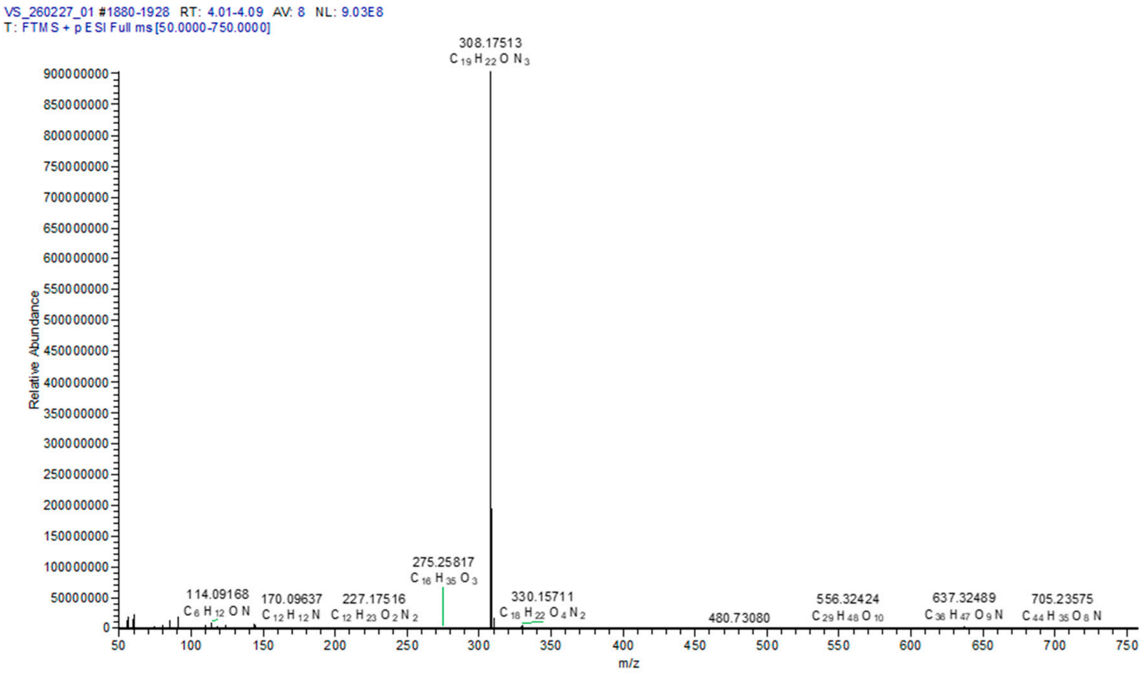


**Figure S55**. HRMS of **3h**.

9. (*R*,*E*)-*N'*-2-methyl-5-(prop-1-en-2-yl)cyclohex-2-en-1-ylidene)isonicotinohydrazide, **3i**

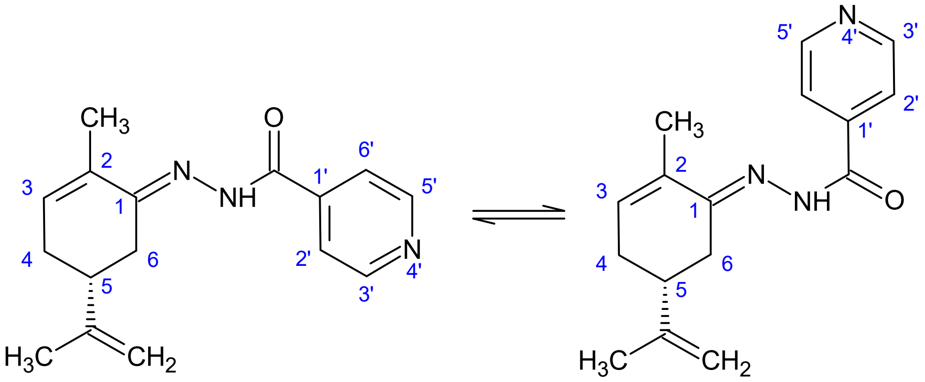


*Syn* *Anti*

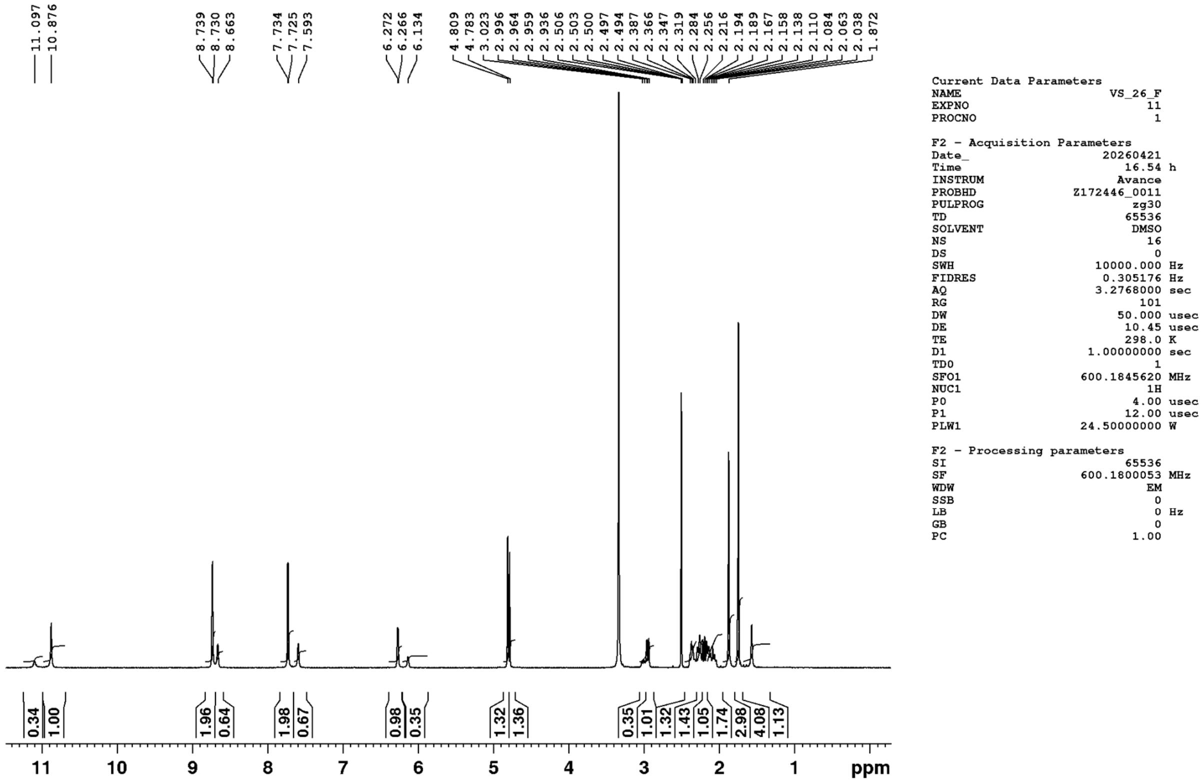


**Figure S56.** $^{1}H$ NMR spectrum of **3i** in DMSO-$d_6$.

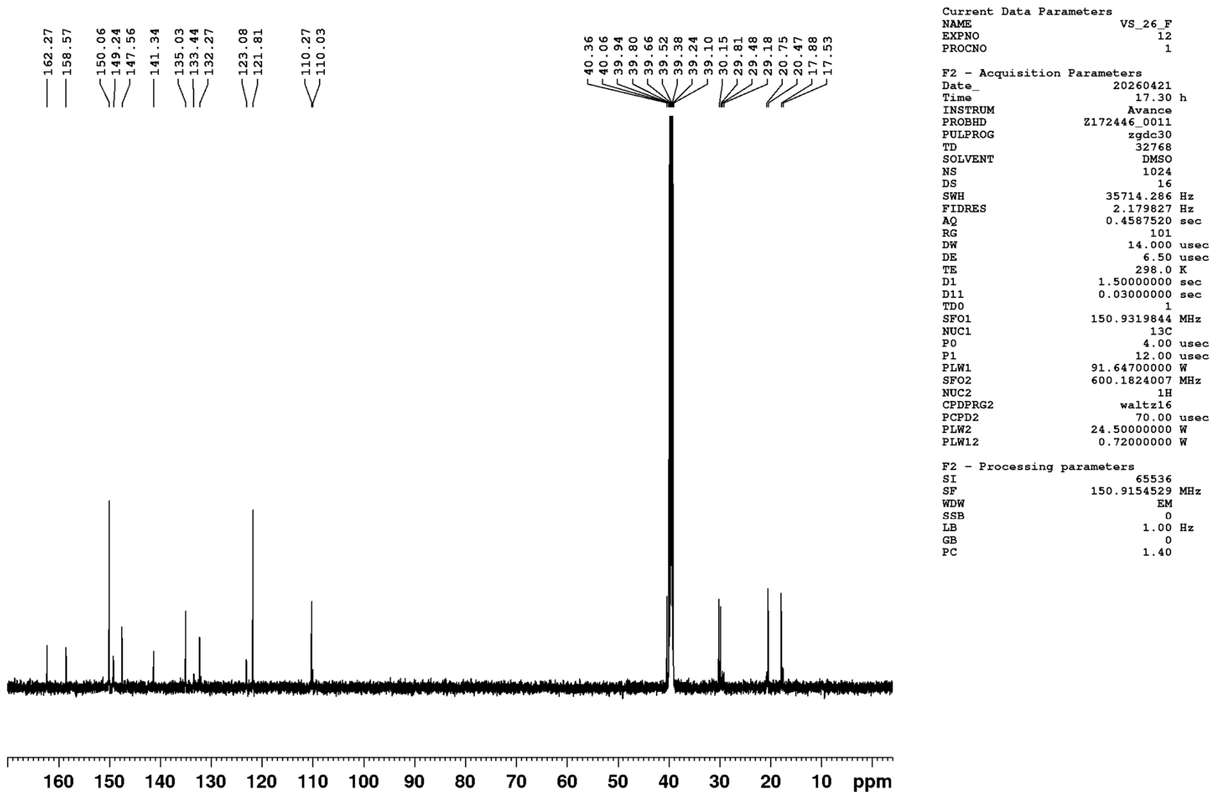


**Figure S57.** $^{13}C$ NMR spectrum of **3i** in DMSO-$d_6$.

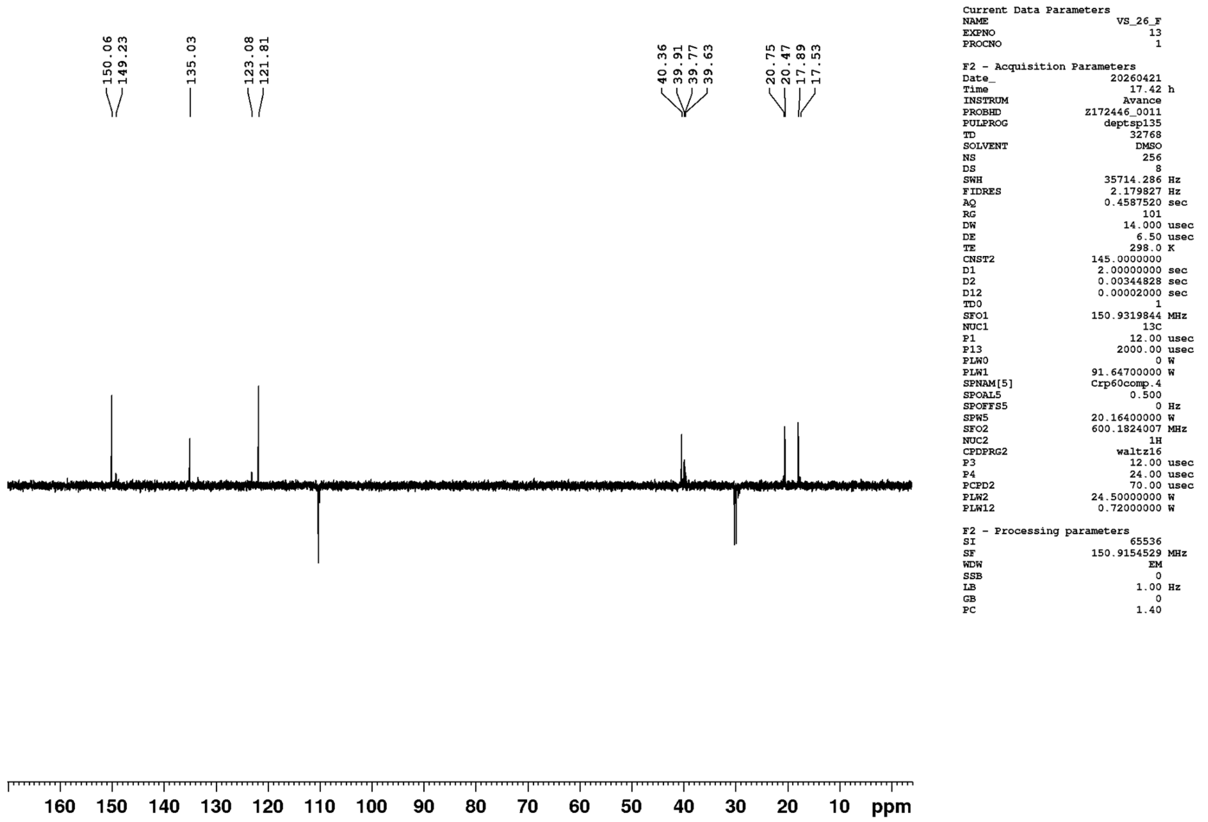


**Figure S58.** DEPT-135 NMR spectrum of **3i** in DMSO-$d_6$.

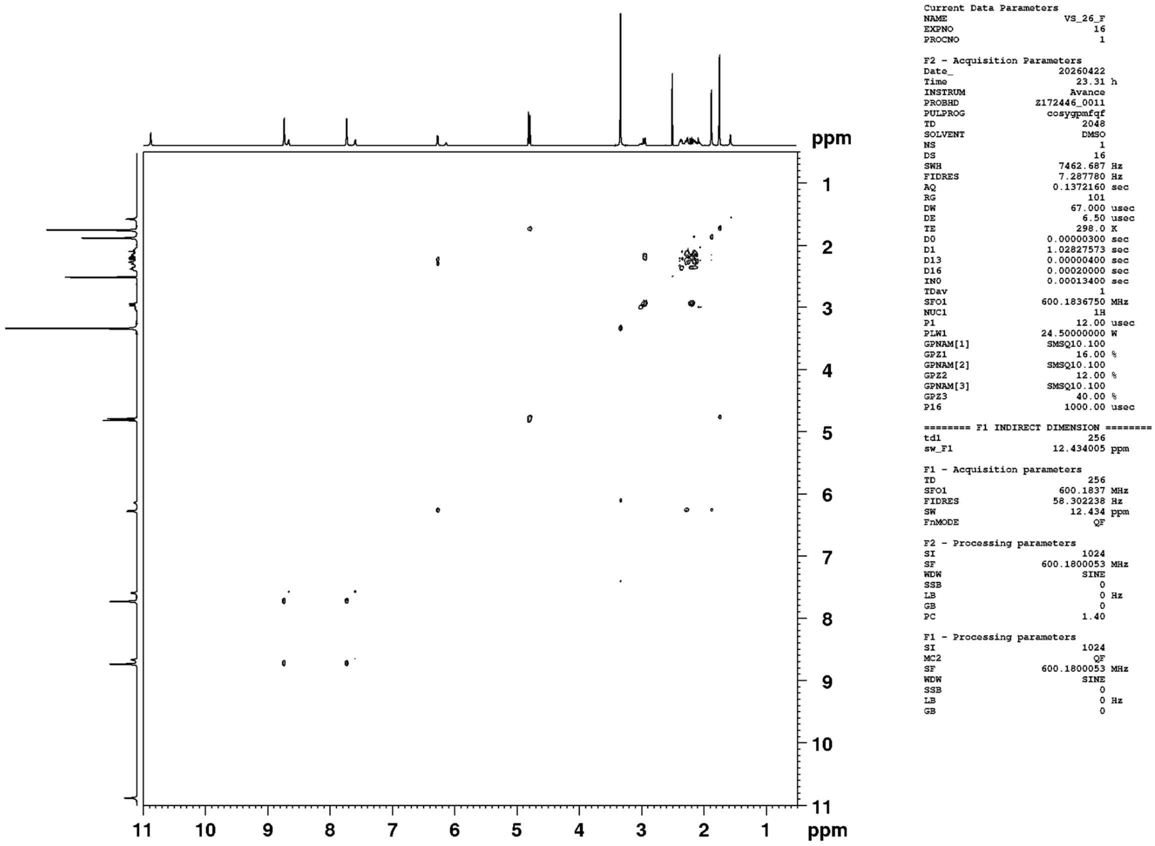


**Figure S59.** 2D COSY NMR spectrum of **3i** in DMSO-$d_6$.

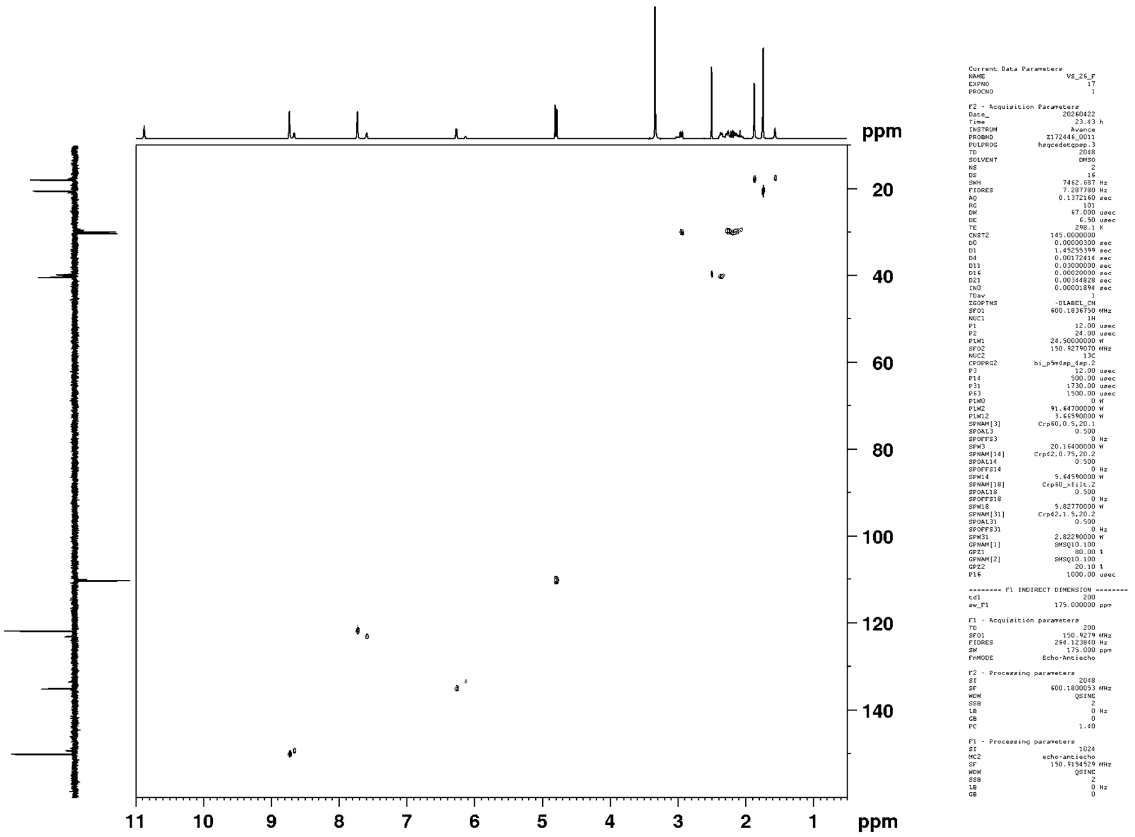


**Figure 60S.** 2D HSQC NMR spectrum of **3i** in DMSO-$d_6$.

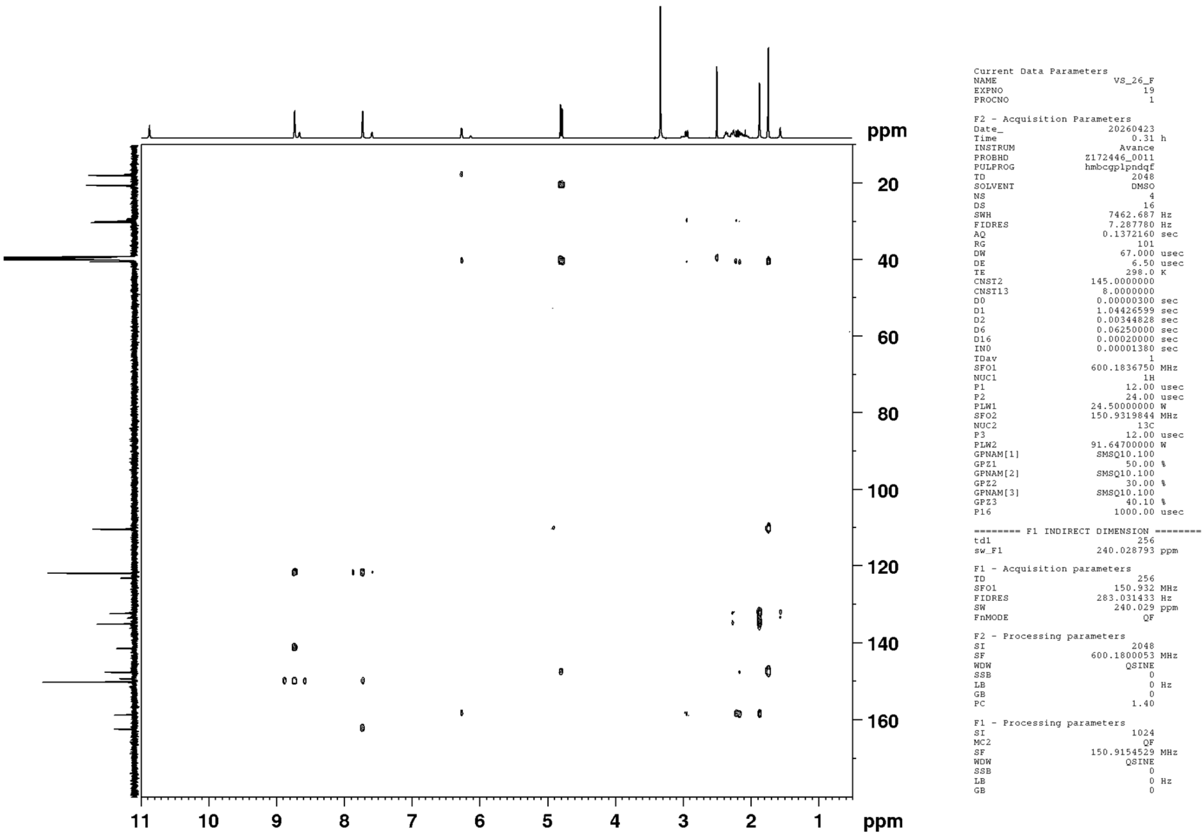


**Figure S61.** 2D HMBC NMR spectrum of **3i** in DMSO-$d_6$.

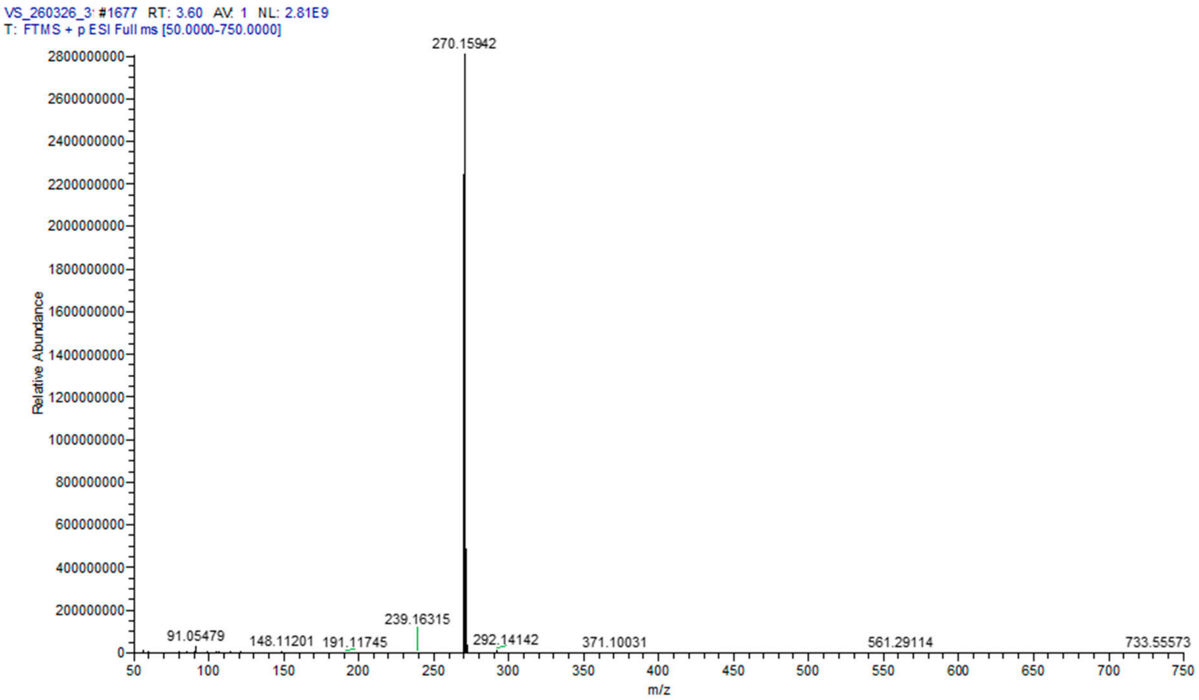


**Figure S62**. HRMS of **3i**.

**Table S1**. Hydrogen Bonds and weak interactions for **3i**.

| D | H | A | d(D-H)/Å | d(H-A)/Å | d(D-A)/Å | D-H-A/° |
|---|---|---|---|---|---|---|
| C61 | H61 | O82[1] | 0.93 | 2.60 | 3.172(4) | 120.4 |
| C61 | H61 | N102[1] | 0.93 | 2.57 | 3.493(4) | 174.4 |
| C162 | H16A | O81 | 0.97 | 2.61 | 3.082(4) | 110.5 |
| C162 | H16A | N101 | 0.97 | 2.52 | 3.481(4) | 168.4 |
| C161 | H16C | N102[1] | 0.97 | 2.55 | 3.476(4) | 159.1 |
| N91 | H91 | O82[1] | 0.95(3) | 2.01(3) | 2.941(3) | 166(3) |
| N92 | H92 | O81 | 0.88(3) | 2.04(3) | 2.904(3) | 167(3) |

Symmetry operation: [1] *1-x*, *1/2+y*, *1-z*

**Table S2**. Bond lengths for **3i**.

| Atom | Atom | Length/Å | Atom | Atom | Length/Å |
|---|---|---|---|---|---|
| O82 | C72 | 1.228(3) | C162 | C152 | 1.524(4) |
| O81 | C71 | 1.228(3) | C122 | C132 | 1.326(5) |
| N101 | N91 | 1.395(3) | C122 | C172 | 1.512(5) |
| N101 | C111 | 1.287(4) | N41 | C51 | 1.322(5) |
| N91 | C71 | 1.346(4) | N41 | C31 | 1.319(6) |
| N92 | N102 | 1.388(3) | C62 | C52 | 1.379(5) |
| N92 | C72 | 1.344(4) | C152 | C182 | 1.514(5) |
| N102 | C112 | 1.291(4) | C152 | C142 | 1.505(5) |
| C11 | C71 | 1.492(4) | C131 | C121 | 1.330(5) |
| C11 | C61 | 1.375(4) | C131 | C141 | 1.486(5) |
| C11 | C21 | 1.376(5) | N42 | C52 | 1.328(5) |
| C12 | C22 | 1.381(4) | C121 | C171 | 1.499(5) |
| C12 | C72 | 1.490(4) | C182 | C192 | 1.399(6) |
| C12 | C62 | 1.377(4) | C182 | C202 | 1.378(6) |
| C61 | C51 | 1.385(5) | C181 | C151 | 1.503(5) |
| C112 | C162 | 1.494(4) | C181 | C201 | 1.337(6) |
| C112 | C122 | 1.470(4) | C181 | C191 | 1.488(6) |
| C22 | C32 | 1.379(5) | C151 | C141 | 1.519(5) |
| C111 | C121 | 1.470(4) | C151 | C161 | 1.523(5) |
| C111 | C161 | 1.504(4) | C132 | C142 | 1.488(5) |
| C32 | N42 | 1.319(5) | C21 | C31 | 1.363(5) |

**Table S3**. Bond angles for **3i**.

| Atom | Atom | Atom | Angle/° | Atom | Atom | Atom | Angle/° |
|---|---|---|---|---|---|---|---|
| C111 | N101 | N91 | 117.9(2) | C132 | C122 | C172 | 122.4(3) |
| C71 | N91 | N101 | 117.1(2) | C31 | N41 | C51 | 115.9(4) |
| C72 | N92 | N102 | 118.3(2) | C12 | C62 | C52 | 119.2(3) |
| C112 | N102 | N92 | 117.2(2) | C182 | C152 | C162 | 110.4(3) |
| C61 | C11 | C71 | 125.4(3) | C142 | C152 | C162 | 109.6(3) |
| C61 | C11 | C21 | 117.1(3) | C142 | C152 | C182 | 114.8(3) |
| C21 | C11 | C71 | 117.6(3) | C121 | C131 | C141 | 125.6(3) |
| C22 | C12 | C72 | 123.9(3) | C32 | N42 | C52 | 115.1(3) |
| C62 | C12 | C22 | 117.3(3) | C111 | C121 | C171 | 118.6(3) |
| C62 | C12 | C72 | 118.7(3) | C131 | C121 | C111 | 119.4(3) |
| O81 | C71 | N91 | 123.6(3) | C131 | C121 | C171 | 121.9(3) |
| O81 | C71 | C11 | 118.9(3) | C192 | C182 | C152 | 120.4(4) |
| N91 | C71 | C11 | 117.4(2) | C202 | C182 | C152 | 118.8(4) |
| C11 | C61 | C51 | 118.8(3) | C202 | C182 | C192 | 120.8(4) |
| N102 | C112 | C162 | 125.1(3) | C201 | C181 | C151 | 120.1(4) |
| N102 | C112 | C122 | 116.9(3) | C201 | C181 | C191 | 121.6(4) |
| C122 | C112 | C162 | 117.8(3) | C191 | C181 | C151 | 118.3(4) |
| C32 | C22 | C12 | 118.4(3) | N41 | C51 | C61 | 124.2(3) |
| O82 | C72 | N92 | 124.3(3) | C181 | C151 | C141 | 114.0(3) |
| O82 | C72 | C12 | 119.9(3) | C181 | C151 | C161 | 112.4(3) |
| N92 | C72 | C12 | 115.8(2) | C141 | C151 | C161 | 109.9(3) |
| N101 | C111 | C121 | 116.0(3) | C122 | C132 | C142 | 125.3(3) |
| N101 | C111 | C161 | 127.0(3) | C131 | C141 | C151 | 111.7(3) |
| C121 | C111 | C161 | 117.0(3) | C31 | C21 | C11 | 119.7(4) |
| N42 | C32 | C22 | 125.4(3) | C111 | C161 | C151 | 110.9(3) |
| C112 | C162 | C152 | 114.4(3) | N42 | C52 | C62 | 124.5(4) |
| C112 | C122 | C172 | 118.3(3) | C132 | C142 | C152 | 110.7(3) |
| C132 | C122 | C112 | 119.1(3) | N41 | C31 | C21 | 124.4(4) |

**Table S4**. Torsion angles for **3i**.

| A | B | C | D | Angle/° | A | B | C | D | Angle/° |
|---|---|---|---|---|---|---|---|---|---|
| N101 | N91 | C71 | O81 | 7.2(4) | C32 | N42 | C52 | C62 | -1.2(7) |
| N101 | N91 | C71 | C11 | -172.1(2) | C162 | C112 | C122 | C132 | -6.1(5) |
| N101 | C111 | C121 | C131 | -172.9(3) | C162 | C112 | C122 | C172 | 178.0(3) |
| N101 | C111 | C121 | C171 | 9.4(5) | C162 | C152 | C182 | C192 | -73.9(5) |
| N101 | C111 | C161 | C151 | 142.0(3) | C162 | C152 | C182 | C202 | 104.4(4) |
| N91 | N101 | C111 | C121 | -175.6(3) | C162 | C152 | C142 | C132 | -51.1(4) |
| N91 | N101 | C111 | C161 | 3.6(5) | C122 | C112 | C162 | C152 | -22.5(4) |
| N92 | N102 | C112 | C162 | 2.2(5) | C122 | C132 | C142 | C152 | 25.5(6) |
| N92 | N102 | C112 | C122 | -172.6(3) | C62 | C12 | C22 | C32 | -0.5(5) |
| N102 | N92 | C72 | O82 | 3.9(5) | C62 | C12 | C72 | O82 | -37.4(4) |
| N102 | N92 | C72 | C12 | -174.4(2) | C62 | C12 | C72 | N92 | 141.0(3) |
| N102 | C112 | C162 | C152 | 162.8(3) | C121 | C111 | C161 | C151 | -38.8(4) |
| N102 | C112 | C122 | C132 | 169.0(3) | C121 | C131 | C141 | C151 | 16.6(6) |
| N102 | C112 | C122 | C172 | -6.9(5) | C182 | C152 | C142 | C132 | -176.0(3) |
| C11 | C61 | C51 | N41 | -0.3(6) | C181 | C151 | C141 | C131 | -173.8(4) |
| C11 | C21 | C31 | N41 | -3.4(8) | C181 | C151 | C161 | C111 | -173.9(3) |
| C12 | C22 | C32 | N42 | -0.6(6) | C51 | N41 | C31 | C21 | 3.1(8) |
| C12 | C62 | C52 | N42 | 0.2(7) | C141 | C131 | C121 | C111 | 3.8(6) |
| C71 | C11 | C61 | C51 | -178.9(3) | C141 | C131 | C121 | C171 | -178.6(4) |
| C71 | C11 | C21 | C31 | -179.3(4) | C141 | C151 | C161 | C111 | 58.0(3) |
| C61 | C11 | C71 | O81 | 155.8(3) | C21 | C11 | C71 | O81 | -23.3(4) |
| C61 | C11 | C71 | N91 | -24.9(4) | C21 | C11 | C71 | N91 | 156.0(3) |
| C61 | C11 | C21 | C31 | 1.6(6) | C21 | C11 | C61 | C51 | 0.1(5) |
| C112 | C162 | C152 | C182 | 178.5(3) | C161 | C111 | C121 | C131 | 7.8(5) |
| C112 | C162 | C152 | C142 | 51.1(4) | C161 | C111 | C121 | C171 | -169.9(3) |
| C112 | C122 | C132 | C142 | 4.6(6) | C161 | C151 | C141 | C131 | -46.6(4) |
| C22 | C12 | C72 | O82 | 141.1(3) | C142 | C152 | C182 | C192 | 50.5(5) |
| C22 | C12 | C72 | N92 | -40.5(4) | C142 | C152 | C182 | C202 | -131.1(4) |
| C22 | C12 | C62 | C52 | 0.6(5) | C172 | C122 | C132 | C142 | -179.7(4) |
| C22 | C32 | N42 | C52 | 1.4(6) | C31 | N41 | C51 | C61 | -1.2(7) |
| C72 | N92 | N102 | C112 | 172.1(3) | C201 | C181 | C151 | C141 | -118.6(4) |
| C72 | C12 | C22 | C32 | -179.0(3) | C201 | C181 | C151 | C161 | 115.5(4) |
| C72 | C12 | C62 | C52 | 179.2(3) | C191 | C181 | C151 | C141 | 62.7(5) |
| C111 | N101 | N91 | C71 | -171.9(3) | C191 | C181 | C151 | C161 | -63.2(5) |
